# Thermally Activated Delayed Fluorescence from d^10^‐Metal Carbene Complexes through Intermolecular Charge Transfer and Multicolor Emission with a Monomer–Dimer Equilibrium

**DOI:** 10.1002/chem.202004106

**Published:** 2020-11-18

**Authors:** Lei Cao, Shiqing Huang, Wei Liu, Hongyan Zhao, Xiao‐Gen Xiong, Jian‐Ping Zhang, Li‐Min Fu, Xiaoyu Yan

**Affiliations:** ^1^ Department of Chemistry Renmin University of China Beijing 100872 P.R. China; ^2^ Sino-French Institute for Nuclear Energy and Technology Sun Yat-sen University Guangzhou 510275 P.R. China

**Keywords:** donor–acceptor systems, dual-emission systems, charge transfer, emission, fluorescence

## Abstract

A series of two‐coordinate Au^I^ and Cu^I^ complexes (**3 a**, **3 b** and **5 a**, **5 b**) are reported as new organometallic thermally activated delayed fluorescence (TADF) emitters, which are based on the carbene–metal–carbazole model with a pyridine‐fused 1,2,3‐triazolylidene (PyTz) ligand. PyTz features low steric hindrance and a low‐energy LUMO (LUMO=−1.47 eV) located over the π* orbitals of the whole ligand, which facilitates intermolecular charge transfer between a donor (carbazole) and an accepter (PyTz). These compounds exhibit efficient TADF with microsecond lifetimes. Temperature‐dependent photoluminescence kinetics of **3 a** supports a rather small energy gap between S_1_ and T_1_ (Δ*E* ${}_{S{}_{1}-T{}_{1}}$ =60 meV). Further experiments reveal that there are dual‐emission properties from a monomer–dimer equilibrium in solution, exhibiting single‐component multicolor emission from blue to orange, including white‐light emission.

## Graphical abstract

**Act later**: This innovative work has developed a dimeric model to construct organometallic through‐space charge‐transfer thermally activated delayed fluorescence emitters, as well as single‐component multicolor luminescence, based on two‐coordinate carbene–metal–carbazole complexes.

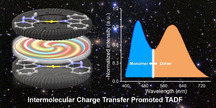

## Introduction

**Figure 1:**
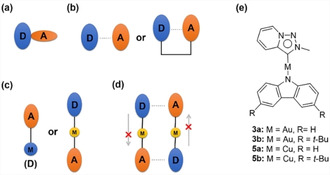
a) TBCT twisted D–A model. b) TSCT intermolecular (left) and intramolecular (right) D–A model. c) Through‐bond MLCT (left) and LLCT (right) metal complex model. d) TSCT based on the linear model of D‐M‐A (this work). e) Molecular structures of **3 a**, **3 b**, **5 a**, and **5 b**. (D: donor. A: acceptor. M: metal.)

In the past few years, thermally activated delayed fluorescence (TADF) emitters have been regarded as a new generation of luminous materials after fluorescent and phosphorescent emitters because of the efficient upconversion from nonemissive triplet states (T_1_) to fluorescent singlet states (S_1_) by thermally activated reverse intersystem crossing (RISC), which could theoretically achieve 100 % internal quantum efficiency (IQE).^[1]^ The current TADF molecular designs include two strategies: 1) through‐bond charge transfer (TBCT) in a twisted donor–acceptor (D–A) configuration that minimizes the overlap between the HOMO and LUMO, resulting in a small Δ*E*_ST_ (<0.1 eV; Figure 1 a);[^1a^, ^2^] and 2) a through‐space charge transfer (TSCT) inter‐/intramolecular D–A configuration that relies on a pair of electron‐donor and ‐acceptor units (Figure 1 b).[^1b^, ^3^] Notably, for TADF organometallic emitters,^[4]^ the common transitions are limited to intramolecular through‐bond metal‐to‐ligand charge transfer (MLCT) or ligand‐to‐ligand charge transfer (LLCT) with spatial separation of the HOMO and LUMO, which leads to a small Δ*E*_ST_ (Figure 1 c).^[4b]^ This means these TADF emitters might be affected by disturbance of the aggregate and excimer formed in the nondoping systems or fluid processes.

Luminescent Group 11 N‐heterocyclic carbene (NHC) complexes have been most extensively studied among all transition‐metal–NHC complexes.^[5]^ Recently, there have been several reports on organometallic TADF emitters based on the model of carbene–d^10^ metal–amides, wherein carbenes with strong π‐accepting abilities played a crucial role in TADF characteristics.^[6]^ The lowest‐energy charge transition of this family of complexes was mainly concentrated on interligand charge transfer (LLCT) from donor (amide) to acceptor (carbene), due to the coplanarity of the ligands and the contribution of metal d orbitals to an electronic bridge. Apart from intramolecular D–A charge transfer (CT), this molecular model provides another possibility for realizing intermolecular D–A interactions (Figure 1 d).

1,2,3‐Triazolylidenes, a class of mesoionic carbenes (MICs), have attracted tremendous attention.^[7]^ However, compared with other traditional NHCs, 1,2,3‐triazolylidenes exhibit poorer π‐accepting ability and extremely low‐energy LUMOs, which are mainly located on the π* orbitals of the triazole ring; thus, this kind of carbene ligand has good potential for the construction of novel TADF metal complexes through intermolecular CT. We envisioned that the LUMO energy could be further lowered by fusing a pyridine ring. In this regard, we report a class of two‐coordinate, neutral PyTz–M–Cz complexes **3 a**, **3 b**, **5 a**, and **5 b** (PyTz=pyridine‐fused 1,2,3‐triazolylidene; M=Au^I^, Cu^I^; Cz=carbazole; Figure 1 e), in which PyTz exhibits weak π‐accepting ability but low‐energy LUMO. With a metal bridge connecting D and A, these molecules are designed to have intermolecular CT between PyTz and Cz, which allows such complexes to emit efficient TADF with short lifetimes in the range of 0.6–2.4 μs in the solid state. Next, investigations into the self‐assembly behavior and photophysical properties of these complexes in solution demonstrated the existence of a monomer–dimer equilibrium, in which the dimer exhibited TADF characteristics at around 600 nm. Additionally, based on the monomer–dimer equilibrium and TADF characteristics, this dual‐emission system can be switched to exhibit single‐component multicolor emission over a wide range, from blue (450 nm) to orange (600 nm), including white‐light emission.^[8]^

## Results and Discussion

### Synthesis and structural characterization

**Scheme 1:**
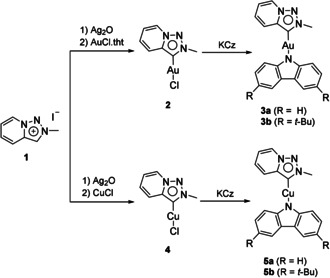
Synthetic routes to complexes **3 a**, **3 b**, **5 a**, and **5 b**. AuCl**⋅**tht: chloro(tetrahydrothiophene)gold(I).

**Figure 2:**
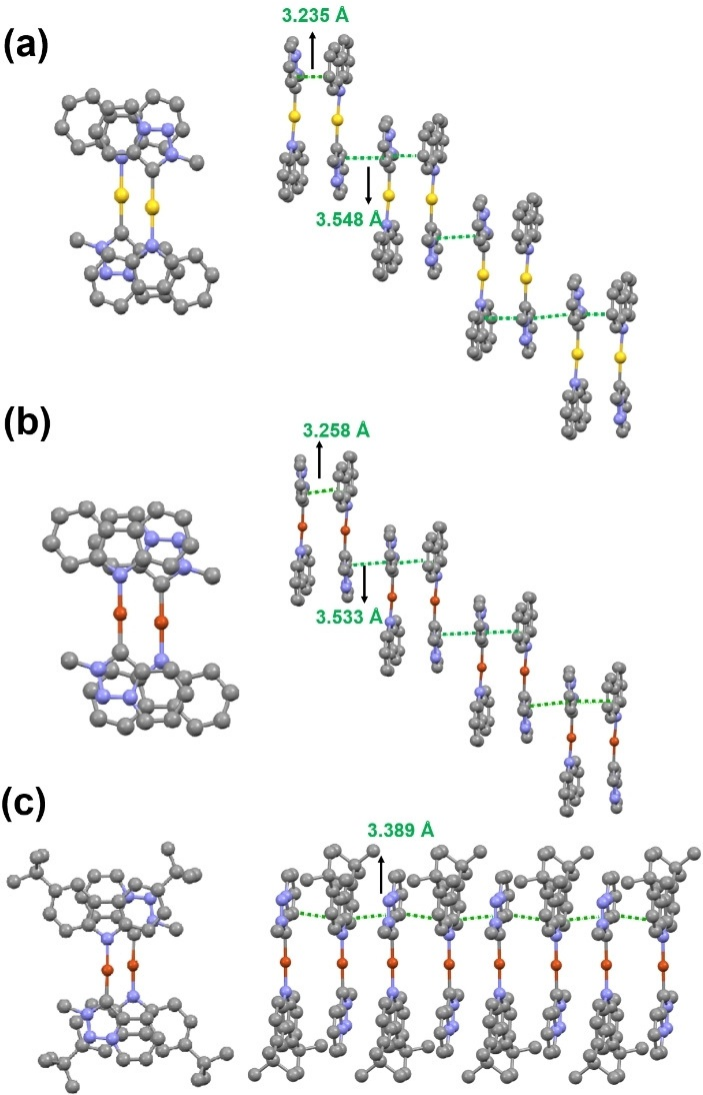
Crystal structures of **3 a**, **5 a**, and **5 b** and the corresponding packing mode. Left: dimeric configurations of a) **3 a**, b) **5 a**, and c) **5 b**; right: packing modes of a) **3 a**, b) **5 a**, and c) **5 b** in space.

PyTz–M–Cz complexes **3** and **5** were synthesized from the carbene precursor, pyridine‐fused 1,2,3‐triazolium (**1**; Scheme 1). Single crystals of **3 a**, **5 a**, and **5 b** have been obtained by diffusion of diethyl ether into a concentrated solution of the complex in dichloromethane for structure determination by means of X‐ray crystallography. X‐ray crystallography of **3** and **5** indicates that all of the molecules present nearly planar structures. For **3 a**, the dihedral angle between the planes upon which PyTz and Cz are located is only 4.61°, whereas, for **5 a** and **5 b**, the dihedral angles are 5.91 and 0°, respectively (Table S7 in the Supporting Information). Furthermore, these complexes could self‐assemble to form a head‐to‐tail dimeric conformation (Scheme 1 and Figure 2). With regard to **3 a** and **5 a**, each molecule is connected to another, forming a dimer in a head‐to‐tail conformation, in which the electron‐rich Cz ring faces toward the electron‐deficient PyTz ligand. The distances of the π–π interactions inside the dimers are around 3.235 and 3.258 Å, respectively. The π–π distances between dimers are about 3.548 Å for **3 a** and 3.533 Å for **5 a**. Then, for **5 b**, a similar self‐assembly mode of the molecule could be observed (in a head‐to‐tail manner). However, unlike **3 a** and **5 a**, molecules of **5 b** are linked to each other through π–π interactions, forming 1D chain structures; the distance of the π–π interaction is around 3.389 Å. Furthermore, in the single‐crystal structure of **5 b**, Cz is not completely facing PyTz, but is slightly interlaced with the carbene ligand, which is probably due to steric hindrance from the *tert*‐butyl groups (Figure 2). It is worth noting that there is no clear metal–metal interaction among complexes (M–M distances >3.5 Å). Selected bond lengths and angles in complexes **3 a** and **5 a**–**5 b** are collected in Table S7 in the Supporting Information.

### Monomer–dimer equilibrium studies

**Figure 3:**
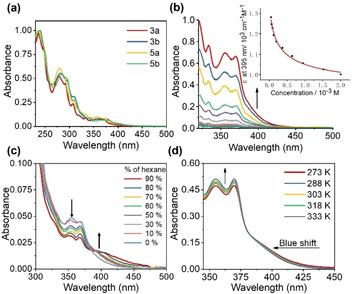
Room‐temperature UV/Vis absorption spectra of a) complexes **3 a**, **3 b**, **5 a** and **5 b** in THF; b) complex **3 a** in THF at different concentrations (1×10^−5^–2×10^−3^ m); inset: Dimerization plots for the monomer–dimer equilibrium monitored at the absorption wavelength of the dimer; and c) complex **3 a** in THF with different volume fractions of hexane (0–90 %); [**3 a**]=1.5×10^−5^ m. d) UV/Vis absorption spectra of **3 a** in THF at various temperatures; [**3 a**]=1×10^−3^ m.

Figure 3 a shows the absorption spectra of **3 a**, **3 b**, **5 a** and **5 b** in THF, and Table 1 summarizes the photophysical properties of these complexes. All of the complexes exhibit similar spectral profiles, although *tert*‐butylated complexes **3 b** and **5 b** show bathochromic shifts of about 6 nm with respect to their non‐butylated counterparts. Structured absorption bands between 280 and 310 nm are assigned to intraligand π→π* and n→π* transitions of Cz and PyTz, whereas the low‐energy absorption bands at 358–380 nm are also attributed to the Cz ligand, which are consistent with the absorption spectra of the KCz and KCz′′ (Figure S7 in the Supporting Information), suggesting that there are no MLCT or LLCT transitions for such complexes. Notably, all of the complexes display weak absorption tails at around 400 nm; in view of the single‐crystal data, these might be attributed to the formation of a dimer through π–π interactions. On the basis of the concentration‐dependent UV/Vis spectra (Figure 3 b and Figure S8 in the Supporting Information), the dimerization constant *K* (THF, 25 °C) was determined by applying absorbance plots at around 400 nm to a monomer–dimer equilibrium (see “Calculations of the dimerization constant *K* using absorption spectra” in the Supporting Information).^[9]^ The dimerization constants, *K*, and the corresponding Gibbs free energies (Δ*G*) of all complexes have been collected in Table 1.

**Table 1:** Electronic absorption data, dimerization constants (log *K*), and Gibbs free energies (Δ*G*) of complexes **3 a**, **3 b**, **5 a**, and **5 b** in THF at room temperature.

Complex	*λ*_max_ [nm] (*ϵ* [dm^3^ mol^−1^ cm^−1^])	Log *K* ^[a]^	Δ*G* ^[b]^ [kcal mol^−1^]
**3 a**	278 (18 340), 358 (2750), 370 (2564)	3.44	−4.69
**3 b**	281 (17 707), 362 (2551), 376 (2608)	3.64	−4.96
**5 a**	279 (25 348), 359 (3984), 375 (3552)	3.54	−4.83
**5 b**	285 (16 524), 365 (2027), 380 (2003)	3.67	−5.00

[a] Dimerization constants determined by means of UV/Vis absorption spectroscopy for solutions of the complexes (1×10^−5^ to 2×10^−3^ m) in THF. [b] Δ*G* obtained from the dimerization constant, *K*.

For further proof of the existence of dimers in solution, investigations of induced self‐assembly were performed in mixed‐solvent systems. The nominal concentration of complexes **3 a**, **3 b**, **5 a**, and **5 b** was kept at 1.5×10^−5^ m, and hexane was added to trigger aggregation (Figure 3 c and Figure S9 in the Supporting Information). Upon addition of hexane (0 to 90 %) to the solution of **3 a** in THF, an isosbestic point appeared at around 390 nm, with the growth of an absorption shoulder at around 405 nm and a decline of the absorption band at 375 nm. It is worth noting that increasing the proportion of the poor solvent only led to growth of the shoulder at around 400 nm and no other new absorption bands were observed. These observations suggest that the series of complexes undergo solvent‐induced aggregation, which favors the dimer upon the addition of a poor solvent.^[10]^

The temperature‐dependent UV/Vis spectra of complex **3 a** in THF are shown in Figure 3 d. With increasing temperature, there was a blueshift of the band initially centered at about 400 nm, whereas the absorption of Cz at 365 nm was gradually enhanced, indicating that increased molecular motions weakened the intermolecular CT process at high temperature.^[11]^ In addition, the ^1^H–^1^H COSY spectrum of **3 b** shows a cross peak between the protons of PyTz and proton of Cz (Figure S10 in the Supporting Information), which indicates that head‐to‐tail π–π interactions are conserved in CDCl_3_.

**Figure 4:**
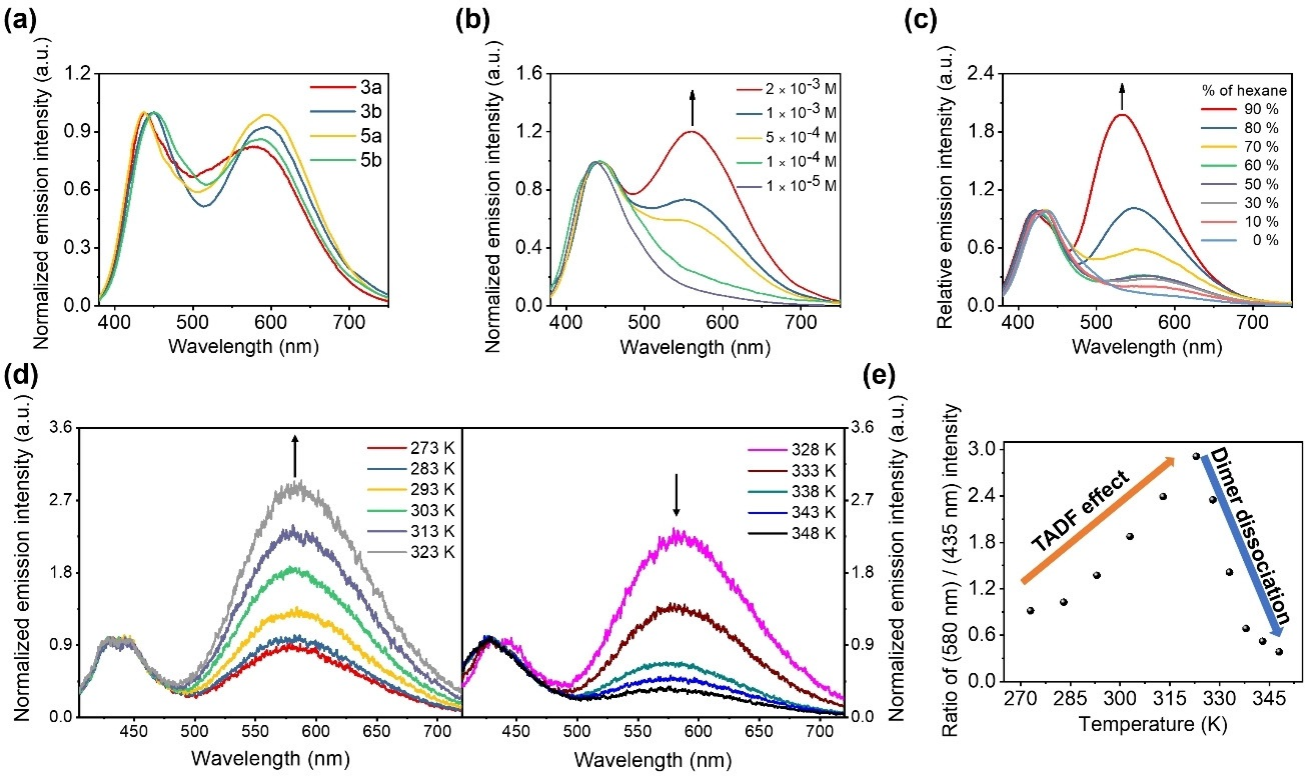
Room‐temperature PL spectra of a) complexes **3 a**, **3 b**, **5 a**, and **5 b** at 1×10^−3^ m in THF; *λ*_ex_=375 nm; b) complex **3 a** in THF at different concentrations (1×10^−5^‐2×10^−3^ m); intensity maxima of the blue emission bands were normalized; *λ*_ex_=375 nm; c) complex **3 a** in THF mixed with hexane at different volume fractions (0–90 %); intensity maxima of the blue emission bands were normalized; [**3 a**]=1.5×10^−5^ m, *λ*_ex_=375 nm. d) PL spectra of complex **3 a** in THF recorded at different temperatures, ranging from 0 to 50 °C (left) and 55 to 75 °C (right); intensity maxima of the blue emission bands were normalized. e) PL intensity ratio of the dimer over the monomer as a function of temperature; [**3 a**]=1.0×10^−3^ m; *λ*_ex_=380 nm.

Photoluminescence (PL) spectra of **3 a**, **3 b**, **5 a**, and **5 b** in the nondoped solid state exhibit broad emission bands centered at 539–580 nm (Table 2 and Figure S11 in the Supporting Information). However, emission spectra of these complexes in THF exhibit dual‐emission bands at around 450 and 600 nm (Table 2 and Figure 4 a). The high‐energy, narrow, emission bands at around 450 nm are attributed to the π→π* transition of ^1^Cz, which are in line with the emission of KCz and KCz′′ that display vibronic fine structure (Figure S12 in the Supporting Information). To investigate the origin of the orange‐colored emission band (≈600 nm) thoroughly, a concentration‐dependent PL experiment was first carried out (Figure 4 b and Figure S13 in the Supporting Information). The intensity of the emission band depends on the concentration of the solution. According to single‐crystal data and UV/Vis absorption investigation, this emission band was produced by the formation of a dimer. The self‐assembly behavior of all complexes in THF/hexane mixtures were further investigated (Figure 4 c and Figure S14 in the Supporting Information). All of the complexes were prepared at a dilute concentration of 1.5×10^−5^ m in the admixture solution, of which there was little emission at around 600 nm at the same concentration in pure THF. As the proportion of hexane gradually increased, the emission signal at longer wavelength became more intense than that at 450 nm. Notably, the position of the emission band of ^1^Cz at around 450 nm was not polarity dependent, but the emission band of the dimer underwent a hypochromatic shift of about 70 nm, going from more polar (hexane 0 %) to less polar (hexane 90 %) solutions.

**Table 2:** Photophysical data for complexes **3 a**, **3 b**, **5 a**, and **5 b** at room temperature.

Complex	Solution					Solid				
	*λ*_dual emission_ [nm]	*τ*_1_^[b]^ [ns]	*τ*_2,p_^[c]^ [ns] (*f* ^[d]^ [%])	*τ*_2,d_^[e]^ [ns] (*f* ^[d]^ [%])	*Φ*_RT_^[f]^	*λ*_emission_ [nm]	*τ*_d_^[g]^ [μs]	*Φ*_RT_^[f]^	*k*_r_ [s^−1^]	*k*_nr_ [s^−1^]
**3 a**	439, 582	3.6	6.7 (3)	182.5 (97)	0.32	580	0.9	0.49	5.4×10^5^	5.7×10^5^
**3 b**	449, 600	2.8	6.6 (7)	43.3 (93)	0.39	555	2.4	0.58	2.4×10^5^	1.8×10^5^
**5 a**	437, 594	4.0	4.4 (20)	68.7 (80)	0.11	570	0.6	0.26	4.3×10^5^	1.2×10^6^
**5 b**	451, 588	3.5	4.1 (16)	28.8 (84)	0.17	539	1.0	0.40	4.0×10^5^	6×10^5^

[a] Measured in THF (1×10^−3^ m) at room temperature. [b] Average PL lifetime at the shorter emission wavelength. [c] Prompt lifetimes at the longer emission wavelength. [d] The fraction of prompt or delayed components. [e] Delayed lifetimes at the longer emission wavelength. [f] Absolute quantum yield (QY) determined at room temperature by the use of an integrating sphere. [g] Delayed lifetimes measured in the solid state at room temperature.

### Computational studies

DFT calculations at the M062X/def2SVP level in the gas phase have been performed on the monomers and dimers of these complexes to investigate the effect of D–A interactions on the molecular geometry (Figures S16–S18 and Table S8 in the Supporting Information). The optimized ground structures of the monomers were in a twisted conformation between PyTz and Cz, whereas the dimeric models showed a planar conformation and were nearly consistent with the single‐crystal results. This difference was due to the existence of intermolecular noncovalent π–π interactions between Cz and PyTz, which propelled the complexes into a planar configuration.

**Figure 5:**
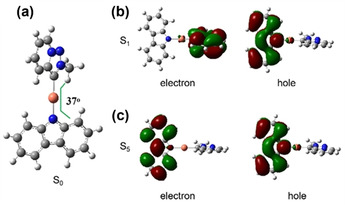
a) The optimized ground‐state structure of monomer **5 a**. The electron and hole distributions of b) the lowest‐energy excited state (S_1_) and c) the fifth‐lowest‐energy excited state (S_5_). The geometry of **5 a** is optimized at S_1_ by using TDDFT calculations.

Notably, the twisted conformation of the monomer plays a crucial role in the luminescence of metal complexes.[^4a^, ^6b^, ^12^] Time‐dependent (TD) DFT calculations of **5 a** showed that the S_1_ state originated from intramolecular CT from Cz to PyTz, but there was a negligible oscillator strength (*f*=0.0000) because of the poor overlap between electron and hole distributions, which was associated with the twisted geometry of the complex (Figure 5). The excited states S_1_ to S_4_ had very low oscillator strengths until S_5_ gave a larger oscillator strength (*f*=0.0450), which was mainly assigned to π→π* of Cz (93 %; Table S9 in the Supporting Information), showing the consistency of the emission of ^1^Cz observed in the dilute solution.

In addition, the electronic effect of the carbene ligand is also a key factor affecting the photophysical properties. Molecular orbital analysis demonstrates that, unlike other carbenes, in which LUMOs are mainly located on the unfilled p orbital of the carbene carbon, the LUMOs of PyTz and MIC are primarily distributed over the π* orbitals of the whole ligand and LUMO+2, which are mainly situated on the carbene carbon, truly reflect the π‐accepting ability of the carbene (Figures S19–S25 in the Supporting Information). The energy of LUMO+2 of PyTz (0.93 eV) is relatively higher than that of other carbenes. In fact, MLCT or intramolecular LLCT cannot be observed for this series of 1,2,3‐triazolylidene complexes.^[13]^ However, it is noteworthy that the low‐energy LUMO and lesser steric hindrance enable PyTz to have the potential to facilitate intermolecular CT.

### TADF studies

To probe the nature of dual emission, a temperature‐dependent PL experiment has been carried out (Figure 4 d and Figure S26 in the Supporting Information). Interestingly, for complex **3 a**, with increasing temperature from 273 to 323 K, the emission intensity of the dimer became increasingly stronger, which reminded us of the characteristics of TADF. However, as the temperature continued to rise, this emission band gradually weakened, in comparison with the fluorescence of ^1^Cz. Combined with the temperature‐dependent UV/Vis spectra (Figure 3 d), the reason should be attributed to, with an increase of temperature, the thermal motions of the molecules strengthening, leading to the destruction of dimer molecules. The thermally controlled luminescent characteristics of complex **3 a** showed sensitivity upon increasing the temperature (Figure 4 e).

**Figure 6:**
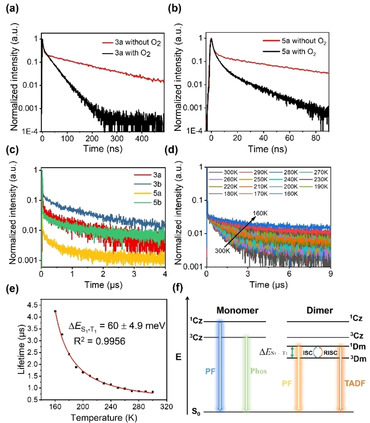
Room‐temperature PL kinetics of a) **3 a** and b) **5 a** in THF under Ar and O_2_ saturation; [**3 a**]=[**5 a**]=1×10^−3^ m; *λ*_ex_=377.4 nm. c) Transient PL decay curves of **3 a**, **3 b**, **5 a**, and **5 b** in the nondoped solid state; *λ*_ex_=377.4 nm. d) Temperature‐dependent PL kinetics of **3 a** at 200–300 K in the nondoped solid state; *λ*_ex_=377.4 nm. e) The temperature‐dependent PL decay plots of **3 a** fitted to Equation (1). The value of Δ*E* ${}_{S{}_{1}-T{}_{1}}$ was derived from least‐squares curve fitting. f) Simplified energy diagrams and kinetics schemes of the monomer and dimer. ISC: intersystem crossing. PF: prompt fluorescence; Phos: phosphorescence; Δ*E* ${}_{S{}_{1}-T{}_{1}}$ : singlet–triplet splitting energy.

The transient PL decay for the emission was investigated in both solution and nondoped solid state (Table 2 and Figure S27 in the Supporting Information). All of the complexes in THF under an inert atmosphere show short lifetimes at around 450 nm for about 2.8–4.0 ns, which corresponds to the emission originating from ^1^Cz. Meanwhile, it is interesting to note that the emission at longer wavelength exhibits a biexponential decay with a shorter component of 4.1–6.7 ns and a longer component of 28.8–182.5 ns. Combined with temperature‐dependent PL experiments, the emission band at around 600 nm might be attributed to the prompt fluorescence and TADF of the dimer, which probably originates from the separated HOMO and LUMO distributions in the dimer molecule. To investigate the effect of the triplet on the delayed component, excited‐state lifetimes of **3 a** and **5 a** were measured in the presence of triplet‐quenching oxygen (Figure 6 a and b).[^3e^, ^12^] If oxygen was bubbled through the solution for 3 min, for **3 a**, the delayed fluorescence lifetime was decreased from 182.5 to 35.4 ns, and, for **5 a**, the delayed component dropped to 18.4 ns, which indicated that the triplet had a crucial role in the delayed‐component emission because of the efficient RISC from T_1_ to S_1_.

Longer delayed‐component lifetimes were observed in the nondoped solid state in the microsecond range (0.6–2.4 μs; Figure 6 c). To examine the small Δ*E* ${}_{S{}_{1}-T{}_{1}}$ of TADF, temperature‐dependent PL kinetics of **3 a** were recorded from 300 to 160 K (Figure 6 d), which presented a temperature dependence of increasing delayed‐component lifetimes with decreasing temperature. Because the PLQYs of these complexes are limited at around 50 %, we attempted to use a two‐level model to evaluate Δ*E* ${}_{S{}_{1}-T{}_{1}}$ through Equation 1,^[6c]^ in which *τ*_TADF_ is the lifetime of TADF; Δ*E* ${}_{S{}_{1}-T{}_{1}}$ is the singlet–triplet splitting energy; *T* is temperature; *k*_B_ is the Boltzmann constant; and *k* ${}_{S{}_{1}}$ and *k* ${}_{T{}_{1}}$ are the prompt fluorescence and phosphorescent rate constants, respectively.

(1) $$\tau_{TADF}=\frac{3+\exp\left(\frac{\Delta E_{S1-T1}}{k_{B}T}\right)}{3k_{T1}+k_{S1}\exp\left(\frac{\Delta E_{S1-T1}}{k_{B}T}\right)}$$

As shown in Figure 6 e, these plots were fitted to afford Δ*E* ${}_{S{}_{1}-T{}_{1}}$ =60 meV, which indicated that the triplet energy level was quite close to the singlet energy level. Meanwhile, the TADF process could also be confirmed by Figure 4 d and e.

It is necessary to compare the excited‐state energies of the dimer (Dm) and localized Cz. To improve the reverse ISC efficiency, the ^1, 3^Dm energies must be kept lower than that of ^3^Cz. The emission spectra of **3 a**, **3 b**, **5 a**, and **5 b** in 2‐MeTHF were measured at 300 and 80 K (Figure S28 in the Supporting Information). The emission bands of dimers were located at 600 nm at room temperature. However, the spectral profiles underwent a huge change in frozen solvent if the temperature dropped to 80 K. At this point, there was a strong emission band at around 500 nm, which displayed vibronic fine structure. The spectral shifts were the result of destabilization of intermolecular CT due to the rigid environment in the freezing solution.[^6c^, ^6d^, ^6e^] Therefore, the proportion of ^3^Cz/Dm gradually increased with decreasing temperature: once the temperature reached to 80 K, ^3^Cz emission was observed because the energies of the ^1, 3^Dm states were above the ^3^Cz state. The vibronic fine structure at 500 nm, along with the long lifetime at 80 K (=0.7 ms), was in agreement with the locally excited ^3^Cz state of KCz (Figures S29 and S30 in the Supporting Information). These results demonstrate that the ^1, 3^Dm levels are below that of ^3^Cz at room temperature. Based on the above experimental results, the singlet and triplet distributions of the monomer and dimer are illustrated in Figure 6 f.

### Single‐component multicolor emission studies

**Figure 7:**
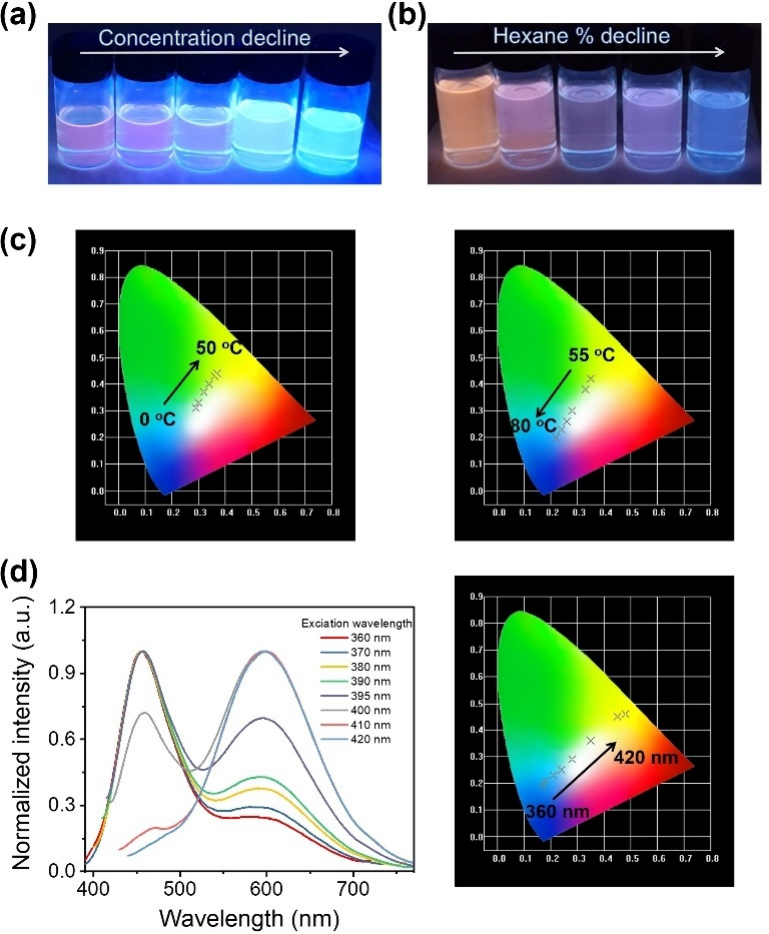
Room‐temperature fluorescence photographs of **3 a**: a) at different concentrations and b) with different volume fractions of hexane under UV irradiation. c) Several luminescent color coordinates for **3 a** plotted in a CIE 1931 chromaticity diagram, corresponding to PL emission at different temperatures (0–80 °C); [**3 a**]=1.0×10^−3^ m; *λ*_ex_=380 nm. d) Left: Room‐temperature PL spectra of **3 b** in THF measured at various excitation wavelengths. Right: Several luminescent color coordinates for **3 b** plotted in a CIE 1931 chromaticity diagram, corresponding to PL emission at different excitation wavelengths (360–420 nm); [**3 b**]=1.0×10^−3^ m.

After confirming the origin of the dual emissions, it was found that the contributions from the monomer and dimer emissions could result in single‐component multicolor emission. The position and intensity of dual‐emission bands of these complexes could be modulated by the concentration, degree of aggregation, and temperature based on the above experiments (Figure 4 b–d and Figures S13, S14, and S26 in the Supporting Information). The fluorescence photographs of **3 a** at different concentrations and with different volume fractions of hexane are shown in Figure 7 a and b, respectively. Owing to the TADF nature and switching between monomer and dimer, the color change is sensitive to temperature. As shown in Figure 7 c, as the temperature rose, the color of **3 a** in the 1×10^−3^ m solution in THF under 380 nm excitation changed from white to yellow to white to blue, which is promising for a temperature sensor.

In addition, modulation of the dual emissions is also investigated by changing the excitation wavelengths (Figure 7 d and Figure S31 in the Supporting Information). Taking **3 b** as an example, as the excitation wavelengths varied from 360 to 420 nm, the dimer emission bands gradually enhanced and the emission of ^1^Cz weakened. So, the overall emission color ranged from blue to white to yellow, and the corresponding CIE^[14]^ coordinates are displayed in Figure 7 d.

## Conclusion

We have designed and synthesized a series of two‐coordinate Au^I^ or Cu^I^ complexes (**3 a**, **3 b**, **5 a**, and **5 b**) based on a simple molecular model of PyTz–metal–Cz. The complexes could self‐assemble into a head‐to‐tail configuration through intermolecular π–π interactions that facilitated intermolecular CT between Cz and PyTz, taking advantage of the low‐energy LUMO of the carbene ligand. PL kinetics demonstrated that these molecules exhibited a delayed component in both solution and the solid state in the absence of triplet‐quenching oxygen, which was ascribed to TADF emitters. In addition, these complexes exhibited longer delayed‐component lifetime (0.6–2.4 μs) and enhanced QYs in the solid state. Temperature‐dependent studies of **3 a** revealed a rather small energy separation between S_1_ and T_1_ (Δ*E* ${}_{S{}_{1}-T{}_{1}}$ =60 meV).

Efficient absorption and emission spectra demonstrated the existence of the monomer–dimer equilibrium in solution, presenting dual‐emission bands at around 450 nm and 600 nm, respectively. The emission band in the low‐energy region is solvatochromic as the polarity of the solution changes. Furthermore, there exists a locally excited triplet state, ^3^Cz, that dominates emission in frozen solvent because of the destabilization of the intermolecular CT states. We also demonstrate that, through combining the dual emissions generated by the monomer and dimer, the series of complexes displayed single‐component multicolor emission. The color tunability could be realized by changing the excitation wavelength, temperature, degree of aggregation, and concentration of the complex. Further studies to explore TADF materials and the corresponding application of this dimeric mode are in progress.

## Experimental Section

Experimental details, including materials, experimental procedures, chemical preparation of the compounds, photophysical characterization, X‐ray crystallographic studies, calculations of the dimerization constant *K*, computational details, and NMR spectroscopy data for the compounds, are provided in the Supporting Information.

## Conflict of interest

The authors declare no conflict of interest.

## Supporting information

As a service to our authors and readers, this journal provides supporting information supplied by the authors. Such materials are peer reviewed and may be re‐organized for online delivery, but are not copy‐edited or typeset. Technical support issues arising from supporting information (other than missing files) should be addressed to the authors.

Supplementary
