# Supplementary material for: Thermally Activated Delayed Fluorescence from d10‐Metal Carbene Complexes through Intermolecular Charge Transfer and Multicolor Emission with a Monomer–Dimer Equilibrium
Source: Chemistry. 2020 Nov 18;26(71):17222–9. doi: 10.1002/chem.202004106 (PMC7839463; doi:10.1002/chem.202004106)
Supplement: Supplementary file 1 — Supplementary [file CHEM-26-17222-s001.pdf]

Chemistry–A European Journal

Supporting Information

Thermally Activated Delayed Fluorescence from d^{10}-Metal Carbene Complexes through Intermolecular Charge Transfer and Multicolor Emission with a Monomer–Dimer Equilibrium

Lei Cao,[a] Shiqing Huang,[a] Wei Liu,[a] Hongyan Zhao,[a] Xiao-Gen Xiong,[b] Jian-Ping Zhang,[a] Li-Min Fu,*[a] and Xiaoyu Yan*[a]

Table of Contents

Table of Contents S1

Experimental Procedures S2

General information S2

Photophysical characterization S2

X-ray crystallographic studies S3

Results and Discussion S4

Experimental details and characterization data S4

Crystallographic data S8

Calculations of the dimerization constant *K* using absorption spectra S16

Monomer-Dimer equilibrium studies S17

Computational details S25

Thermally activated delayed fluorescence (TADF) studies S48

Multicolor emission construction S53

Electrochemical Characterization S55

NMR spectra characterization S56

References S65

Experimental Procedures

General information

All manipulations of air and moisture sensitive compounds were carried out under an inert atmosphere of dry argon in a glovebox or using standard Schlenk techniques under an atmosphere of dry argon or nitrogen in flame-dried glassware. All chemical reagents and deuterated solvent were purchased from Alfa, Acros, Aldrich, TCI, and J&K and used without further purification. Diethyl ether, dichloromethane (DCM), hexane and tetrahydrofuran (THF) were dried using an inert solvent purification system. ^{1}H NMR spectra were recorded on Bruker 400 or 600 MHz spectrometer and the chemical shifts were reported in parts per million (δ) relative to internal standard TMS (0 ppm) for d_6-DMSO. The peak patterns are indicated as follows: s, singlet. d, doublet. t, triplet. m, multiplet. dd, doublet doublet. td, triplet doublet. The coupling constants, J, are reported in Hertz (Hz). ^{13}C NMR spectra were obtained at Bruker 100 or 150 MHz and referenced to the internal solvent signals (central peak is 39.96 ppm in d_6-DMSO). $CDCl_3$, DMSO and $(CD_3)_2CO$ were used as the NMR solvent. High-resolution mass spectra (HR-MS) were acquired on Thermo (Q-Exactive) instrument using electrospray ionization mode (ESI).

Photophysical characterization

Absorption spectra were measured on Shimadzu UV–3600 UV–VIS–NIR spectrophotometer and steady state emission spectra were recorded on HITACHI F–4600 fluorescence spectrophotometer. Temperature-dependent absorption measurements in the range of 273–348 K were performed using a TCC–100 Thermoelectrically Temperature Controlled Cell Holder. Transient PL decays were recorded using Edinburgh fluorescence spectrometer (FLS–980) equipped with a 377.4 nm laser excitation source and temperature-dependent transient PL characterization in the range of 160–300 K were performed using Oxford-instruments OptistatDN2. Long-lived phosphorescence decays of ^{3}Cz were measured using a Division of Edinburgh instruments equipped with a μF2 lamp. Quantum yields at room temperature were measured by an integrating sphere (SM4, Edinburgh Instrument, UK). The

solutions of all the complexes were prepared to the quartz cuvettes under Ar in the glovebox to avoid contact with air. The non-doped solid means the microcrystalline sample.

X-ray crystallographic studies

The single crystals of **1, 2, 3a, 4** and **5a–b** suitable for X-ray analysis were obtained by the methods in the 'Experimental Details'. Data collections for them were performed on a 'Bruker APEX-II CCD' diffractometer, using graphite-monochromated Mo Kα radiation (λ = 0.71073 Å). Using Olex2[1], the structures were solved with the ShelXT[2] structure solution program using Intrinsic Phasing and refined with the ShelXL[3] refinement package using Least Squares minimization. Refinement was performed on F^2 anisotropically for all the non-hydrogen atoms by the full-matrix least-squares method. The hydrogen atoms were placed at the calculated positions and were included in the structure calculation without further refinement of the parameters.

Crystallographic data have been deposited with the Cambridge Crystallographic Data Centre as supplementary publication nos. CCDC Number: **1**, 1915181. **2**, 1915182. **3a**, 1915183. **4**, 1915184. **5a**, 1915185. **5b**, 1915186. These data can be obtained free of charge from The Cambridge Crystallographic Data Centre via www.ccdc.cam.ac.uk/data_request/cif.

Refinement details for 1: There exists a disordered iodine anion in **1** due to thermodynamic vibration. Iodine anion in **1** is disordered over two sites with a occupancy ratio = 0.98:0.02.

Refinement details for 2: The complex **2** presents one disordered Au atom due to the heavy atom effect of Au. Au atom is disordered over two sites with a occupancy ration = 0.99:0.01. The commands DELU, SIMU and ISOR were applied to mostly restrain the disorder of Au atom. The command DIFX was applied to constrain the bond length of carbene carbon and a disordered Au in **2**. In addition, the command OMIT was applied to remove the bad reflections with large errors, which would seriously affect the refinement progress.

Refinement details for 5b: The commands DELU and SIMU were applied to restrain the thermodynamic vibration of tertiary butyl. And the command DIFX was applied to fix the relative position of methyl in tertiary butyl.

Results and Discussion

Experimental details and characterization data

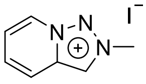

1

[1,2,3]-triazolo-[1,5-*a*]-pyridine[4] (536 mg, 1.00 mmol) and iodomethane (146 mg, 1.30 mmol) were combined in a Schlenk flask and dissolved with acetonitrile (30 mL). The resulting suspension was stirred at 70 °C for 2 days. All volatiles were evaporated under vacuum and the solid residue was treated with DCM. The insoluble residue was filtered off and the combined filtrates were evaporated. The residue was washed with hexane to give the target product as a yellow solid in 90% yield (235 mg, 0.9 mmol). X-ray-quality crystals were obtained by slow diffusion of ether into DCM solution of the corresponding complex. ^{1}H NMR (400 MHz, DMSO-d_6) δ 9.41 (dd, *J* = 7.6, 1.1 Hz, 1H), 9.36 (s, 1H), 8.41 (dd, *J* = 9.0, 1.3 Hz, 1H), 7.91 (td, *J* = 7.0, 1.0 Hz, 1H), 7.82 (td, *J* = 7.0, 1.4 Hz, 1H), 4.54 (s, 3H). ^{13}C NMR (101 MHz, Acetone-d_6) δ 135.9, 130.5, 126.1, 124.9, 122.8, 120.1, 40.5. HR-MS (ESI) calcd for $C_7H_8N_3$ ([M–I]$^+$): m/z 134.0713, Found: m/z 134.0716.

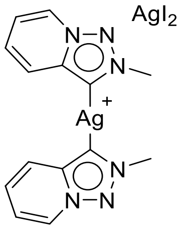

6

A mixture of **1** (2.610 g, 10 mmol) and Ag_2O (6.952 g, 30 mmol) in DCM (70 mL) was placed in a Schlenk tube at room temperature for 3 days without light inside the glovebox. The solution was concentrated after filtration. The solid residue was washed with hexane to give the target product as a brown solid in 85% yield (6.263 g, 8.5 mmol). X-ray-quality crystals were obtained by slow diffusion of ether into DCM solution of the corresponding complex. ^{1}H NMR (400 MHz, Chloroform-*d*) δ 8.62 (d, *J* = 6.8 Hz, 2H), 8.16 (d, *J* = 8.7 Hz, 2H), 7.44 (td, *J* = 6.9, 1.2 Hz, 2H), 7.39 (td, *J* = 6.9, 1.5 Hz, 2H), 4.48 (s, 6H). ^{13}C NMR (150 MHz, Chloroform-*d*) δ 163.8, 143.5, 125.6, 124.6, 123.9, 121.1, 43.7. HR-MS (ESI) calcd for $C_{14}H_{14}AgN_6$ ([M–AgI_2]$^+$): m/z 373.0325, Found: m/z 373.0316.

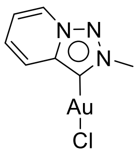

2

A mixture of **6** (737 mg, 1 mmol) and (THT)AuCl[5] (800 mg, 2.5 mmol) in DCM (25 mL) was placed in a Schlenk tube at room temperature for 2h without light inside the glovebox. The solution was concentrated after filtration. The solid residue was washed with hexane to give the target product as a yellow solid in 85% yield (311 mg, 0.85 mmol). X-ray-quality crystals were obtained by slow diffusion of ether into a dilute DCM solution of the complex. ^{1}H NMR (400 MHz, Chloroform-*d*) δ 8.58 (d, *J* = 7.0 Hz, 1H), 8.14 (d, *J* = 8.9 Hz, 1H), 7.48 – 7.44 (m, 1H), 7.41 – 7.38 (m, 1H), 4.41 (s, 3H). ^{13}C NMR (100 MHz, Chloroform-*d*) 142.1, 131.8, 125.6, 124.0, 123.9, 121.4, 42.7. HR-MS (ESI) calcd for $C_{14}H_{14}AuN_6$ ($[2M–AuCl_2]^+$): m/z 463.0940, Found: m/z 463.0935.

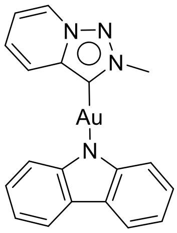

3a

Potassium *t*-butoxide (112 mg, 1 mmol) and carbazole (167 mg, 1 mmol) were combined in a Schlenk flask and dissolved with THF (30 mL) at room temperature. After 30 minutes, **2** (366 mg, 1 mmol) was added to the the mixture and then it was stirred at room temperature for 3h. All volatiles were evaporated under vacuum and the solid residue was treated with DCM. The insoluble residue was filtered off and the combined filtrates were evaporated. The residue was washed with hexane to give the target product as a white solid in 92% yield (457 mg, 0.92 mmol). X-ray-quality crystals were obtained by slow diffusion of ether into DCM solution of the corresponding complex. ^{1}H NMR (400 MHz, Chloroform-*d*) δ 8.56 (d, *J* = 6.9 Hz, 1H), 8.23 (d, *J* = 8.8 Hz, 1H), 8.11 (d, *J* = 7.5 Hz, 2H), 7.81 (d, *J* = 8.1 Hz, 2H), 7.46-7.42 (m, 1H), 7.41 – 7.34 (m, 1H), 7.36 – 7.32 (m, 2H), 7.08 – 7.04 (m, 2H), 4.50 (s, 3H). ^{13}C NMR (100 MHz, Chloroform-*d*) δ 156.6, 149.7, 139.5, 129.7, 125.8, 123.4, 120.3, 119.7, 119.4, 116.0,

113.7, 110.6, 29.7. HR-MS (ESI) calcd for $C_{19}H_{16}AuN_4$ ($[M+H]^+$): m/z 497.1035, Found: m/z 497.1031.

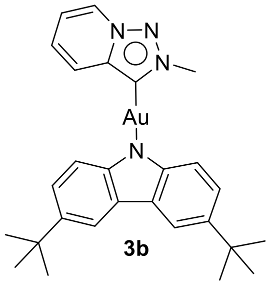

3b

Potassium *t*-butoxide (112 mg, 1 mmol) and 3, 6-Di-tert-butylcarbazole (279 mg, 1 mmol) were combined in a Schlenk flask and dissolved with THF (30 mL). The resulting suspension was stirred at room temperature. After 30 minutes, **2** (366 mg, 1 mmol) was added to the the mixture and then it was stirred at room temperature for 3h. All volatiles were evaporated under vacuum and the solid residue was treated with DCM. The insoluble residue was filtered off and the combined filtrates were evaporated. The residue was washed with hexane to give the target product as a white solid in 94% yield (572 mg, 0.94 mmol). X-ray-quality crystals have were obtained by slow diffusion of ether into DCM solution of the corresponding complex. ^{1}H NMR (600 MHz, Chloroform-*d*) δ 8.46 (d, *J* = 6.84 Hz, 1H), 8.14 (d, *J* = 8.6 Hz, 1H), 8.07 (s, 2H), 7.69 (dd, *J* = 8.5, 0.6 Hz, 2H), 7.37 (dd, *J* = 8.4, 2.0 Hz, 2H), 7.33 (td, *J* = 6.9, 1.2 Hz, 1H), 7.29 (td, *J* = 6.9, 1.6 Hz, 1H), 4.43 (s, 3H), 1.47 (s, 18H). ^{13}C NMR (100 MHz, Chloroform-*d*) δ 159.7, 148.2, 138.5, 129.8, 124.0, 123.7, 123.5, 121.4, 116.2, 115.3, 112.9, 110.0, 34.6, 32.3, 29.7. HR-MS (ESI) calcd for $C_{27}H_{32}AuN_4$ ($[M+H]^+$): m/z 609.2287, Found: m/z 609.2288.

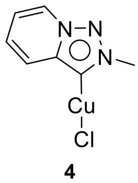

4

A mixture of **6** (737 mg, 1 mmol) and CuCl (99 mg, 1 mmol) in DCM (25 mL) was placed in a Schlenk tube at room temperature for 2h without light inside the glovebox. The solution was concentrated after filtration. The solid residue was washed with hexane to give the target product as a yellow solid in 88% yield (204 mg, 0.88 mmol). X-ray-quality crystals were obtained by slow diffusion of ether into a dilute DCM solution of the complex. ^{1}H NMR (400

MHz, Chloroform-*d*) δ 8.58 (d, *J* = 6.8 Hz, 1H,), 8.13 (d, *J* = 8.7 Hz, 1H), 7.43-7.34 (m, 2H), 4.46 (s, 3H). ^{13}C NMR (100 MHz, Chloroform-*d*) δ 143.5, 129.9, 125.1, 124.3, 123.8, 121.1, 43.2. HR-MS (ESI) calcd for $C_{14}H_{14}CuN_6$ ([2M–$CuCl_2$]$^+$): m/z 329.0570, Found: m/z 329.0567.

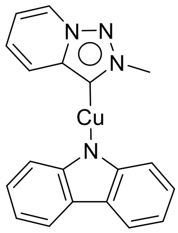

5a

Potassium *t*-butoxide (112 mg, 1 mmol) and carbazole (167 mg, 1 mmol) were combined in a Schlenk flask and dissolved with THF (30 mL). The resulting suspension was stirred at room temperature. After 30 minutes, **4** (232 mg, 1 mmol) was added to the mixture and then it was stirred at room temperature for 3h. All volatiles were evaporated under vacuum and the solid residue was treated with DCM. The insoluble residue was filtered off and the combined filtrates were evaporated. The residue was washed with hexane to give the target product as a faint yellow solid in 88% yield (319 mg, 0.88 mmol). X-ray-quality crystals were obtained by slow diffusion of ether into DCM solution of the corresponding complex. ^{1}H NMR (400 MHz, Chloroform-*d*) δ 8.61 (d, *J* = 6.9 Hz, 1H), 8.23 (d, *J* = 8.8 Hz, 1H), 8.11 (d, *J* = 7.7 Hz, 2H), 7.67 (d, *J* = 8.1 Hz, 2H), 7.44 – 7.41 (m, 1H), 7.40 – 7.37 (m, 1H), 7.31 (t, *J* = 7.5 Hz, 2H), 7.05 (t, *J* = 7.3 Hz, 2H) 4.56 (s, 3H). ^{13}C NMR (100 MHz, Methylene Chloride-*d*$_2$) 150.3, 139.5, 129.8, 125.8, 125.2, 124.1, 123.1, 121.0, 120.1, 119.3, 115.4, 110.6, 29.7. HR-MS (ESI) calcd for $C_{19}H_{16}CuN_4$ ([M+H]$^+$): m/z 363.0665, Found: m/z 363.0662.

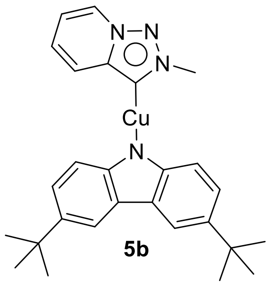

5b

Potassium *t*-butoxide (112 mg,1 mmol) and 3, 6-Di-tert-butylcarbazole (279 mg, 1 mmol) were combined in a Schlenk flask and dissolved with THF (30 mL). The resulting suspension was stirred at room temperature. After 30 minutes, **4** (232 mg, 1 mmol) was added to the mixture and then it was stirred at room temperature for 3h. All volatiles were evaporated under

vacuum and the solid residue was treated with DCM. The insoluble residue was filtered off and the combined filtrates were evaporated. The residue was washed with hexane to give the target product as a faint yellow solid in 93% yield (442 mg, 0.93 mmol). X-ray-quality crystals were obtained by slow diffusion of ether into DCM solution of the corresponding complex. ^{1}H NMR (400 MHz, Chloroform-*d*) δ 8.60 (d, *J* = 6.8 Hz, 1H), 8.18 (d, *J* = 8.3 Hz, 1H), 8.07 (s, 2H), 7.57 (d, *J* = 8.4 Hz, 1H), 7.47 – 7.31 (m, 5H), 4.52 (s, 3H), 1.44 (s, 18H). ^{13}C NMR (100 MHz, Methylene Chloride-d_2) 161.1, 142.2, 138.1, 129.8, 125.1, 124.1, 123.9, 123.5, 121.3, 121.0, 116.1, 110.0, 37.1, 31.8, 29.4. HR-MS (ESI) calcd for $C_{27}H_{32}CuN_4$ ($[M+H]^+$): m/z 475.1917, Found: m/z 475.1962.

Crystallographic data

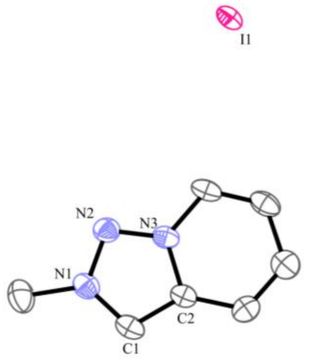

Figure S1. Oak Ridge thermal ellipsoid plot (ORTEP) representation of **1**. Thermal ellipsoids shown at 50% probability. Hydrogen atoms are removed for clarity.

Table S1. Crystal data and structure refinement for **1**.

Empirical formula	$C_7H_8IN_3$
Formula weight	261.06
Temperature/K	199.97
Crystal system	orthorhombic
Space group	Pbca
a/Å	7.3685(4)
b/Å	11.9359(6)
c/Å	20.5150(8)
α/°	90
β/°	90
γ/°	90
Volume/Å3	1804.29(15)

Z	8
ρ_{calc}g/cm^3	1.922
μ/mm^{-1}	3.491
F(000)	992.0
Crystal size/mm^3	0.17 × 0.15 × 0.12
Radiation	MoKα (λ = 0.71073)
2θ range for data collection/°	6.796 to 55.006
Index ranges	-9 ≤ h ≤ 8, -15 ≤ k ≤ 15, -26 ≤ l ≤ 24
Reflections collected	11521
Independent reflections	2063 [R_{int} = 0.1326, R_{sigma} = 0.0633]
Data/restraints/parameters	2063/0/111
Goodness-of-fit on F^2	1.068
Final R indexes [I>=2σ (I)]	R_1 = 0.0409, wR_2 = 0.1003
Final R indexes [all data]	R_1 = 0.0502, wR_2 = 0.1066
Largest diff. peak/hole / e Å^{-3}	1.70/-1.25

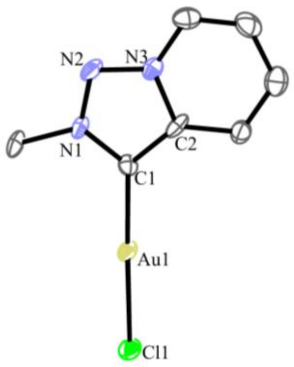

Figure S2. Oak Ridge thermal ellipsoid plot (ORTEP) representation of **2** Thermal ellipsoids shown at 50% probability. Hydrogen atoms are removed for clarity.

Table S2. Crystal data and structure refinement for **2**.

Empirical formula	$C_7H_7ClAuN_3$
Formula weight	365.57
Temperature/K	170.0
Crystal system	monoclinic
Space group	$P2_1/c$

a/Å	8.4013(7)
b/Å	13.7741(10)
c/Å	8.0374(6)
α/°	90
β/°	107.491(2)
γ/°	90
Volume/Å^3	887.09(12)
Z	4
ρ_{calc}g/cm^3	2.737
μ/mm^{-1}	16.829
F(000)	664.0
Crystal size/mm^3	0.3 × 0.11 × 0.11
Radiation	MoKα (λ = 0.71073)
2θ range for data collection/°	5.916 to 49.982
Index ranges	-9 ≤ h ≤ 9, -16 ≤ k ≤ 16, -9 ≤ l ≤ 9
Reflections collected	11903
Independent reflections	1497 [R_{int} = 0.1725, R_{sigma} = 0.0649]
Data/restraints/parameters	1497/10/120
Goodness-of-fit on F^2	1.055
Final R indexes [I>=2σ (I)]	R_1 = 0.0350, wR_2 = 0.0839
Final R indexes [all data]	R_1 = 0.0365, wR_2 = 0.0859
Largest diff. peak/hole / e Å^{-3}	2.01/-1.54

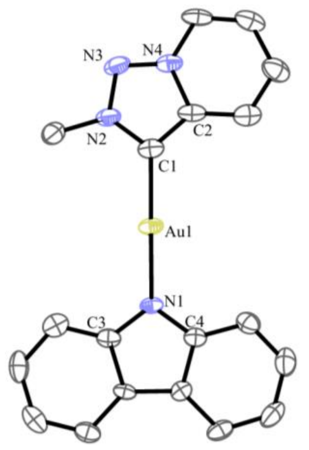

Figure S3. Oak Ridge thermal ellipsoid plot (ORTEP) representation of **3a**. Thermal ellipsoids shown at 50% probability. Hydrogen atoms are removed for clarity.

Table S3. Crystal data and structure refinement for **3a**.

Empirical formula	$C_{19}H_{15}AuN_4$
Formula weight	496.32
Temperature/K	150.0
Crystal system	monoclinic
Space group	$P2_1/n$
a/Å	8.8048(7)
b/Å	8.3497(6)
c/Å	21.7180(17)
α/°	90
β/°	93.399(3)
γ/°	90
Volume/Å^3	1593.8(2)
Z	4
ρ_{calc}g/cm^3	2.068
μ/mm^{-1}	9.237
F(000)	944.0
Crystal size/mm^3	0.18 × 0.15 × 0.12
Radiation	MoKα (λ = 0.71073)
2θ range for data collection/°	5.104 to 54.976
Index ranges	-11 ≤ h ≤ 11, -10 ≤ k ≤ 10, -28 ≤ l ≤ 26
Reflections collected	15826

Independent reflections	3623 [R_{int} = 0.1183, R_{sigma} = 0.0751]
Data/restraints/parameters	3623/0/218
Goodness-of-fit on F^2	1.053
Final R indexes [I>=2σ (I)]	R_1 = 0.0372, wR_2 = 0.0743
Final R indexes [all data]	R_1 = 0.0485, wR_2 = 0.0792
Largest diff. peak/hole / e $Å^{-3}$	2.23/-1.46

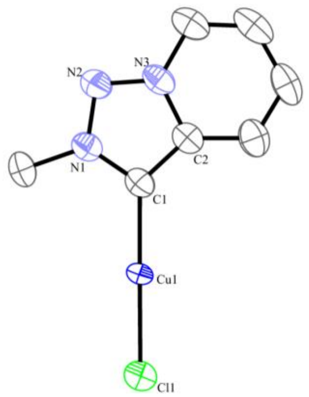

Figure S4. Oak Ridge thermal ellipsoid plot (ORTEP) representation of **4**. Thermal ellipsoids shown at 50% probability. Hydrogen atoms are removed for clarity.

Table S4. Crystal data and structure refinement for **4**.

Empirical formula	$C_7H_7ClCuN_3$
Formula weight	232.15
Temperature/K	200.01
Crystal system	monoclinic
Space group	$P2_1/c$
a/Å	9.6384(18)
b/Å	14.222(3)
c/Å	6.3585(14)
α/°	90
β/°	94.699(7)
γ/°	90
Volume/$Å^3$	868.7(3)
Z	4
$\rho_{calc}g/cm^3$	1.775

μ/mm^{-1}	2.764
F(000)	464.0
Crystal size/mm^3	0.19 × 0.15 × 0.12
Radiation	MoKα (λ = 0.71073)
2θ range for data collection/°	5.118 to 55.058
Index ranges	-11 ≤ h ≤ 12, -18 ≤ k ≤ 18, -8 ≤ l ≤ 8
Reflections collected	7203
Independent reflections	1995 [R_{int} = 0.1391, R_{sigma} = 0.1053]
Data/restraints/parameters	1995/0/110
Goodness-of-fit on F^2	1.036
Final R indexes [I>=2σ (I)]	R_1 = 0.0638, wR_2 = 0.1391
Final R indexes [all data]	R_1 = 0.1221, wR_2 = 0.1642
Largest diff. peak/hole / e Å^{-3}	0.81/-0.60

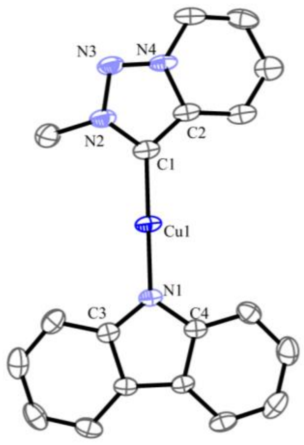

Figure S5. Oak Ridge thermal ellipsoid plot (ORTEP) representation of **5a**. Thermal ellipsoids shown at 50% probability. Hydrogen atoms are removed for clarity.

Table S5. Crystal data and structure refinement for **5a**.

Empirical formula	$C_{19}H_{15}CuN_4$
Formula weight	362.89
Temperature/K	170.0
Crystal system	monoclinic
Space group	$P2_1/n$

a/Å	8.7660(8)
b/Å	8.1711(7)
c/Å	21.940(2)
α/°	90
β/°	94.323(3)
γ/°	90
Volume/Å^3	1567.0(2)
Z	4
ρ_{calc}g/cm^3	1.538
μ/mm^{-1}	1.400
F(000)	744.0
Crystal size/mm^3	0.26 × 0.09 × 0.08
Radiation	MoKα (λ = 0.71073)
2θ range for data collection/°	5.148 to 55.038
Index ranges	-11 ≤ h ≤ 11, -10 ≤ k ≤ 10, -26 ≤ l ≤ 28
Reflections collected	21232
Independent reflections	3608 [R_{int} = 0.0472, R_{sigma} = 0.0357]
Data/restraints/parameters	3608/0/218
Goodness-of-fit on F^2	1.039
Final R indexes [I>=2σ (I)]	R_1 = 0.0373, wR_2 = 0.0774
Final R indexes [all data]	R_1 = 0.0582, wR_2 = 0.0852
Largest diff. peak/hole / e Å^{-3}	0.35/-0.42

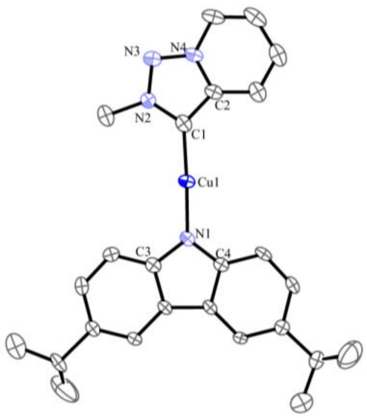

Figure S6. Oak Ridge thermal ellipsoid plot (ORTEP) representation of **5b**. Thermal ellipsoids shown at 50% probability. Hydrogen atoms are removed for clarity.

Table S6. Crystal data and structure refinement for **5b**.

Empirical formula	$C_{27}H_{31}CuN_4$
Formula weight	475.10
Temperature/K	199.99
Crystal system	monoclinic
Space group	C2/m
a/Å	22.2569(10)
b/Å	6.7435(3)
c/Å	18.2137(9)
α/°	90
β/°	117.315(2)
γ/°	90
Volume/Å^3	2428.9(2)
Z	4
ρ_{calc}g/cm^3	1.299
μ/mm^{-1}	0.920
F(000)	1000.0
Crystal size/mm^3	0.21 × 0.12 × 0.11
Radiation	MoKα (λ = 0.71073)
2θ range for data collection/°	5.034 to 49.99
Index ranges	-26 ≤ h ≤ 26, -8 ≤ k ≤ 8, -21 ≤ l ≤ 21
Reflections collected	10959
Independent reflections	2297 [R_{int} = 0.1968, R_{sigma} = 0.1000]
Data/restraints/parameters	2297/44/192
Goodness-of-fit on F^2	1.049
Final R indexes [I>=2σ (I)]	R_1 = 0.0780, wR_2 = 0.2064
Final R indexes [all data]	R_1 = 0.0879, wR_2 = 0.2181
Largest diff. peak/hole / e Å^{-3}	1.24/-1.56

Table S7. Selected bond lengths and angles in complex **3a** and **5a-5b**.

Complex	**3a**	**5a**	**5b**
C2-C1-N1-C4	4.61^{o}	5.91^{o}	0°
N2-C1-C2	102.2 (5)^{o}	101.7 (2)^{o}	100.6 (6)^{o}
C2-C1-M*	131.1 (4)^{o}	131.7 (19)^{o}	133.4 (5)^{o}
N2-C1-M*	126.5 (4)^{o}	126.3 (2)^{o}	126.1 (4)^{o}
C3-N1-C4	106.1 (4)^{o}	105.4 (18)^{o}	104.9 (5)^{o}
C3-N1-M*	126.8 (3)^{o}	127.9 (16)^{o}	126.9 (4)^{o}
C4-N1-M*	126.8 (3)^{o}	126.2 (16)^{o}	128.2 (4)^{o}
C1-M*-N1	176.8 (18)^{o}	176.8 (9)^{o}	177.7 (2)^{o}
C1-M*	1.982 (6) Å	1.877 (2) Å	1.863 (7) Å
N1-M*	2.020 (4) Å	1.8609 (19) Å	1.846 (5) Å
C1-N1	4.002 (10) Å	3.7379 (21) Å	3.709 (12) Å

* M = metal Au or Cu

Calculations of the dimerization constant *K* using absorption spectra

Determination of dimer constant K for self-aggregation is represented by (*Eq.* S1) and (*Eq.* S2),

A + A ↔ A-A

$K = [A\text{-}A]/[A]^2$ (*Eq.* S1)

$[A_0] = [A] + 2[A\text{-}A]$ (*Eq.* S2)

where [A-A], [A], and $[A_0]$ are dimer, monomer, and total concentration of the compound A, respectively. It is assumed that the observed ε_{obs} value is the weighted average of those of the monomer and dimer, and ε_{obs} is described as:

$\varepsilon_{obs} = \{[A]\varepsilon_m + 2[A\text{-}A]\varepsilon_d\}/[A_0]$ (*Eq.*S3)

where ε_{obs} is the observed molar absorption coeffcient, while ε_d and ε_m are the limiting values of dimer and monomer. Combining (*Eq.* S1) and (*Eq.* S2) gives the (*Eq.* S4).

$2K[A]^2 + [A] – [A_0] = 0$ (*Eq.* S4)

So the expression of [A] could be rewritten in (*Eq.* S5)

$[A] = \{(1 + 8K[A_0])^{1/2} – 1\}/4K$ (*Eq.* S5)

Thus, (*Eq.* S3) and (*Eq.* S5) gives

$\varepsilon_{obs} = \varepsilon_d – (\varepsilon_d – \varepsilon_m)\{(1 + 8K[A_0])^{1/2} – 1\}/4K[A_0]$ (*Eq.* S6)

The three variable parameters K, ε_d, and ε_m were determined by nonlinear least-squares fit using Origin Pro 2017.

Monomer-Dimer equilibrium studies

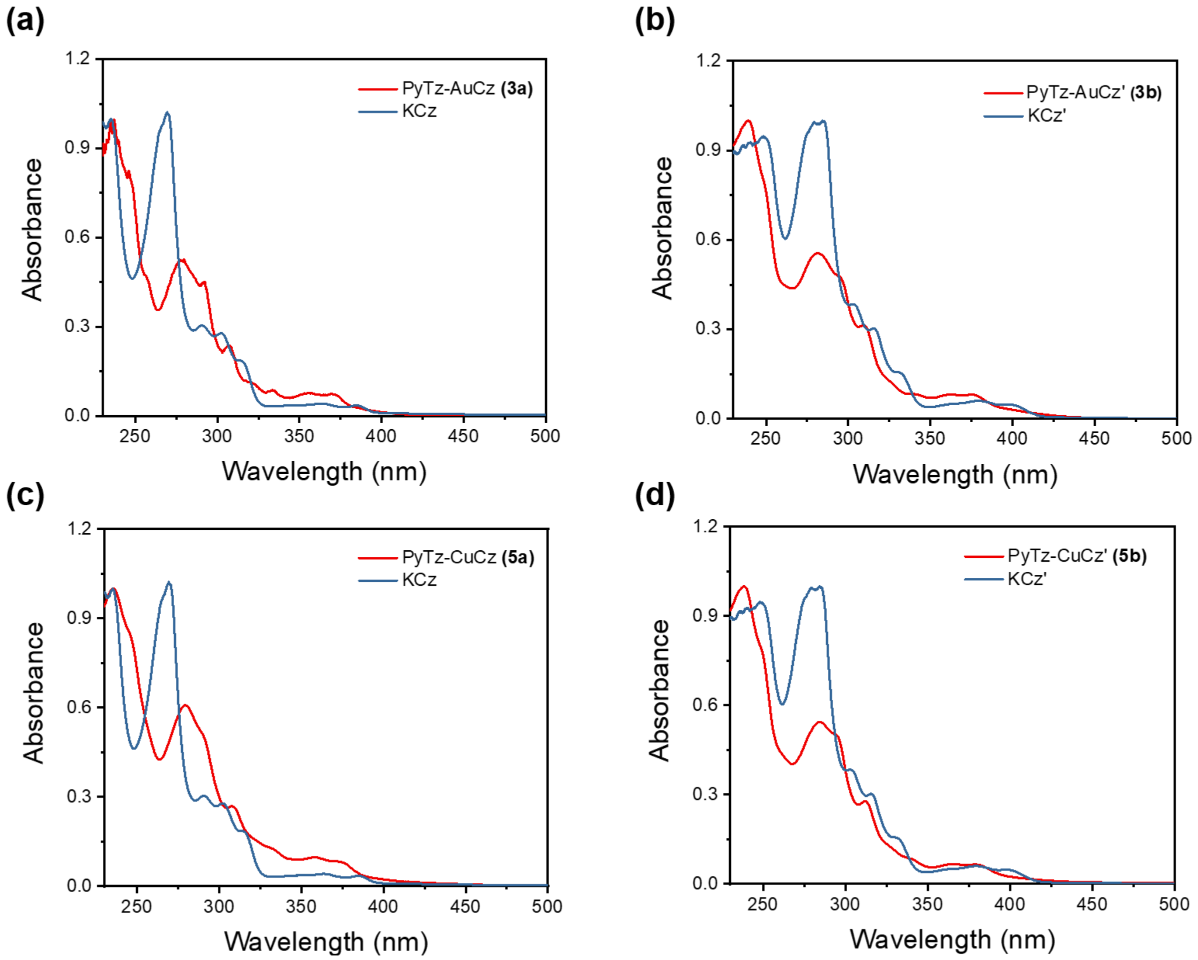

Figure S7. Absorption spectra of complexes (a) **3a** and potassium carbazolide (KCz); (b) **3b** and potassium 3,6-di-tert-butylcarbazolide (KCz'); (c) **5a** and potassium carbazolide (KCz) (d) **5b** and potassium 3,6-di-tert-butylcarbazolide (KCz') in THF.

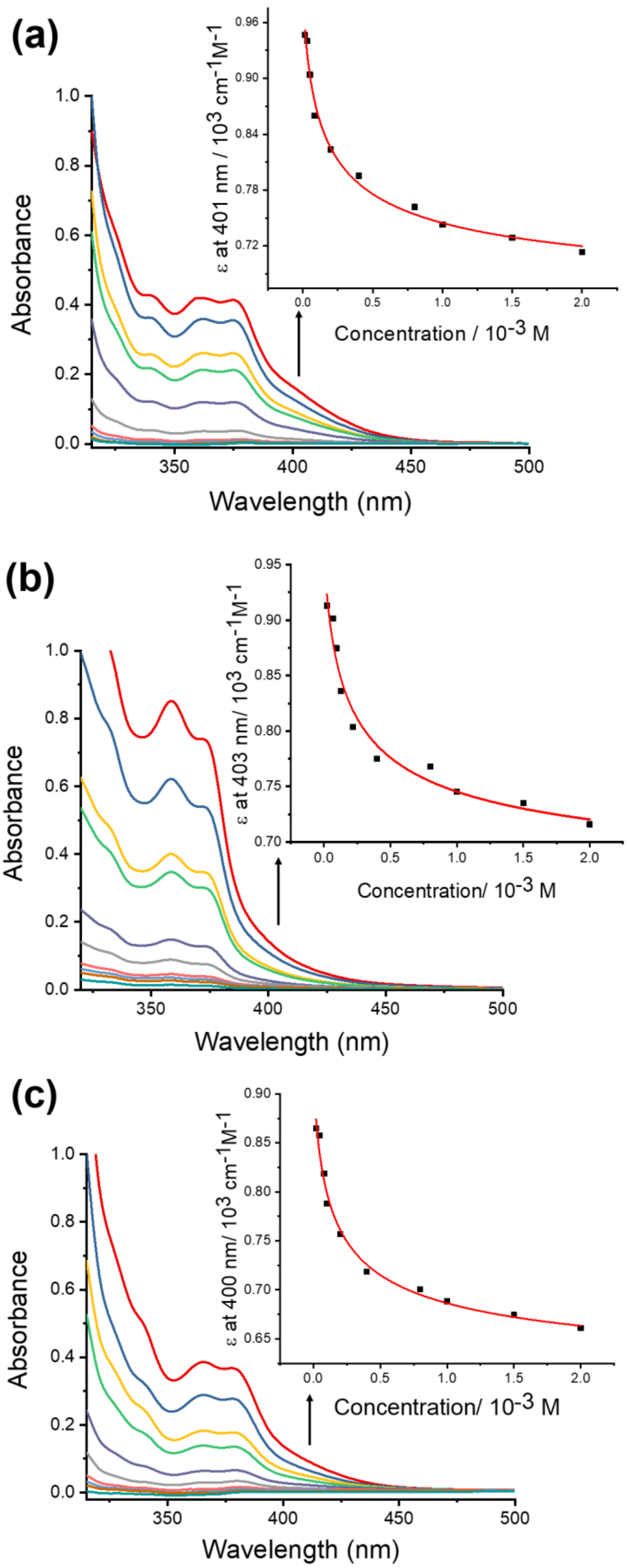

Figure S8. Absorption spectra of complexes (a) **3b**, (b) **5a** and (c) **5b** from 1 × 10^{-5} M to 2 × 10^{-3} M in THF at 298 K. Insets: The dimerization plot for monomer–dimer equilibrium monitored at the absorption wavelength of the dimer.

(a)

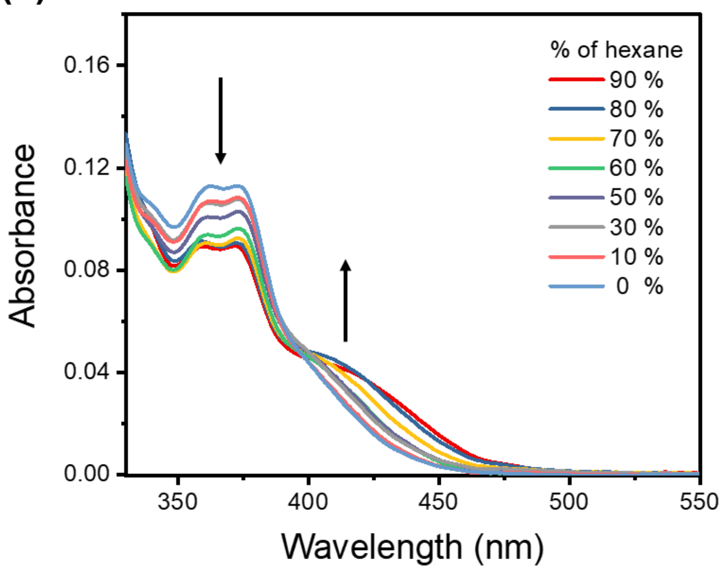
% of hexane
90 %
80 %
70 %
60 %
50 %
30 %
10 %
0 %
0.16
0.12
0.08
0.04
0.00
Absorbance
350
400
450
500
550
Wavelength (nm)

(b)

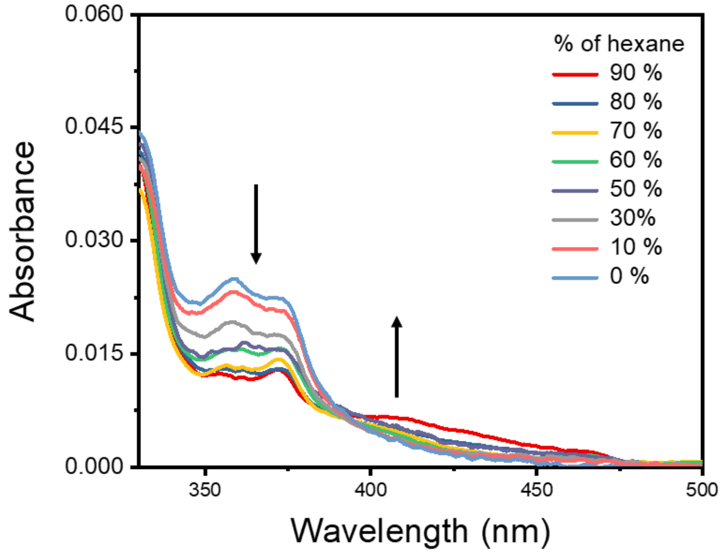
% of hexane
90 %
80 %
70 %
60 %
50 %
30%
10 %
0 %
0.060
0.045
0.030
0.015
0.000
Absorbance
350
400
450
500
Wavelength (nm)

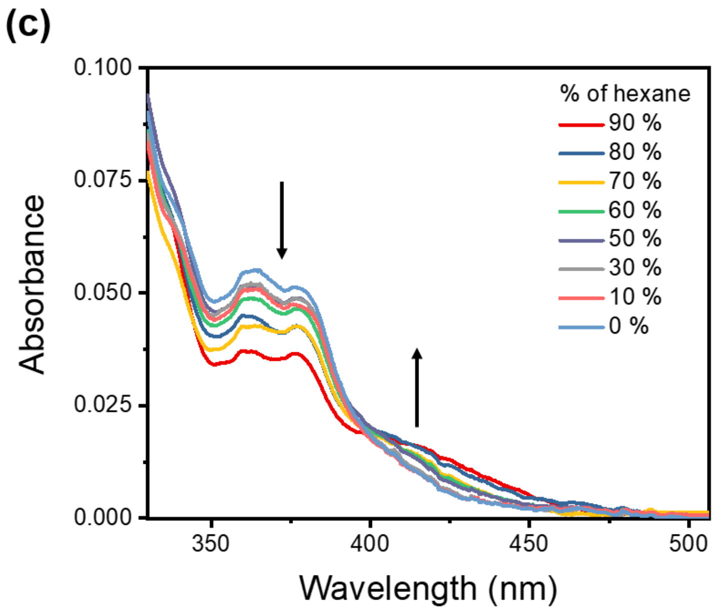

Figure S9. Absorption spectra of complexes (a) **3b**, (b) **5a** and (c) **5b** in THF solution at different volume fractions of hexane (from 0% to 90%), [**3b**] = [**5a**] = [**5b**] = 1.5 × 10^{-5} M.

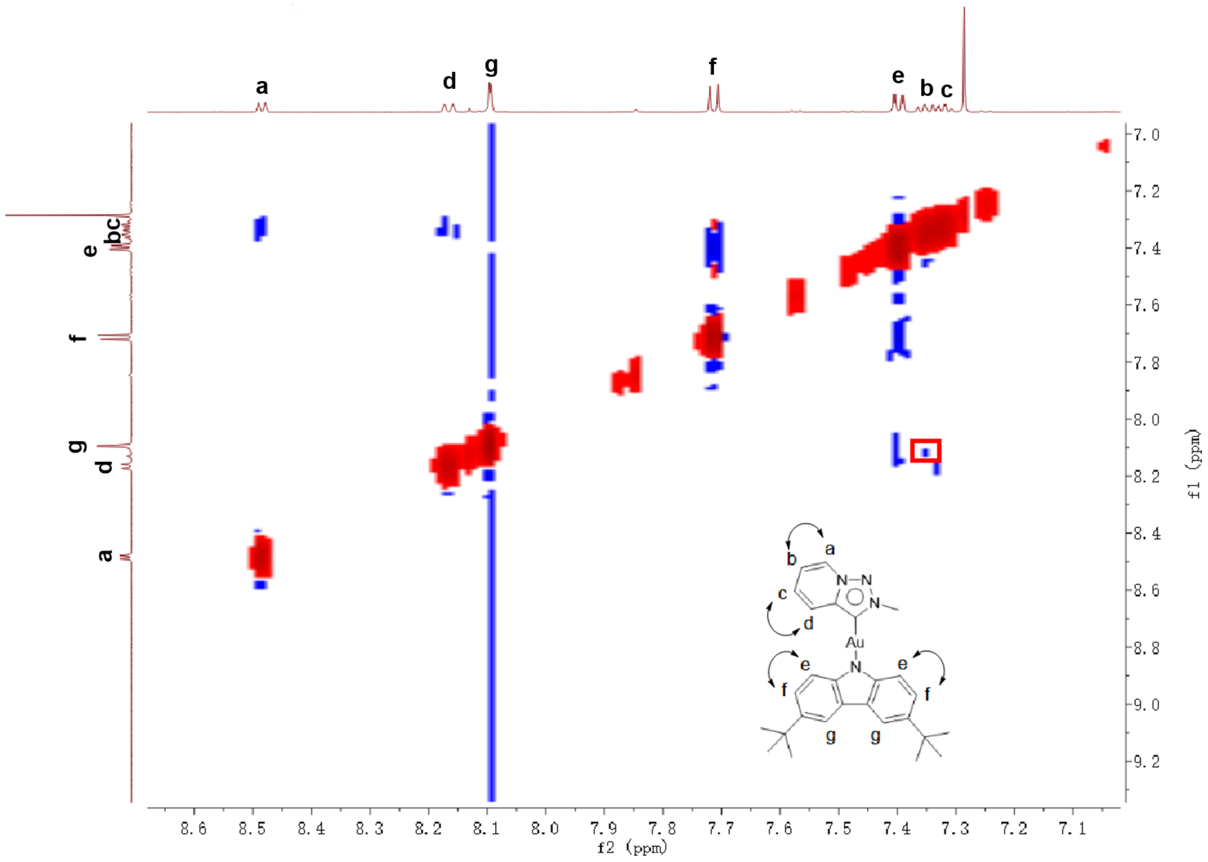

Figure S10. ^1H–^1H NOESY spectrum of **3b**.

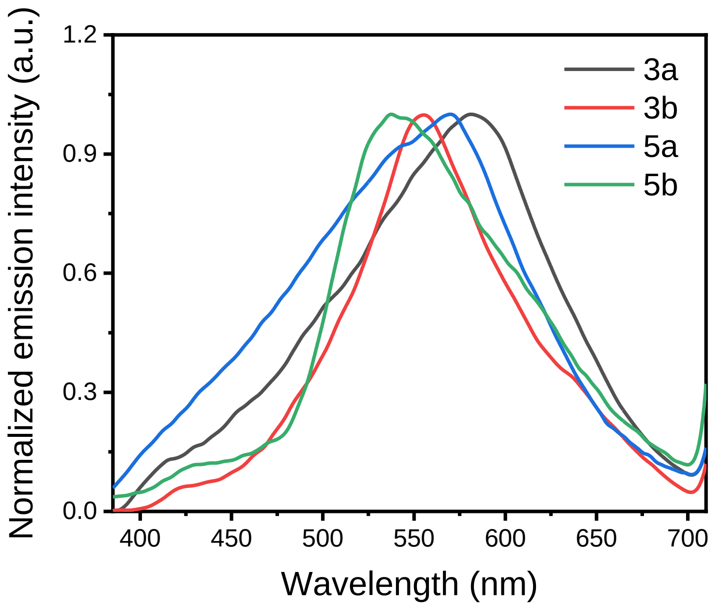

Figure S11. Room temperature PL spectra of complexes **3a–3b** and **5a–5b** in solid state, λ_{ex} = 370 nm.

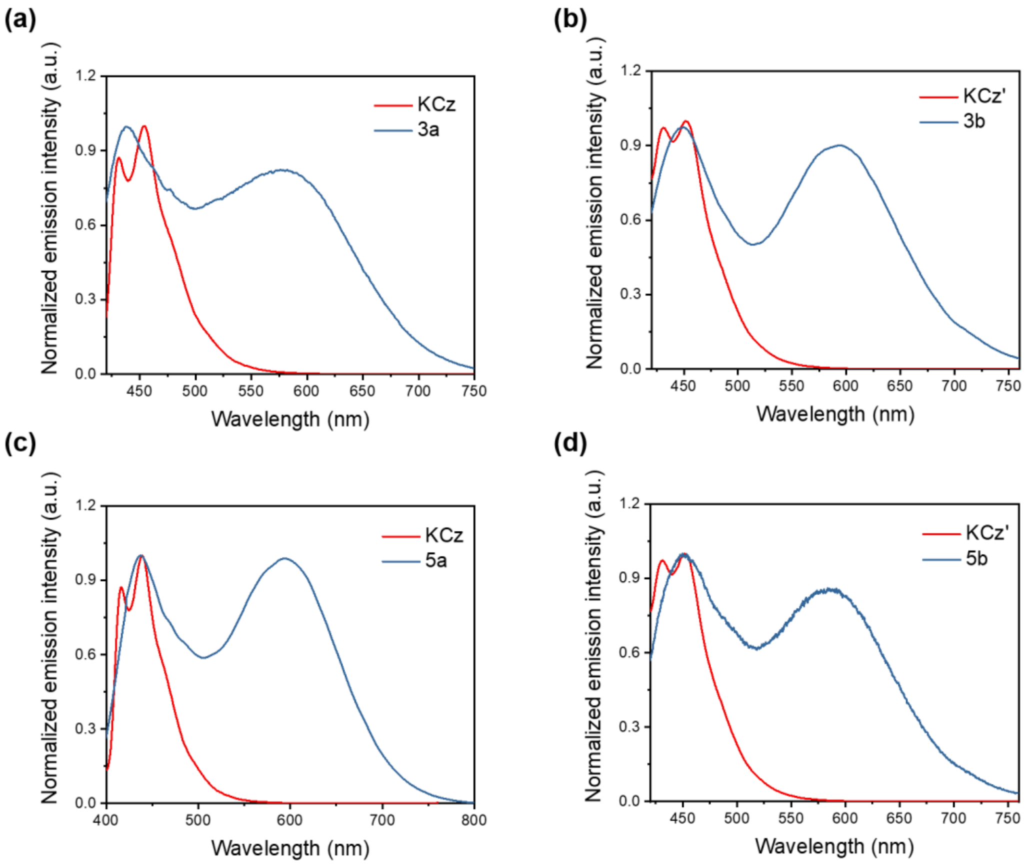

Figure S12. PL emission of complexes (a) **3a** and potassium carbazolide (KCz); (b) **3b** and potassium 3,6-di-tert-butylcarbazolide (KCz'); (c) **5a** and potassium carbazolide (KCz); (d) **5b** and potassium 3,6-di-tert-butylcarbazolide (KCz') in THF, λ_{ex} = 380 nm.

(a)

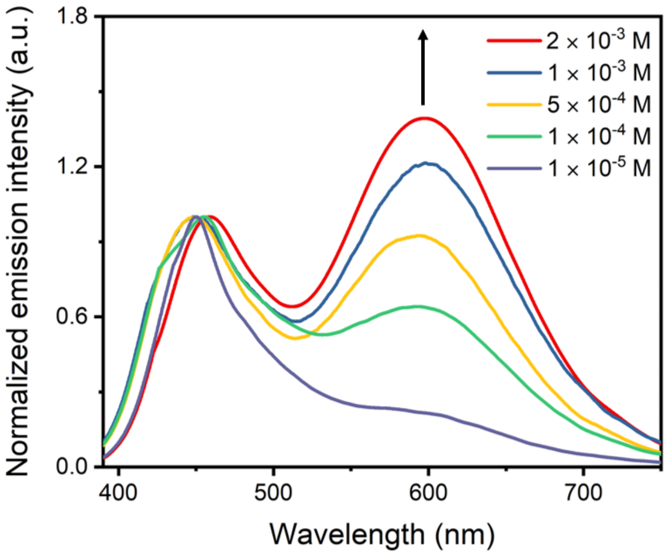
2 × 10-3 M
1 × 10-3 M
5 × 10-4 M
1 × 10-4 M
1 × 10-5 M
Normalized emission intensity (a.u.)
Wavelength (nm)
1.8
1.2
0.6
0.0
400
500
600
700

(b)

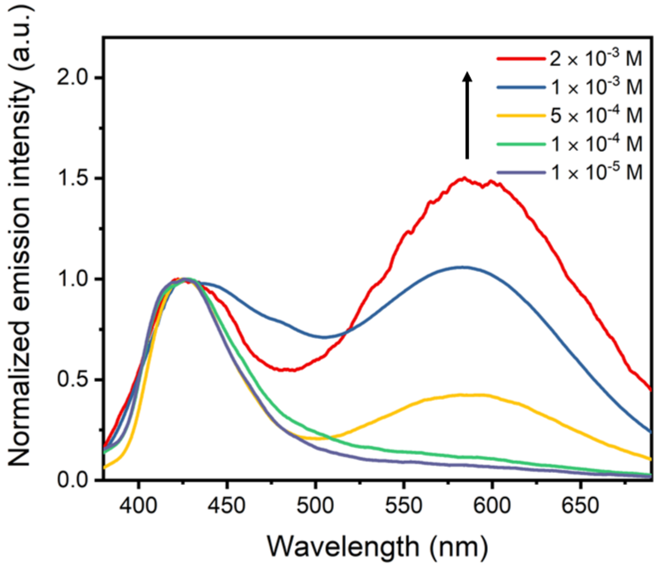
2 × 10-3 M
1 × 10-3 M
5 × 10-4 M
1 × 10-4 M
1 × 10-5 M
Normalized emission intensity (a.u.)
Wavelength (nm)
2.0
1.5
1.0
0.5
0.0
400
450
500
550
600
650

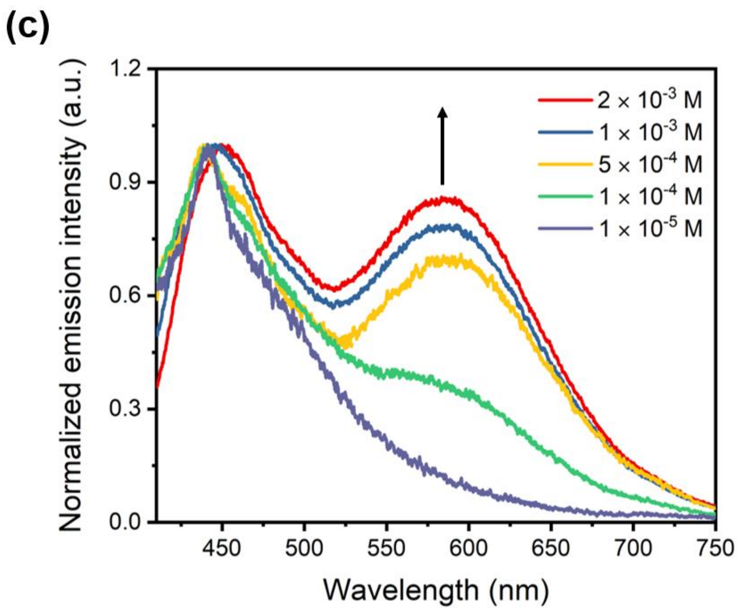

Figure S13. PL spectra of complexes (a) **3b**, (b) **5a** and (c) **5b** in THF solution at different concentration from 1 × 10^{-5} M to 2 × 10^{-3} M, intensity maxima of the blue emission bands were normalized, λ_{ex} = 375 nm.

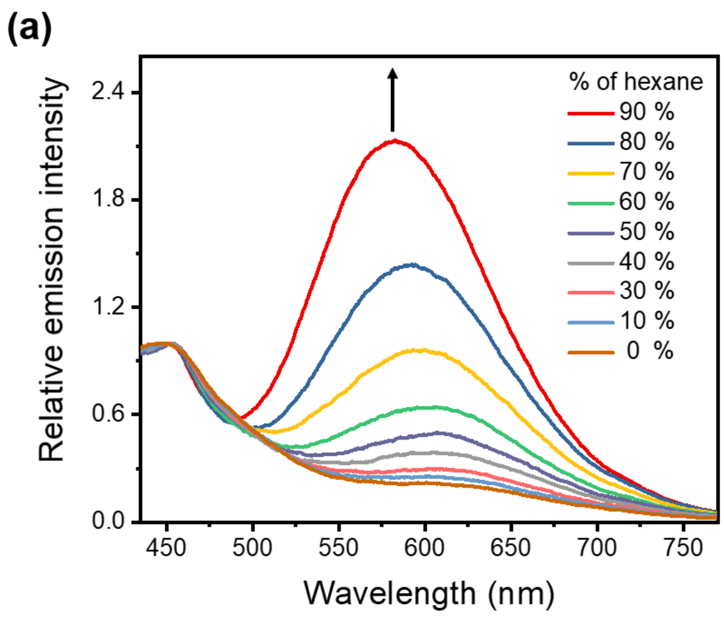

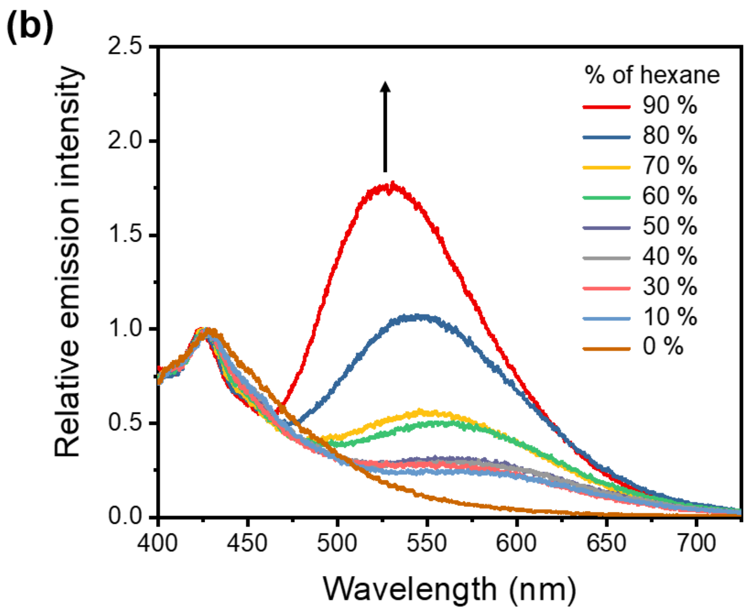

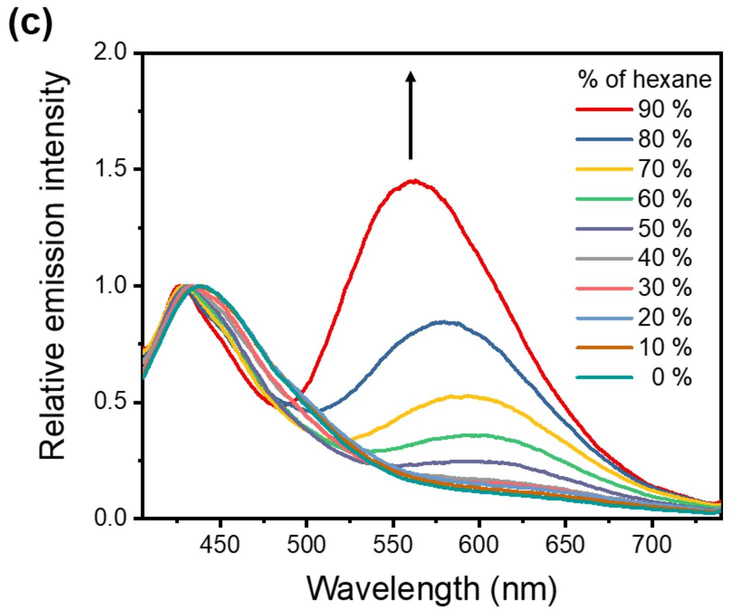

Figure S14. PL spectra of complexes (a) **3b**, (b) **5a** and (c) **5b** in THF solution at different volume fractions of hexane (from 0 to 90%), intensity maxima of the blue emission bands were normalized, [**3b**] = [**5a**] = [**5b**] = 1.5 × 10^{-5} M, λ_{ex} = 375 nm.

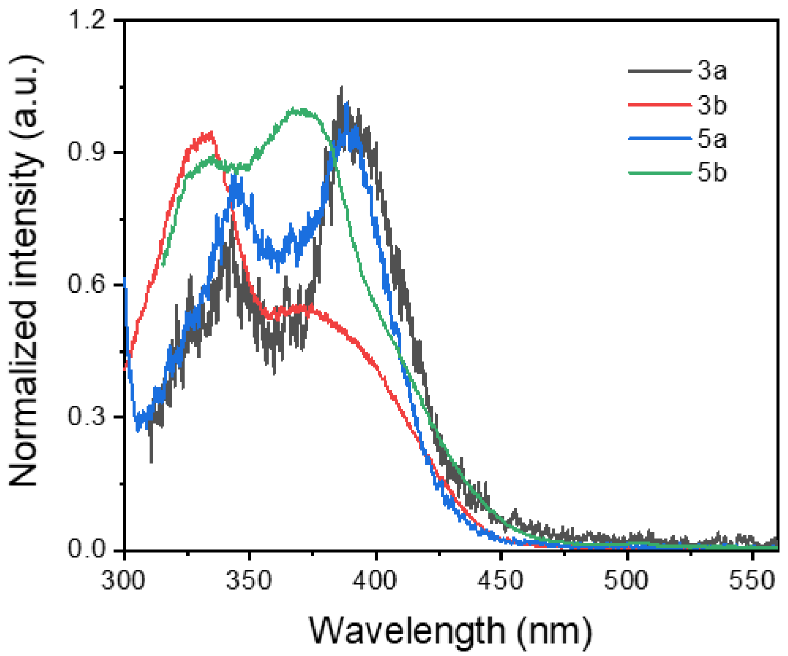

Figure S15. Excitation spectra of complex **3a-3b** and **5a-5b** in THF.

Computational details

Calculations were carried out with the Gaussian 09 package.[6] All the carbene geometry optimizations were performed with the density functional theory (DFT)/B3LYP in the gas phase. The def2-TZVPP basis set was used for all the atoms. Frequency calculations at the same level of theory were performed. Ground /excited states of complexes and time-dependent DFT (TD-DFT) calculation were optimized at the m062x/def2svp level. The dimer obtained with DFT were close to the the X-ray crystal structures. All calculations were treated in vacuo.

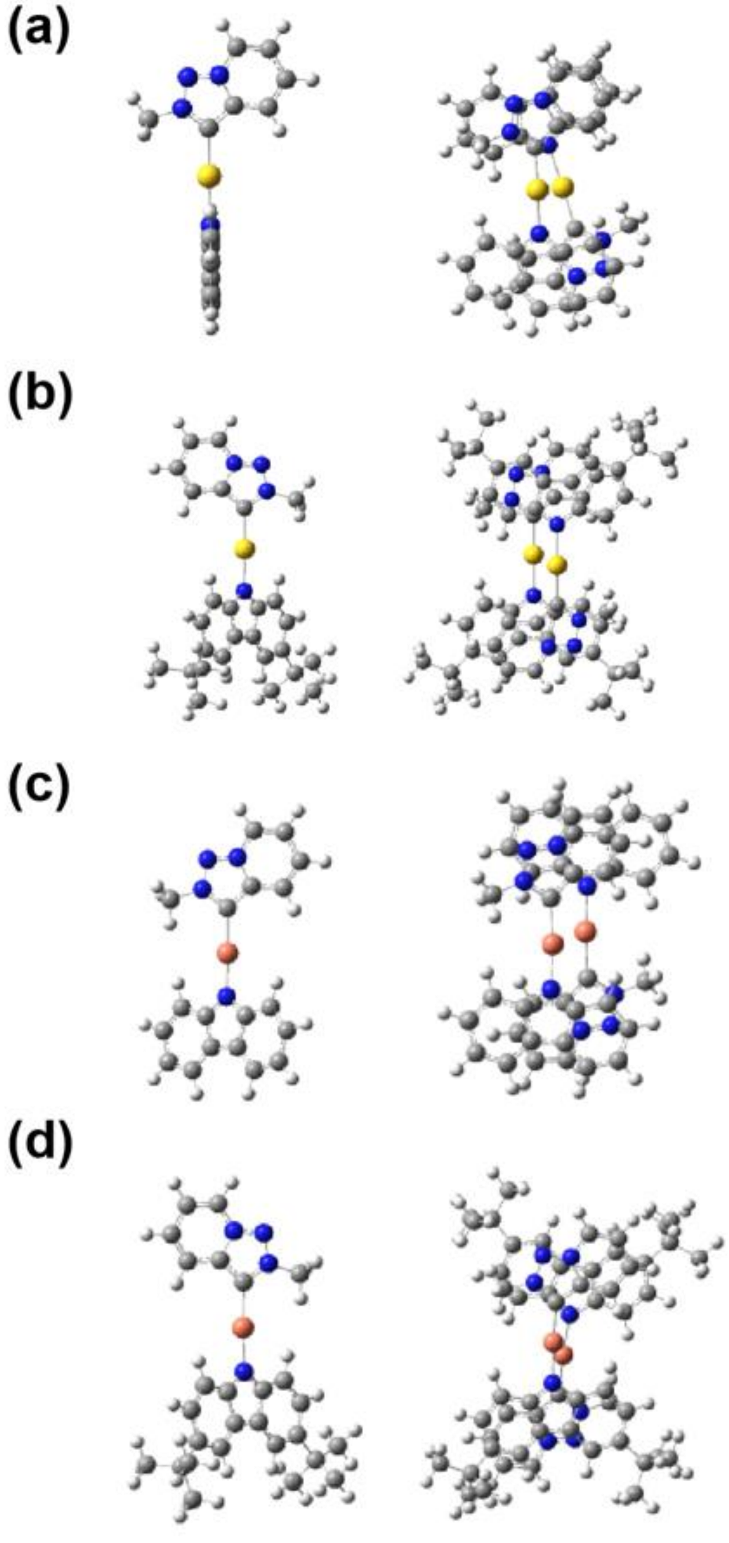

Figure S16. Optimized ground-state structures of the monomer (left) and dimer (right) of (a) **3a**, (b) **3b**, (c) **5a**, (d) **5b**.

Table S8. Comparison of physical parameters between single crystal data and optimized structure from DFT (m062x/def2svp). n.d., not determined.

Complex	Dihedral angle in single crystal	Monomer dihedral angle from DFT	Dimer dihedral angle from DFT	The distance of π-π interaction in single crystal	The distance of π-π interaction from DFT
**3a**	4.61°	89°	29°	3.315 Å	3.388 Å
**3b**	n.d.	50°	0.3°	n.d.	3.371 Å
**5a**	5.91°	37°	0.9°	3.359 Å	3.138 Å
**5b**	0°	34°	2.6°	3.372 Å	3.388 Å

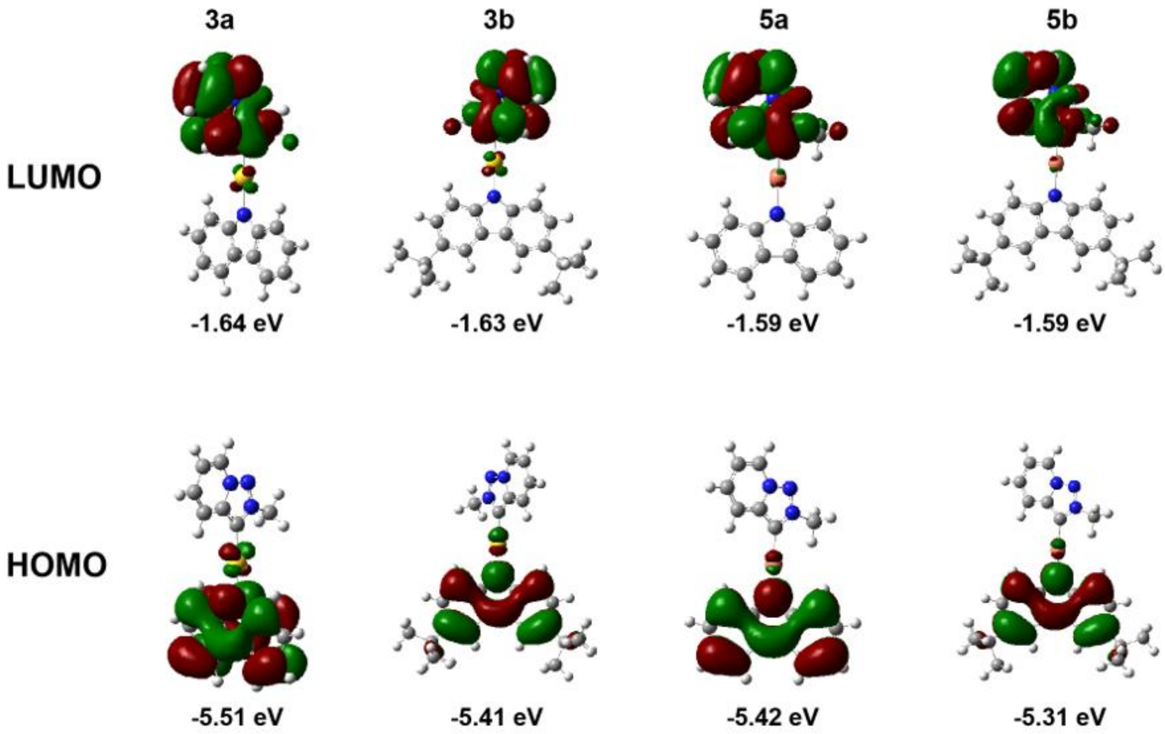

Figure S17. HOMO (bottom) and LUMO (top) of the monomers of complexes **3a-3b** and **5a-5b** and orbital energies *ε* at the DFT m062x/def2svp level of theory. [isovalue = 0.05 (electrons/bohr3)1/2; red (green), positive (negative)].

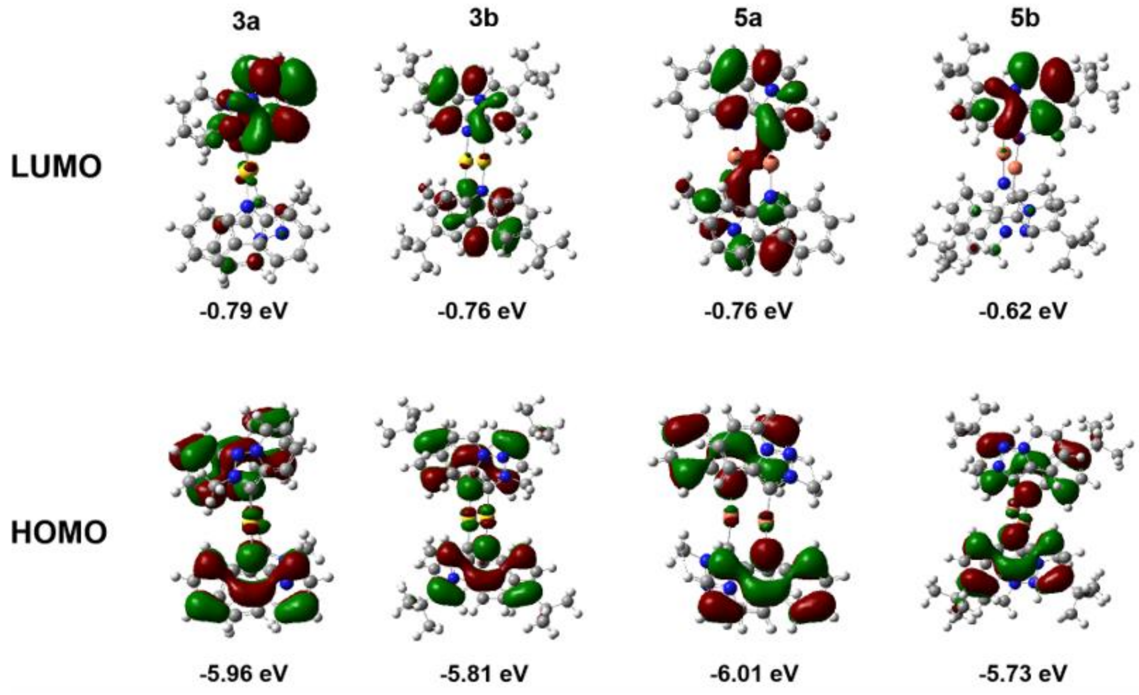

Figure S18. HOMO (bottom) and LUMO (top) of the dimers of complexes **3a-3b** and **5a-5b** and orbital energies *ε* at the DFT m062x/def2svp level of theory. [isovalue = 0.05 (electrons/bohr3)1/2; red (green), positive (negative)].

Table S9. Vertical emission energy transitions of **5a**.

Excited state	Energy (eV)	oscillator strength (*f*)	Major contribution
Excited state 1	2.1508	0.0000	HOMO→LUMO (99%)
Excited state 2	3.1070	0.0000	HOMO-1→LUMO (100%)
Excited state 3	3.1279	0.0000	HOMO→LUMO+1 (100%)
Excited state 4	3.9533	0.0020	HOMO-6→LUMO (7%)<br>HOMO-3→LUMO (82%)<br>HOMO-3→LUMO+2 (7%)
Excited state 5	4.0034	0.0450	HOMO-1→LUMO+6 (4%)<br>HOMO→LUMO+4 (93%)
Excited state 6	4.0627	0.0000	HOMO-1→LUMO+1 (100%)
Excited state 7	4.1993	0.1370	HOMO-16→LUMO+1 (2%)<br>HOMO-5→LUMO (94%)
Excited state 8	4.2414	0.0000	HOMO-2→LUMO (12%)<br>HOMO→LUMO+2 (67%)<br>HOMO→LUMO+3 (15%)
Excited state 9	4.2467	0.0004	HOMO-2→LUMO (60%)<br>HOMO→LUMO+2 (23%)<br>HOMO→LUMO+3 (14%)

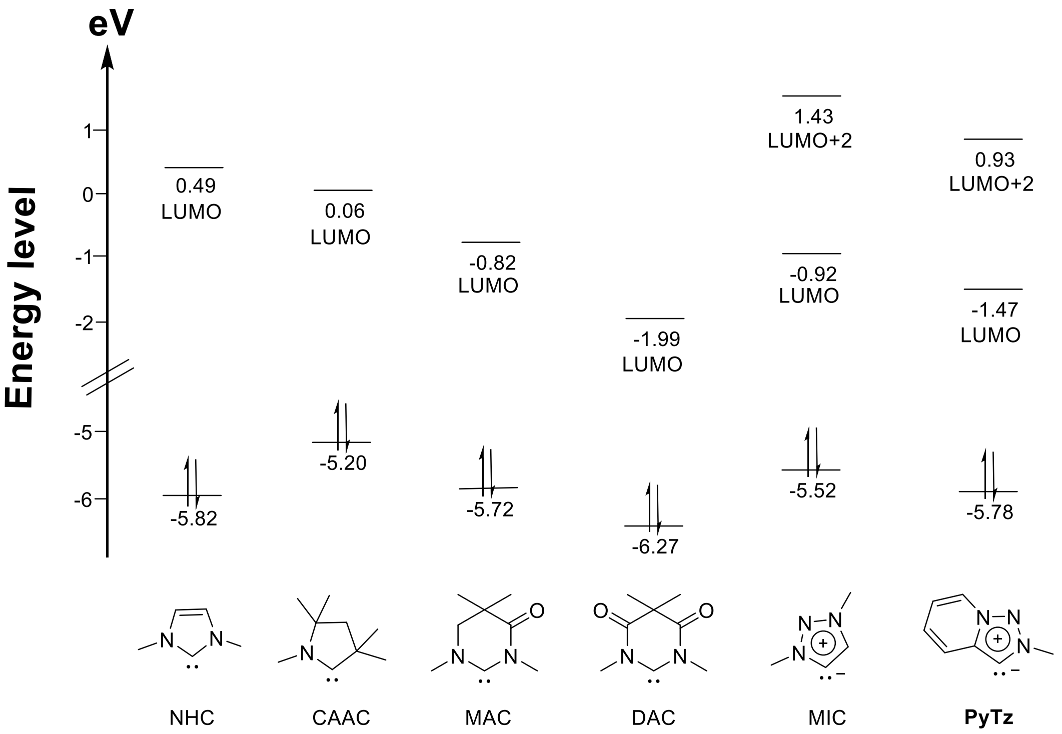

Figure S19. Energy (eV) of frontier orbitals of representative carbenes calculated at B3LYP/def2-TZVPP level.

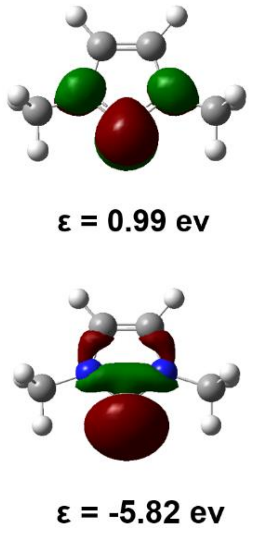

Figure S20. HOMO (bottom) and LUMO + 1 (top) of N-heterocyclic carbene (NHC) and orbital energies *ε* at the DFT B3LYP/def2-TZVPP level of theory. [isovalue = 0.05 (electrons/bohr3)1/2; red (green), positive (negative)].

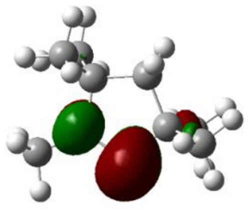

ε = 0.06 ev

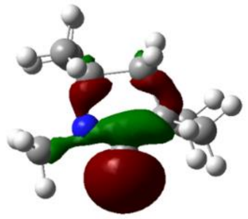

ε = -5.20 ev

Figure S21. HOMO (bottom) and LUMO (top) of cyclic (alkyl)(amino)carbene (CAAC) and orbital energies *ε* at the DFT B3LYP/def2-TZVPP level of theory. [isovalue = 0.05 (electrons/bohr3)1/2; red (green), positive (negative)].

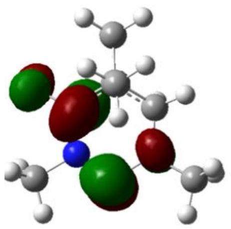

ε = -0.82 ev

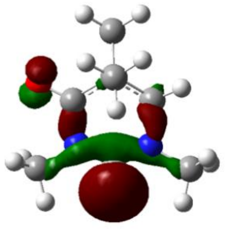

ε = -5.72 ev

Figure S22. HOMO (bottom) and LUMO (top) of cyclic monoamido-aminocarbene (MAC) and orbital energies *ε* at the DFT B3LYP/def2-TZVPP level of theory.[isovalue = 0.05 (electrons/bohr3)1/2; red (green), positive (negative)].

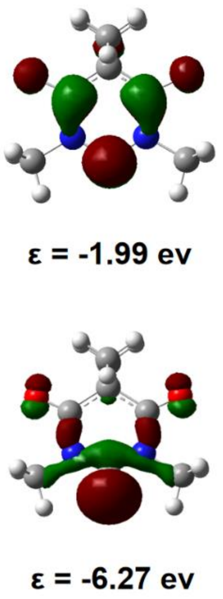

Figure S23. HOMO (bottom) and LUMO (top) of cyclic diamidocarbene (DAC) and orbital energies *ε* at the DFT B3LYP/def2-TZVPP level of theory. [isovalue = 0.05 (electrons/bohr3)1/2; red (green), positive (negative)].

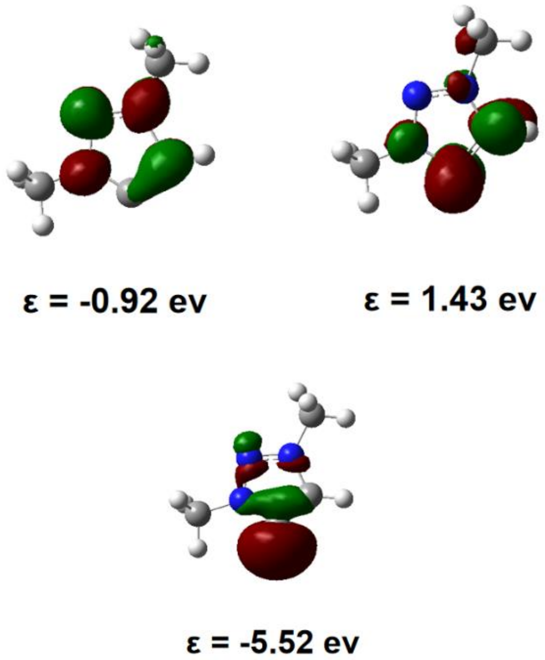

Figure S24. HOMO (bottom) and LUMO (top, left) and LUMO + 2 (top, right) of 1,2,3-triazolylidene and orbital energies *ε* at the DFT B3LYP/def2-TZVPP level of theory. [isovalue = 0.05 (electrons/bohr3)1/2; red (green), positive (negative)].

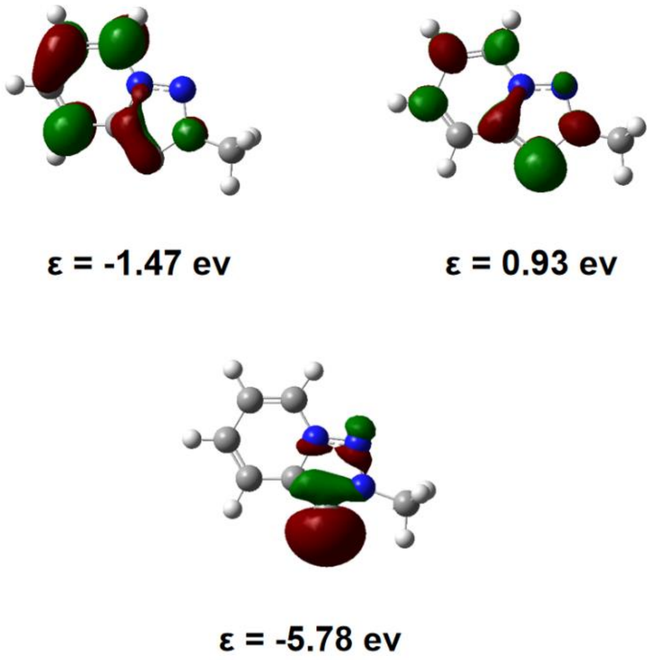

Figure S25. HOMO (bottom) and LUMO (top, left) and LUMO + 2 (top, right) of PyTz and orbital energies *ε* at the DFT B3LYP/def2-TZVPP level of theory. [isovalue = 0.05 (electrons/bohr3)1/2; red (green), positive (negative)].

Cartesian coordinates:

NHC

C	0.00000000	-0.97634600	-0.00012600
N	1.06085100	-0.12118500	-0.00003800
N	-1.06085100	-0.12118500	-0.00005800
C	-2.43935100	-0.57133100	0.00006100
H	-2.43147600	-1.65696900	-0.00012300
H	-2.96429500	-0.21506600	0.88778700
H	-2.96452900	-0.21476200	-0.88740500
C	2.43935200	-0.57133100	0.00007400
H	2.96438700	-0.21475800	0.88762000
H	2.43147700	-1.65696900	0.00025600
H	2.96443800	-0.21506900	-0.88757200
C	0.67584800	1.21064800	0.00003900
H	1.37492000	2.02822400	-0.00029500
C	-0.67584800	1.21064800	-0.00000700
H	-1.37492000	2.02822500	0.00015600

CAAC

C	-0.95866500	-0.57275400	-0.08226100
C	0.44805500	-1.12906200	-0.37288100
C	1.44287200	0.02124900	-0.04025800
C	0.59931700	1.28006400	0.08534700
H	0.52686700	-1.39741500	-1.42728900
H	0.64552600	-2.03366400	0.20263100
C	2.16215400	-0.19866800	1.30338500
H	2.80180200	-1.08267700	1.24906400
H	2.78255300	0.66316800	1.54834000
H	1.45722700	-0.34277000	2.12347100
C	2.48954000	0.20875500	-1.14638000

H	3.15107600	1.04198700	-0.91004300
H	3.09476200	-0.69422000	-1.26048800
H	2.01591500	0.42164000	-2.10636100
C	-1.54544600	-1.10412300	1.23200600
H	-2.50281900	-0.63694900	1.46617100
H	-1.71738600	-2.17851800	1.15294700
H	-0.86734300	-0.92985000	2.06673500
C	-1.93481700	-0.82746500	-1.23368500
H	-2.04008200	-1.90142000	-1.39397700
H	-2.92899000	-0.42997100	-1.02547900
H	-1.57081200	-0.38275600	-2.16062400
N	-0.64839800	0.90484800	0.05023000
C	-1.74591800	1.85982800	0.17288400
H	-2.35859300	1.86738400	-0.72928200
H	-2.38466100	1.61384000	1.02232000
H	-1.30880500	2.84130900	0.32131100

MAC

N	1.65684000	0.08169800	0.10871200
N	-0.21560400	1.37921200	0.03977700
C	-0.80166700	2.72246000	-0.03081000
H	0.01779500	3.42124900	-0.15414800
H	-1.49417100	2.79496000	-0.86872600
H	-1.35339200	2.94387800	0.88170700
C	3.09970800	-0.10456400	0.04321800
H	3.54901700	0.83860100	-0.24965200
H	3.49195500	-0.40747600	1.01813400
H	3.35562300	-0.87714400	-0.68547600
C	1.15666100	1.28757500	-0.10162100
C	-0.52311600	-1.07709000	-0.01931500
C	0.87846400	-1.06496500	0.58694600
H	0.81862400	-1.01941100	1.68068100
H	1.40388500	-1.98652000	0.33043400
C	-1.40204900	-2.09066200	0.71713300
H	-2.41106100	-2.08694100	0.31168200
H	-0.98640500	-3.09439000	0.61146400
H	-1.47036300	-1.85782200	1.78048500
C	-0.46621800	-1.41843100	-1.52297200
H	-0.08003500	-2.43060300	-1.65771200
H	-1.46369000	-1.37329000	-1.95878000
H	0.17871700	-0.73156400	-2.07088000
C	-1.12370000	0.32327200	0.10302700
O	-2.31920500	0.52181800	0.16721700

DAC

N	1.15084900	1.09961400	-0.00016200
N	-1.15106000	1.09942000	0.00010800
C	-2.41266200	1.85541700	0.00003100
H	-2.15118900	2.90693900	0.00081900
H	-2.99962600	1.60667000	-0.88226100
H	-3.00046000	1.60546100	0.88140300
C	2.41230600	1.85584700	-0.00016800
H	2.15062900	2.90731800	-0.00010500
H	2.99974200	1.60658400	0.88166400
H	2.99973200	1.60664100	-0.88200400
C	-0.00016400	1.81493700	0.00011200
C	0.00007300	-1.11944600	0.00014700
C	0.00018900	-2.00687800	1.26593700
H	-0.88973900	-2.63237200	1.26650600
H	0.89027200	-2.63214500	1.26644500

H	0.00011900	-1.40515400	2.17585700
C	0.00009300	-2.00898000	-1.26409100
H	0.89011900	-2.63432600	-1.26364900
H	-0.88989500	-2.63437100	-1.26358500
H	0.00006700	-1.40875500	-2.17499800
C	-1.28020900	-0.29659700	-0.00037300
O	-2.37150700	-0.81718900	-0.00095600
C	1.28026300	-0.29640700	-0.00033700
O	2.37180300	-0.81694700	-0.00070200

MIC

C	-0.74690400	1.34789600	-0.00872700
C	0.64377200	1.25899100	0.00509000
H	1.39384500	2.03104600	0.01119800
C	2.34981800	-0.62525900	-0.00857400
H	2.58208500	-1.00014500	-1.00416000
H	3.06010300	0.15113700	0.26197400
H	2.40741400	-1.44094300	0.70733400
C	-2.38885300	-0.55611300	0.00045300
H	-3.08160800	0.26611000	-0.13823400
H	-2.49327000	-1.28033600	-0.80538400
H	-2.58358200	-1.04845200	0.95250500
N	1.01430000	-0.05263600	0.01487900
N	-0.02643600	-0.84904900	0.00597100
N	-1.04957600	0.01147000	-0.00866100

PyTz

C	-0.17636300	0.79484500	0.00000100
C	-1.25049600	-1.39358400	-0.00000200
C	-2.46568700	-0.78063900	-0.00001200
C	-2.57439900	0.63346100	-0.00000400
C	-1.44246400	1.40498400	0.00000200
C	1.15117300	1.27886100	0.00000600
H	-1.08854700	-2.45944800	-0.00000700
H	-3.35331000	-1.39652700	-0.00002000
H	-3.55270000	1.09209300	-0.00000500
H	-1.48362700	2.48444700	-0.00000300
N	1.81967200	0.10657600	0.00001100
N	1.10706100	-1.05083000	0.00001600
N	-0.13046100	-0.60499900	0.00001100
C	3.26608900	-0.04520400	-0.00001700
H	3.58259300	-0.59173700	-0.88714100
H	3.69194700	0.95146700	0.00003900
H	3.58262100	-0.59187100	0.88701100

3a-monomer

Au	0.29753600	-0.00372100	0.20892500
N	-1.74346300	0.00150800	-0.02464200
N	4.51732900	-0.00691300	0.18730900
N	2.99836100	-0.04013400	1.58348100
N	4.31446600	-0.04000100	1.47800800
C	3.33415100	0.01359100	-0.52971600
C	-2.55823600	1.10801400	-0.09592800
C	-3.92234000	0.72851200	-0.24330500
C	-3.92342800	-0.71640900	-0.26193900
C	2.30055100	-0.00911100	0.42315200
C	5.76035300	0.00728300	-0.40833900
H	6.61147500	-0.01126800	0.26961300
C	-2.55990000	-1.10155900	-0.12442900
C	3.39396900	0.05046400	-1.94617800
H	2.45537000	0.06628400	-2.50023400

C	-4.91869800	1.70884500	-0.33526800
H	-5.96610300	1.42058900	-0.44837600
C	-2.20304500	2.46643000	-0.04230400
H	-1.15602100	2.75849800	0.06949200
C	5.81258000	0.04259900	-1.76671600
H	6.78903800	0.05400700	-2.24950100
C	-4.92120800	-1.69262200	-0.37914200
H	-5.96820000	-1.40003900	-0.48473500
C	4.61831100	0.06456900	-2.55375800
H	4.69504000	0.09275400	-3.64012400
C	-3.20747900	3.41898500	-0.13557000
H	-2.94518900	4.47814300	-0.09572600
C	-4.55898600	3.04915000	-0.28117800
H	-5.32461300	3.82300900	-0.35191400
C	-2.20665200	-2.46140500	-0.10603000
H	-1.16002300	-2.75775600	-0.00207700
C	-4.56342000	-3.03439600	-0.35968700
H	-5.33017400	-3.80506300	-0.45028000
C	-3.21243600	-3.40980800	-0.22382500
H	-2.95163800	-4.47000600	-0.21147900
C	2.41995800	-0.08063000	2.92082100
H	1.84470500	-1.00688600	3.03215000
H	1.74983500	0.77768300	3.04151100
H	3.24004300	-0.04219700	3.64358400

3a-dimer

Au	-0.02079100	-1.46562300	0.88648000
N	-2.01254900	-1.00798200	1.27092500
N	4.14638000	-1.87144100	0.52288600
N	2.47580600	-2.52112500	-0.49483000
N	3.79356800	-2.56908600	-0.52303900
C	3.06130400	-1.38257000	1.22319900
C	-2.47003900	0.18986600	1.75577500
C	-3.89423400	0.25351200	1.71846800
C	-4.31971000	-1.02136200	1.18877300
C	1.92484500	-1.82749300	0.52849000
C	5.45110600	-1.62202300	0.88754800
H	6.21099800	-2.03910400	0.23003900
C	-3.11727300	-1.74902200	0.94118700
C	3.28913100	-0.60562700	2.38833300
H	2.42682700	-0.21433300	2.92728500
C	-4.55798500	1.40907700	2.15431500
H	-5.64875200	1.46471700	2.12505500
C	-1.72379500	1.27800000	2.24640400
H	-0.63282900	1.22023300	2.28606800
C	5.66558400	-0.88500900	2.00954400
H	6.69286200	-0.67825100	2.30760500
C	-5.56306900	-1.60763000	0.91751200
H	-6.48672600	-1.05800400	1.11529600
C	4.57609700	-0.36883900	2.77899200
H	4.78254000	0.23675100	3.66020900
C	-2.40513800	2.40511700	2.68157300
H	-1.83929600	3.25193500	3.07706800
C	-3.81324400	2.48066000	2.63050800
H	-4.31844200	3.38105900	2.98358900
C	-3.17345300	-3.05509700	0.42128500
H	-2.25140000	-3.61408200	0.24477900
C	-5.60840300	-2.89335900	0.39386500
H	-6.57022300	-3.36445600	0.18504200

C	-4.41616300	-3.60775600	0.15131200
H	-4.47529400	-4.62365700	-0.24496900
C	1.75524800	-3.22600500	-1.54163900
H	2.43521900	-3.35203100	-2.38975500
H	0.89350900	-2.61505500	-1.83056100
H	1.41545400	-4.19877500	-1.16291300
Au	-0.03619000	1.34367300	-0.99654400
N	2.03555400	1.18119800	-0.85034300
N	-4.23146300	1.28965700	-1.41373300
N	-2.77820200	2.61126600	-0.78250400
N	-4.08332200	2.49840100	-0.94311500
C	-3.02635900	0.62850300	-1.55753000
C	2.87882000	0.52581400	-1.71015900
C	4.24329100	0.69981800	-1.33078300
C	4.20877500	1.52940800	-0.15026100
C	-2.03838100	1.53207800	-1.13009800
C	-5.44538000	0.72061700	-1.72680100
H	-6.31868700	1.34486300	-1.54969900
C	2.82531000	1.77448200	0.09903200
C	-3.03548000	-0.70293000	-2.04265500
H	-2.08361800	-1.22918000	-2.11772800
C	5.26458800	0.09300000	-2.07493500
H	6.31101200	0.23311800	-1.79213700
C	2.55351200	-0.24393900	-2.84210800
H	1.50963300	-0.35199500	-3.14835200
C	-5.44659100	-0.55096000	-2.20862200
H	-6.40109000	-1.01848000	-2.44675600
C	5.18395600	2.07565500	0.69390500
H	6.24467300	1.90759600	0.49140600
C	-4.22995700	-1.28056300	-2.37072800
H	-4.26715600	-2.30993000	-2.72424900
C	3.58210100	-0.84205800	-3.55599100
H	3.34445000	-1.43904800	-4.43933900
C	4.93100600	-0.68339700	-3.17685300
H	5.71457000	-1.16310500	-3.76474600
C	2.43301700	2.53170800	1.21880200
H	1.37293500	2.71337500	1.41434800
C	4.78691900	2.82820600	1.79213600
H	5.53532700	3.26393100	2.45596000
C	3.41768400	3.04430700	2.05132300
H	3.12677300	3.63892100	2.91956500
C	-2.27680600	3.88889400	-0.30003400
H	-3.02083700	4.30688600	0.38529800
H	-1.33619900	3.70487400	0.22761400
H	-2.11015100	4.56056500	-1.15218900

3b-monomer

Au	-1.59142300	-0.24847400	-0.19016600
N	0.44986500	-0.07222100	-0.07802300
N	-4.31937100	-1.20860800	-1.10262700
N	-5.80677400	-0.25496900	-0.03629100
C	1.17754800	1.09506300	-0.11262700
C	1.65976400	3.44242600	-0.23980000
H	1.29937800	4.47002400	-0.33053800
C	-3.59626500	-0.40552200	-0.28622800
C	0.72553800	2.41998500	-0.22770900
H	-0.34259200	2.63613900	-0.30849500
N	-5.63288500	-1.14429800	-0.97781400
C	3.49282300	1.89296800	-0.02883800

H	4.55602300	1.66253400	0.04892700
C	2.56968400	0.83752100	-0.01403300
C	3.05459700	3.20987600	-0.14118300
C	3.76914900	-1.48110800	0.20615500
H	4.78487700	-1.07745200	0.23828500
C	2.69098700	-0.59964500	0.08648600
C	1.35897000	-1.09850400	0.04228400
C	-4.60870000	0.24521100	0.44187400
C	-4.63682400	1.22034000	1.47074700
H	-3.68646200	1.60469000	1.84137800
C	3.55816400	-2.85939500	0.28419300
C	4.76247300	-3.80201900	0.41514500
C	2.22717900	-3.33147600	0.23957000
H	2.03316500	-4.40222600	0.30060100
C	1.13598000	-2.47928400	0.12125300
H	0.11887400	-2.87809100	0.09329700
C	-3.77337200	-2.14697600	-2.07450800
H	-3.36937300	-3.01982100	-1.54744100
H	-2.96714000	-1.64730900	-2.62172400
H	-4.58168200	-2.44604000	-2.74824900
C	-7.03562200	0.15236800	0.43675200
H	-7.90146100	-0.31736900	-0.02588600
C	4.01215400	4.40710500	-0.16308300
C	-5.84689700	1.63437500	1.95369300
H	-5.89924700	2.38149800	2.74483500
C	-7.05765300	1.08850500	1.42307800
H	-8.02290000	1.41810100	1.80566100
C	5.68555400	-3.62963500	-0.80221700
H	5.14792000	-3.86893500	-1.73169000
H	6.55864600	-4.29684000	-0.72261000
H	6.05388700	-2.59701600	-0.88333900
C	3.69542100	5.34198000	1.01581100
H	2.66372700	5.71885100	0.96499900
H	4.37383500	6.20999300	1.01293700
H	3.81374400	4.81076400	1.97182700
C	5.47524800	3.97084300	-0.04754600
H	5.76658200	3.31670000	-0.88242800
H	5.66091700	3.43239700	0.89343100
H	6.12950200	4.85536400	-0.06627100
C	4.33982500	-5.27241700	0.48846200
H	5.23188900	-5.90989700	0.58129600
C	5.54335500	-3.46437300	1.69575300
H	4.90188400	-3.58189500	2.58169200
H	6.41384200	-4.13089000	1.80434900
H	5.90950100	-2.42791300	1.68092900
C	3.84369900	5.17807800	-1.48265400
H	2.81614100	5.55020000	-1.60346900
H	4.52370900	6.04431800	-1.51276200
H	4.06976600	4.52790300	-2.34071200
H	3.69594700	-5.46391500	1.35964700
H	3.79695600	-5.58145700	-0.41710600

3b-dimer

Au	-0.24629600	-0.71325300	-1.51531500
N	-2.26778300	-0.33598300	-1.20569800
N	2.29739000	-2.30984700	-1.82403600
N	3.89563800	-1.06462400	-2.20611200
C	-2.90115700	0.87972100	-1.21432300
C	-3.20091600	3.25858300	-1.35151400

H	-2.77174000	4.25054600	-1.51635600	C	-7.49282000	-3.25135500	1.09720600
C	1.70387800	-1.09366600	-1.83417800	H	-6.84167200	-3.39544700	1.97389400
C	-2.35865200	2.16058800	-1.42191700	H	-8.41119200	-3.83790900	1.25624600
H	-1.29207200	2.27674400	-1.63379800	H	-7.77676700	-2.18972700	1.04690100
N	3.59800400	-2.32524000	-2.04292900	C	-5.40006800	5.16549100	-2.33407200
C	-5.12233300	1.87531300	-0.89693800	H	-4.37309600	5.43560700	-2.61926200
H	-6.18634000	1.73856000	-0.69714500	H	-5.99201300	6.09276000	-2.27908000
C	-4.29457500	0.74426200	-0.96122100	H	-5.81587300	4.53385000	-3.13273100
C	-4.58892600	3.14852600	-1.07826800	C	-7.73515300	-3.53449800	-1.37903600
C	-5.64102800	-1.46677000	-0.54772100	H	-7.24559200	-3.84904100	-2.31223300
H	-6.61976600	-0.98712300	-0.45192300	H	-8.64212400	-4.14347900	-1.23792300
C	-4.51679900	-0.67355800	-0.79923000	H	-8.04346000	-2.48596600	-1.49872700
C	-3.23347400	-1.27499700	-0.95249600	C	-4.86363600	5.33557500	0.11263300
C	2.79113000	-0.24175100	-2.09327100	H	-3.83949200	5.66017800	-0.12668300
C	2.96744000	1.15804900	-2.24268200	H	-5.48030000	6.24132800	0.22347100
H	2.09783800	1.80416100	-2.12510200	H	-4.84238400	4.80359900	1.07633800
C	-5.52276500	-2.85249200	-0.42039500	Au	0.24625600	0.71285200	1.51555400
C	-6.77922000	-3.70404400	-0.18643600	N	2.26783100	0.33637000	1.20554800
C	-4.23572500	-3.42525000	-0.55595900	N	-2.29752300	2.30876800	1.82567500
H	-4.11524100	-4.50459800	-0.46435000	N	-3.89575100	1.06324400	2.20683800
C	-3.10375100	-2.66616500	-0.82114700	C	2.90154800	-0.87915600	1.21493400
H	-2.12558200	-3.14043500	-0.93269200	C	3.20196500	-3.25784100	1.35367500
C	5.16540000	-0.59354300	-2.45430900	H	2.77308800	-4.24980800	1.51926700
H	5.94814800	-1.34819400	-2.49682900	C	-1.70400200	1.09257800	1.83486000
C	-5.43318500	4.42433500	-0.98786500	C	2.35943100	-2.16002100	1.42353900
C	4.21602400	1.64221000	-2.51423200	H	1.29293300	-2.27630300	1.63576100
H	4.38143800	2.71424200	-2.61742500	N	-3.59812000	2.32398800	2.04464400
C	5.32454300	0.74649500	-2.61944200	C	5.12292700	-1.87436400	0.89779100
H	6.32395000	1.13330000	-2.81383500	H	6.18685400	-1.73746000	0.69767200

C	4.29488100	-0.74349100	0.96149300	H	4.84327800	-4.80376500	-1.07371600
C	4.58988200	-3.14759800	1.08004000	C	7.49182600	3.25139300	-1.10014300
C	5.64065400	1.46762300	0.54624700	H	6.84059300	3.39456100	-1.97691900
H	6.61951300	0.98817800	0.45067000	H	8.41001600	3.83808400	-1.25972200
C	4.51668600	0.67428000	0.79849600	H	7.77608600	2.18989000	-1.04896400
C	3.23321400	1.27546800	0.95151300	C	5.40174300	-5.16368700	2.33678100
C	-2.79124800	0.24045600	2.09328700	H	4.37488900	-5.43387600	2.62232800
C	-2.96757200	-1.15946000	2.24159200	H	5.99388600	-6.09085200	2.28217300
H	-2.09798600	-1.80548500	2.12340600	H	5.81756900	-4.53151500	3.13500800
C	5.52197900	2.85321700	0.41791200	C	-6.45022500	-5.19317800	-0.04370900
C	6.77816100	3.70495000	0.18315900	H	-5.98264100	-5.59207300	-0.95552900
C	4.23479500	3.42571000	0.55322200	H	-5.76996400	-5.37778100	0.80144200
H	4.11399000	4.50495400	0.46081300	H	-7.37470700	-5.76164300	0.13639900
C	3.10307400	2.66650000	0.81914300	C	6.89400800	-4.11727500	0.64385500
H	2.12478200	3.14056700	0.93047500	H	6.97614100	-3.60540400	-0.32696500
C	-5.16549900	0.59198200	2.45474900	H	7.36577500	-3.48429200	1.40942500
H	-5.94823200	1.34661100	2.49791900	H	7.46634100	-5.05463500	0.58120800
C	5.43441300	-4.42325900	0.99016100	C	6.44870700	5.19386400	0.03919600
C	-4.21614300	-1.64382600	2.51283500	H	5.76829500	5.37754400	-0.80603500
H	-4.38157100	-2.71593700	2.61518800	H	5.98110400	5.59340000	0.95072600
C	-5.32464700	-0.74818400	2.61883600	H	7.37299800	5.76245300	-0.14149900
H	-6.32404400	-1.13512600	2.81298900	C	-1.63557500	3.58740500	1.62441300
C	7.73421400	3.53669500	1.37584400	H	-1.11432900	3.87752900	2.54593200
H	7.24461800	3.85188500	2.30880300	H	-0.91760800	3.47621000	0.80523500
H	8.64099800	4.14582200	1.23415900	H	-2.40194700	4.32286700	1.36022400
H	8.04283500	2.48835400	1.49640500	C	-6.89292500	4.11849700	-0.64203900
C	4.86482100	-5.33521100	-0.10972500	H	-7.36465300	3.48600100	-1.40803600
H	3.84077800	-5.65986200	0.12995400	H	-6.97539400	3.60616400	0.32850600
H	5.48163100	-6.24091100	-0.22018300	H	-7.46506500	5.05595300	-0.57905700

C	1.63541200	-3.58831300	-1.62178300
H	0.91794000	-3.47665400	-0.80223200
H	1.11363800	-3.87881600	-2.54287600
H	2.40185500	-4.32372100	-1.35763800

5a-monomer

Cu	0.31730900	-0.19152700	-0.06950800
N	-1.57628300	0.03376000	0.01695800
N	4.50405800	-0.36262900	-0.12250300
N	4.23450600	-1.51898800	-0.66799600
N	2.91280600	-1.54513200	-0.68842300
C	-2.28725500	1.19224900	-0.16878400
C	-2.50066500	-0.95530600	0.23855600
C	3.35576700	0.34013800	0.20260200
C	-3.69146600	0.96649100	-0.06835700
C	-3.83209600	-0.44656800	0.19809700
C	2.26909700	-0.46658400	-0.18333000
C	-2.27599500	-2.32143300	0.48521400
H	-1.25483600	-2.71129400	0.52785400
C	5.77440500	0.11781400	0.10692800
H	6.59132300	-0.53389100	-0.19690000
C	5.89528500	1.34419700	0.68372400
H	6.89489300	1.73483200	0.87062000
C	4.74296500	2.11106000	1.04045600
H	4.87342100	3.08946400	1.50155700
C	-4.91949700	-1.30584600	0.39978100
H	-5.94071700	-0.91936400	0.36920300
C	-1.79827400	2.48441300	-0.43022700
H	-0.72169700	2.65809800	-0.51091300
C	3.48994800	1.61733800	0.80350200
H	2.58244100	2.16486400	1.05823100
C	-4.58811200	2.03068400	-0.22765700
H	-5.66449100	1.86116800	-0.15107800
C	-2.70526800	3.52259900	-0.58425200
H	-2.33747700	4.53040900	-0.78802900
C	-4.68548400	-2.65433600	0.63924800
H	-5.52393400	-3.33387700	0.79822300
C	-4.09357000	3.30333300	-0.48419100
H	-4.78127200	4.14071900	-0.61041900
C	-3.36862800	-3.15348100	0.68133200
H	-3.20633400	-4.21582100	0.87515100
C	2.28234700	-2.74014400	-1.23305700
H	2.71634000	-2.95893500	-2.21488500
H	1.21079800	-2.53645300	-1.32232100
H	2.45295400	-3.58441700	-0.55434000

5a-monomer (Excited state S_1)

Cu	-0.34492800	0.04315100	0.31787600
N	1.61572300	0.00213600	0.06817100
N	-4.50027700	0.04292100	0.21012300
N	-4.34806400	0.22618400	1.49211100
N	-3.02286100	0.26669200	1.66589600
C	2.42832200	1.08416900	-0.11073500
C	2.41044500	-1.10624400	0.03327600
C	-3.27575600	-0.05479400	-0.46164800
C	3.79479400	0.69663700	-0.26617800
C	3.78308100	-0.76389900	-0.17020300
C	-2.28754800	0.08539000	0.51506900
C	2.00424000	-2.44424400	0.16978700
H	0.95267700	-2.68682800	0.32061500

C	-5.71911200	-0.03690500	-0.41909200
H	-6.58700800	0.06084100	0.22371700
C	-5.72800400	-0.23168400	-1.81493100
H	-6.68975200	-0.29613800	-2.32014300
C	-4.53961200	-0.33668000	-2.52440600
H	-4.57197300	-0.48606900	-3.60391000
C	4.74094000	-1.75557100	-0.23433000
H	5.79411800	-1.51802700	-0.38907800
C	2.04314100	2.43434500	-0.15152500
H	0.99556500	2.71096800	-0.03481100
C	-3.29622700	-0.25265100	-1.87624600
H	-2.35481000	-0.33118700	-2.41120900
C	4.76814200	1.65623900	-0.45897500
H	5.81728100	1.38385500	-0.58026800
C	3.03895200	3.38910800	-0.34704300
H	2.77286500	4.44493300	-0.38473600
C	4.32865200	-3.09687100	-0.09577400
H	5.07491700	-3.88985700	-0.14535900
C	4.37719000	3.01030900	-0.49790200
H	5.13576900	3.77817300	-0.65004700
C	2.98481600	-3.43170500	0.10217700
H	2.70232500	-4.47907400	0.20308600
C	-2.52961700	0.34829600	3.01167900
H	-3.28288100	0.84787400	3.63173200
H	-1.60252900	0.93450100	3.01836900
H	-2.32634500	-0.65035900	3.43153100

5a-dimer

Cu	0.51597300	-1.38982700	0.85751600
N	2.41615200	-1.05970100	0.87693100
N	-3.56564800	-2.29683600	1.14762200
N	-3.11564000	-3.41195800	0.63128700
N	-1.81936400	-3.19560200	0.53046100
C	3.07484200	0.02157300	1.39793100
C	3.38664100	-1.91271800	0.41587700
C	-2.55300500	-1.38409000	1.38002800
C	4.48836000	-0.11248300	1.27608800
C	4.69528500	-1.39182200	0.63975300
C	-1.36241900	-1.99066100	0.94567300
C	3.22374800	-3.12670200	-0.27381300
H	2.22131600	-3.51878800	-0.46492200
C	-4.89082700	-2.04632000	1.42392000
H	-5.58497600	-2.84662200	1.17687100
C	-5.20905100	-0.83006600	1.94293800
H	-6.25556100	-0.60489100	2.14608700
C	-4.20376400	0.15352100	2.19137500
H	-4.49743900	1.13874900	2.55656800
C	5.82142100	-2.09285800	0.19049100
H	6.82496500	-1.69590300	0.36262300
C	2.52282000	1.18459600	1.96861100
H	1.43762600	1.27834100	2.06782300
C	-2.89206200	-0.12202000	1.92486400
H	-2.09956800	0.61059800	2.07195900
C	5.33392400	0.92043800	1.70311300
H	6.41821000	0.82195300	1.60839000
C	3.37934400	2.19473600	2.38026400
H	2.96677300	3.10417700	2.82357000
C	5.64747300	-3.29298100	-0.48877600
H	6.51520200	-3.85106100	-0.84326300

C	4.77843800	2.07324700	2.24370700
H	5.42359000	2.88631800	2.57917400
C	4.35281500	-3.80018200	-0.71956000
H	4.23741000	-4.74428800	-1.25569200
C	-1.01899000	-4.23465800	-0.09292800
H	-1.56719200	-5.17986100	-0.02992900
H	-0.06384400	-4.30490600	0.43897000
H	-0.84450800	-3.97241200	-1.14540000
Cu	-0.51597300	1.38981400	-0.85766500
N	-2.41614100	1.05957200	-0.87722800
N	3.56563900	2.29698000	-1.14739500
N	3.11560200	3.41207400	-0.63102300
N	1.81932100	3.19570400	-0.53026300
C	-3.07489300	-0.02173800	-1.39806400
C	-3.38657600	1.91263900	-0.41614300
C	2.55300500	1.38425900	-1.37994000
C	-4.48839600	0.11230400	-1.27601300
C	-4.69524600	1.39170100	-0.63977400
C	1.36239600	1.99080000	-0.94559500
C	-3.22360900	3.12671400	0.27337200
H	-2.22115700	3.51884000	0.46429600
C	4.89083500	2.04648300	-1.42363400
H	5.58496700	2.84677000	-1.17649000
C	5.20908700	0.83026500	-1.94272000
H	6.25560800	0.60510200	-2.14582600
C	4.20381300	-0.15330400	-2.19128000
H	4.49750800	-1.13851500	-2.55650600
C	-5.82133000	2.09277800	-0.19044500
H	-6.82489100	1.69578600	-0.36239400
C	-2.52294300	-1.18478000	-1.96877300
H	-1.43776400	-1.27851300	-2.06814600
C	2.89209300	0.12222600	-1.92484200
H	2.09960700	-0.61037800	-2.07204200
C	-5.33401200	-0.92065200	-1.70285000
H	-6.41828400	-0.82217700	-1.60796100
C	-3.37951600	-2.19495800	-2.38023000
H	-2.96701500	-3.10440000	-2.82359900
C	-5.64730600	3.29299100	0.48864200
H	-6.51499400	3.85110700	0.84317300
C	-4.77859100	-2.07348400	-2.24346200
H	-5.42378000	-2.88658700	-2.57878000
C	-4.35262400	3.80023800	0.71918300
H	-4.23715500	4.74441600	1.25517400
C	1.01891700	4.23472300	0.09314600
H	1.56709700	5.17994200	0.03018200
H	0.06378300	4.30496800	-0.43877000
H	0.84442300	3.97245200	1.14561100

5b-monomer

Cu	-1.82589000	-0.27549100	-0.09423400
N	0.06966100	-0.07769400	-0.00574000
N	-4.42090200	-1.55174600	-0.84625800
N	-6.01386400	-0.45164400	-0.13736600
C	0.80017300	1.08247300	-0.05761800
C	1.27383700	3.43467700	-0.18351200
H	0.90862100	4.46243900	-0.25156800
C	-3.77883700	-0.53561600	-0.22311400
C	0.34405500	2.40918600	-0.14472000
H	-0.72750300	2.62335400	-0.18256300

N	-5.74251400	-1.53537100	-0.81496600
C	3.11730800	1.88702900	-0.05475700
H	4.18352700	1.65966100	-0.01905200
C	2.19821400	0.82880400	-0.01393500
C	2.67217100	3.20406300	-0.13946900
C	3.39701000	-1.49840700	0.13912500
H	4.41626300	-1.10206100	0.13572400
C	2.32110500	-0.60899300	0.06931600
C	0.98283600	-1.09936600	0.07139600
C	-4.86672700	0.21486800	0.26038800
C	-5.00338000	1.41291800	1.00627100
H	-4.09695100	1.93227000	1.31745300
C	3.18095900	-2.87671900	0.21269300
C	4.38418100	-3.82696300	0.28779800
C	1.84655300	-3.34184400	0.21673200
H	1.64918600	-4.41206900	0.27783800
C	0.75783500	-2.48118500	0.14879800
H	-0.26294300	-2.87429800	0.16349800
C	-3.78800900	-2.67334800	-1.52725600
H	-3.92918700	-3.58630300	-0.93623900
H	-2.72181900	-2.44442700	-1.62030800
H	-4.24434700	-2.79596300	-2.51555800
C	-7.28503500	-0.00795200	0.15347800
H	-8.10073500	-0.62443100	-0.21935400
C	3.62562900	4.40394600	-0.18728600
C	-6.25720700	1.86893200	1.30621800
H	-6.38928200	2.78651500	1.87851100
C	-7.40814900	1.14241300	0.86949300
H	-8.40838700	1.50327600	1.10617400
C	5.25988500	-3.64871700	-0.96325700
H	4.68506700	-3.87765400	-1.87290300
H	6.13243000	-4.32029200	-0.92366300
H	5.62935900	-2.61699400	-1.05018400
C	3.35247800	5.32819700	1.01075500
H	2.31871600	5.70278200	1.00278100
H	4.02830900	6.19803700	0.98949600
H	3.50844200	4.78910500	1.95691400
C	5.09308300	3.97055800	-0.13132900
H	5.35367200	3.32421900	-0.98229800
H	5.31565600	3.42438300	0.79708700
H	5.74426600	4.85686700	-0.16727900
C	3.95652300	-5.29576100	0.36453500
H	4.84787500	-5.93898000	0.41554900
C	5.21711200	-3.50574100	1.53952000
H	4.61027300	-3.62758700	2.44896700
H	6.08727200	-4.17809000	1.60777400
H	5.58848200	-2.47124800	1.51949900
C	3.40601400	5.18621800	-1.49267000
H	2.37386800	5.55705600	-1.57120800
H	4.08275100	6.05425600	-1.54106500
H	3.60056700	4.54389400	-2.36427300
H	3.34702500	-5.49199700	1.25911000
H	3.37595100	-5.59329300	-0.52132600

5b-dimer

Cu	-0.16397700	0.83919000	-1.31369700
N	1.69412400	0.39520700	-0.93326000
N	-2.68286300	2.42576100	-1.92607200
N	-4.18818300	1.07492000	-2.32264900

C	2.21209100	-0.87423600	-0.92268000
C	2.26001100	-3.27717900	-0.88592200
H	1.71707200	-4.22643500	-0.89650400
C	-1.99136400	1.26360800	-1.95874500
C	1.52450200	-2.10171000	-0.91841200
H	0.43072500	-2.11749100	-0.95123300
N	-3.98607800	2.35197900	-2.13815800
C	4.34998500	-2.07534300	-0.85098200
H	5.44013500	-2.03897100	-0.83002500
C	3.63418400	-0.87089700	-0.89336200
C	3.67842800	-3.29666000	-0.84855000
C	5.22537700	1.21241100	-0.97144900
H	6.15950400	0.64308200	-0.98699800
C	4.00914500	0.52424800	-0.92360300
C	2.77395400	1.23970200	-0.94026100
C	-3.01618500	0.34436000	-2.24111700
C	-3.07382900	-1.05887000	-2.43679100
H	-2.14838800	-1.62972800	-2.36714500
C	5.25316400	2.60869200	-1.02054500
C	6.59852400	3.33753000	-1.15503500
C	4.01854000	3.29949900	-1.00622300
H	4.01114600	4.38881800	-1.04389400
C	2.79505500	2.64267300	-0.96889600
H	1.85845900	3.20612300	-0.97817900
C	-5.41855000	0.49973900	-2.54838500
H	-6.26596600	1.18223600	-2.56372200
C	4.41352900	-4.64325900	-0.84270100
C	-4.28173600	-1.64514200	-2.68934100
H	-4.35169000	-2.72316800	-2.83080200
C	-5.46633100	-0.84692000	-2.73659000
H	-6.43466900	-1.31201900	-2.91825000
C	7.52340500	2.97473700	0.01692300
H	7.09865600	3.32996300	0.96862600
H	8.51047000	3.44620800	-0.11117900
H	7.67410100	1.88766200	0.09136400
C	4.04545400	-5.43335600	-2.10926900
H	2.96547700	-5.63309200	-2.16057200
H	4.57025200	-6.40161200	-2.12595200
H	4.32665400	-4.86917700	-3.01055100
C	7.26936600	2.91339900	-2.47262300
H	6.62224900	3.15418800	-3.32864300
H	8.23059800	3.43537400	-2.60326900
H	7.46307000	1.83133400	-2.49236400
C	4.01328800	-5.45687000	0.39829500
H	2.93035900	-5.65329200	0.42090500
H	4.52680400	-6.43103500	0.40380000
H	4.29094600	-4.91626300	1.31639900
Cu	0.22010300	-0.35237800	1.47045400
N	-1.65556000	-0.16230700	0.97298300
N	2.59660000	-1.90511300	2.38965700
N	4.25997200	-0.69111700	2.46286700
C	-2.41098000	0.97181800	1.12921000
C	-2.92849900	3.29432200	1.46537000
H	-2.58379700	4.31328200	1.66171500
C	2.04172600	-0.68683400	2.19082700
C	-1.97979100	2.28719700	1.37640200
H	-0.91398600	2.50339700	1.49564300
N	3.90828100	-1.94614900	2.55214200

C	-4.73842900	1.74999500	1.07955300	H	-3.95299100	5.67410400	0.44512000
H	-5.79775000	1.51606000	0.96291100	H	-5.67096400	6.11895600	0.37467100
C	-3.80216700	0.71050500	0.98972500	H	-5.04910600	4.81208000	-0.67281000
C	-4.31883600	3.05780300	1.31269500	C	-6.86345600	-3.51978900	-0.81673800
C	-4.95884900	-1.60739100	0.59548200	H	-6.36610400	-3.57592400	-1.79727000
H	-5.98487800	-1.23030800	0.63959500	H	-7.74036200	-4.18548000	-0.84276900
C	-3.89787100	-0.71194000	0.75783100	H	-7.22545400	-2.49127400	-0.67266400
C	-2.54843000	-1.17870000	0.74831600	C	-5.17286000	4.89254600	2.79817600
C	3.17557300	0.14220200	2.25298700	H	-4.15506200	5.26765000	2.97769000
C	3.40208300	1.53798700	2.15138200	H	-5.86748400	5.74300000	2.88445000
H	2.54637500	2.18765000	1.97245700	H	-5.40928100	4.16809000	3.59126400
C	-4.71874400	-2.96964000	0.39757000	C	6.43285900	4.86015600	-1.17124600
C	-5.90211200	-3.94439500	0.30415300	H	5.82774400	5.19121900	-2.02764900
C	-3.37450800	-3.40784200	0.35766200	H	5.95393200	5.22436700	-0.24969500
H	-3.15737100	-4.46465900	0.20278400	H	7.41971400	5.33984600	-1.25136000
C	-2.29973200	-2.54376000	0.53153200	C	-6.74750000	3.78874400	1.22066600
H	-1.27410200	-2.92233000	0.51415900	H	-6.89669400	3.33275100	0.23036100
C	5.55467300	-0.23276700	2.55488300	H	-7.05010600	3.05997700	1.98665600
H	6.31790500	-0.99393100	2.70348800	H	-7.41744300	4.65797200	1.29733600
C	-5.29574100	4.23526400	1.41407900	C	-5.44833900	-5.37903000	0.01762400
C	4.67788000	2.01553900	2.26202200	H	-4.88395600	-5.44304300	-0.92512800
H	4.87671500	3.08305600	2.17195900	H	-4.81401800	-5.77130800	0.82560500
C	5.76318900	1.10856900	2.46300400	H	-6.32659500	-6.03610200	-0.06794300
H	6.78401400	1.48114100	2.53780000	C	1.88121400	-3.16726400	2.48380100
C	-6.66079500	-3.93653500	1.64231700	H	1.94178500	-3.54154500	3.51362400
H	-5.99269000	-4.23066700	2.46491200	H	0.83907500	-2.97493500	2.21096400
H	-7.50853700	-4.63939400	1.61207100	H	2.33005400	-3.88491100	1.79009500
H	-7.05394300	-2.93543500	1.87102800	C	5.93381000	-4.46195500	-0.81458000
C	-4.97022700	5.27075000	0.32515000	H	6.29035900	-3.91356300	-1.69857100

H	6.25468000	-3.91590200	0.08516100
H	6.42520100	-5.44620300	-0.80651800
C	-2.11800600	3.75197100	-1.74124500
H	-1.17336700	3.63720500	-1.20187100
H	-1.94373900	4.21460100	-2.72164500
H	-2.81832600	4.35189700	-1.15201300

Thermally activated delayed fluorescence (TADF) studies

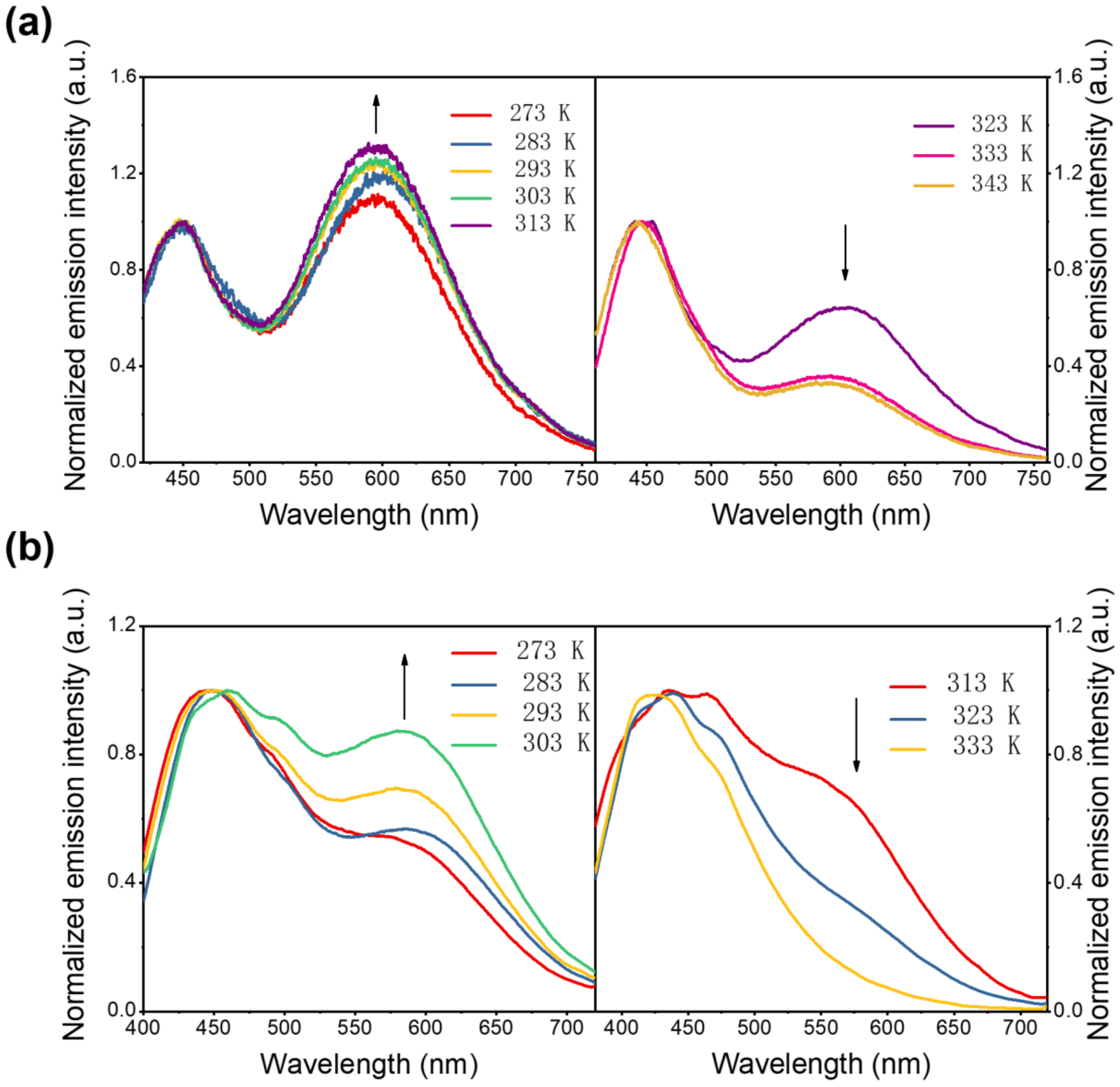

Figure S26. PL spectra of complexes (a) **3b**, (b) **5b** in THF solution measured at different temperatures, intensity maxima of the blue emission bands were normalized, [**3b**] = [**5b**] = 1 × 10^{-3} M, λ_{ex} = 380 nm.

(a)

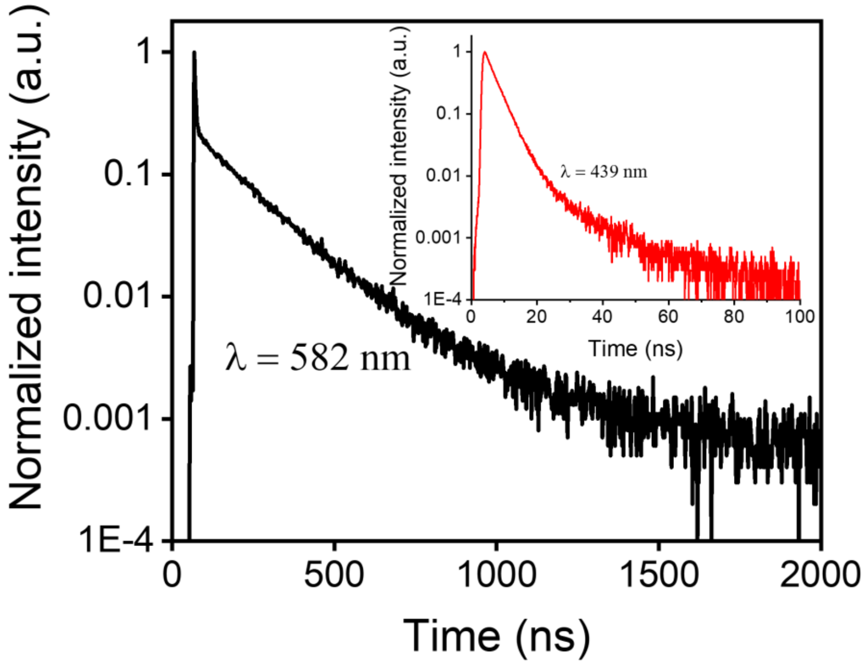
Normalized intensity (a.u.)
1
0.1
0.01
0.001
1E-4
0
500
1000
1500
2000
Time (ns)
λ = 582 nm
λ = 439 nm
0
20
40
60
80
100

(b)

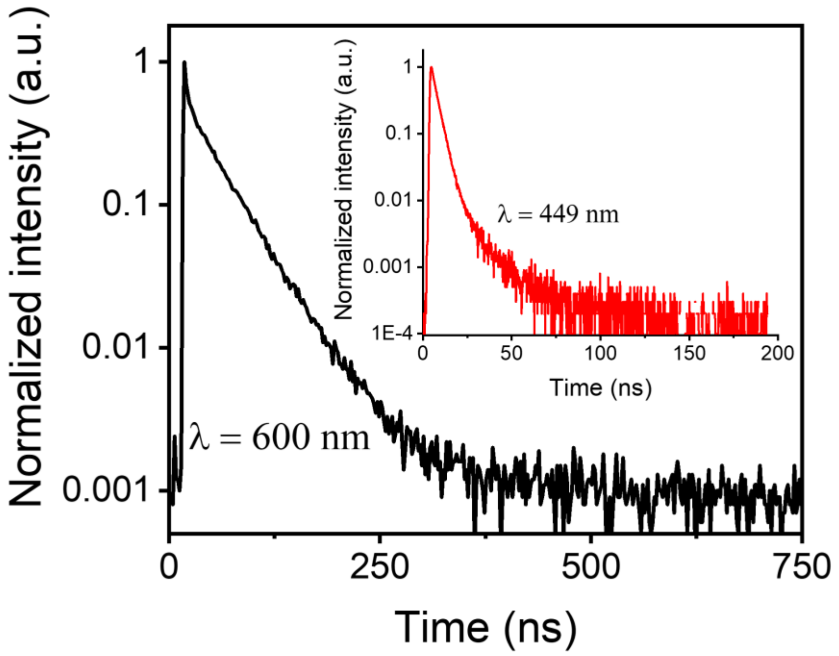
Normalized intensity (a.u.)
1
0.1
0.01
0.001
0
250
500
750
Time (ns)
λ = 600 nm
λ = 449 nm
1E-4
0
50
100
150
200

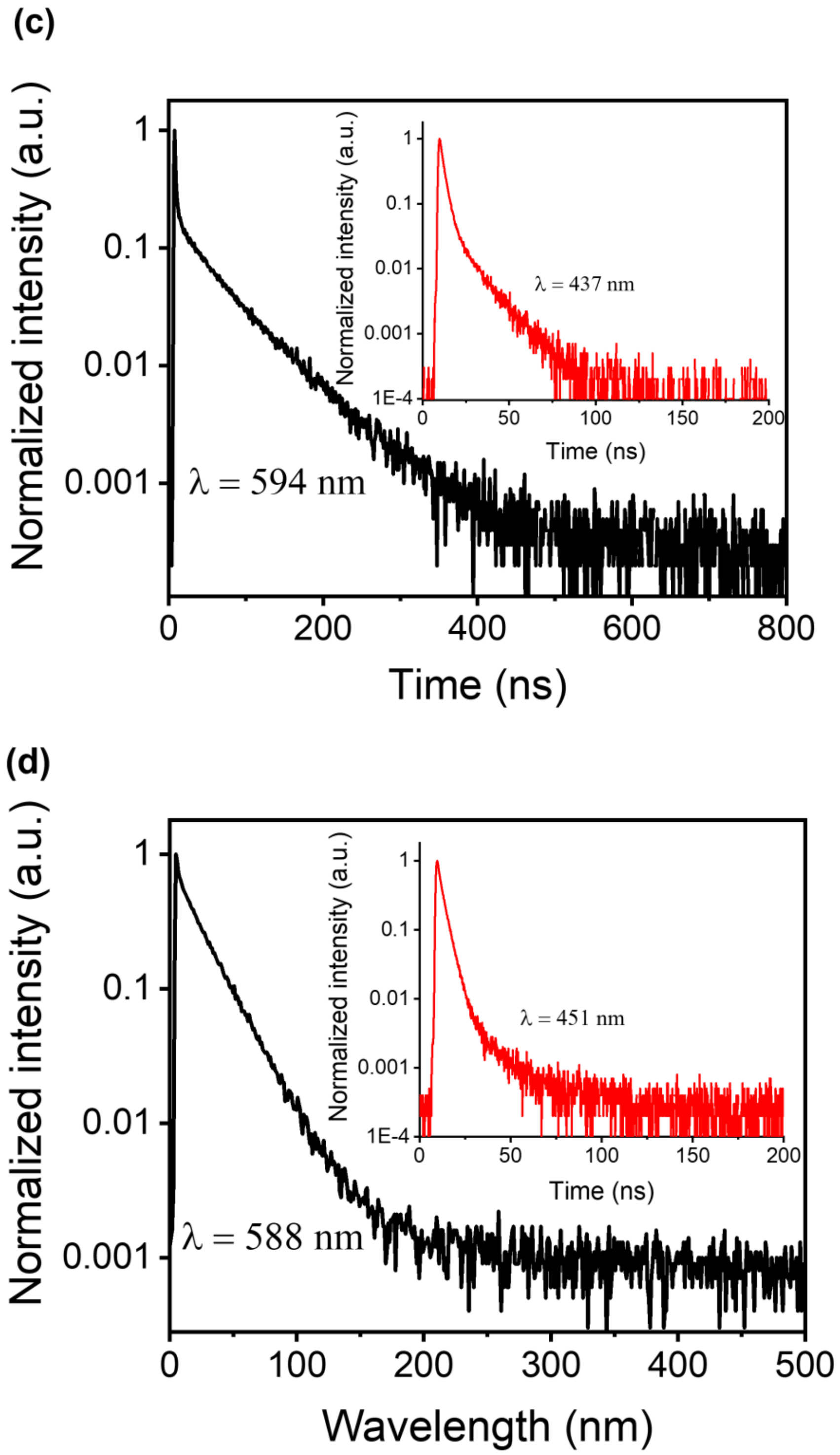

Figure S27. Transient PL decay curves of (a) **3a**, (b) **3b** and (c) **5a** (d) **5b** in THF solution at around 600 nm. The insets show that the luminance decay traces of emission band at around 450 nm. [**3a**] = [**3b**] = [**5a**] = [**5b**] = 1 × 10^{-3} M, λ_{ex} = 377.4 nm.

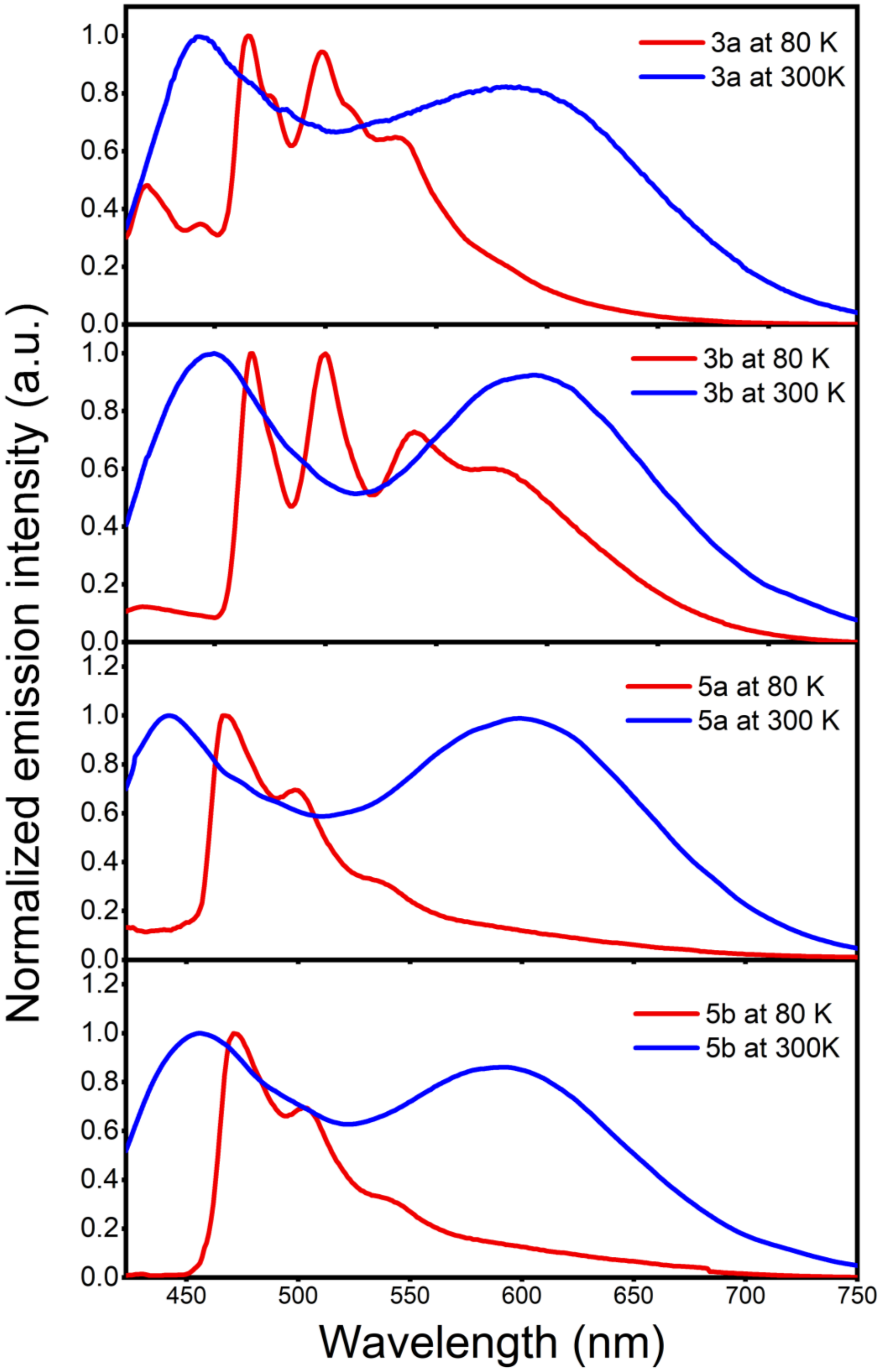

Figure S28. PL spectra of complexes **3a–3b** and **5a–5b** (top to bottom) in 2-MeTHF at 80 K and 300 K.

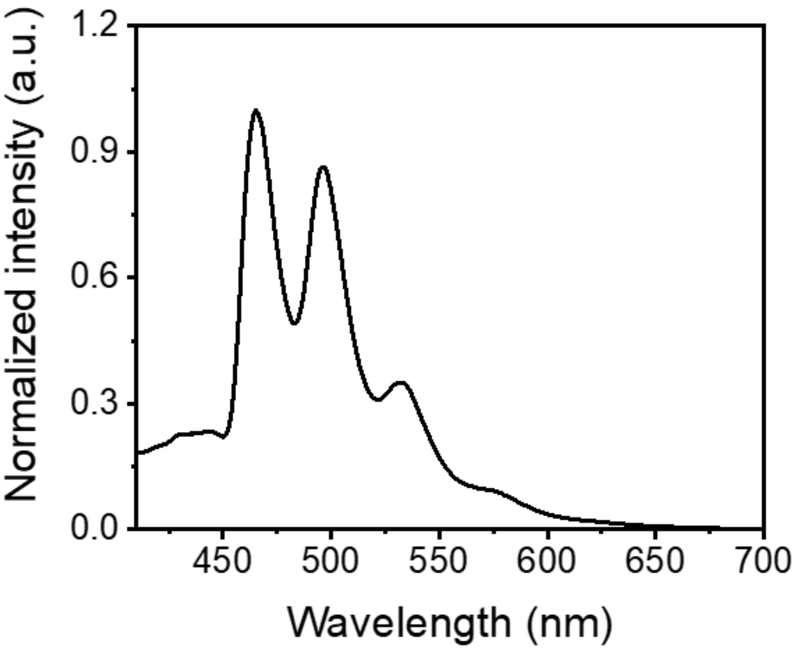

Figure S29. Emission spectrum of KCz at 80 K in 2-MeTHF, λ_{ex} = 380 nm.

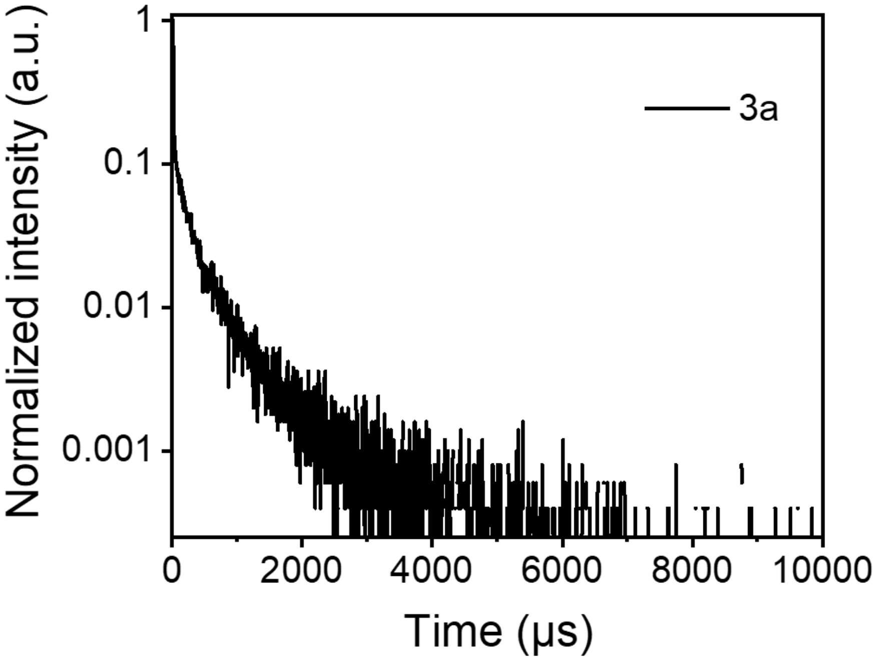

Figure S30. Transient PL decay curve of **3a** in 2-MeTHF at around 500 nm under 80 K, λ_{ex} = 380 nm.

Multicolor emission construction

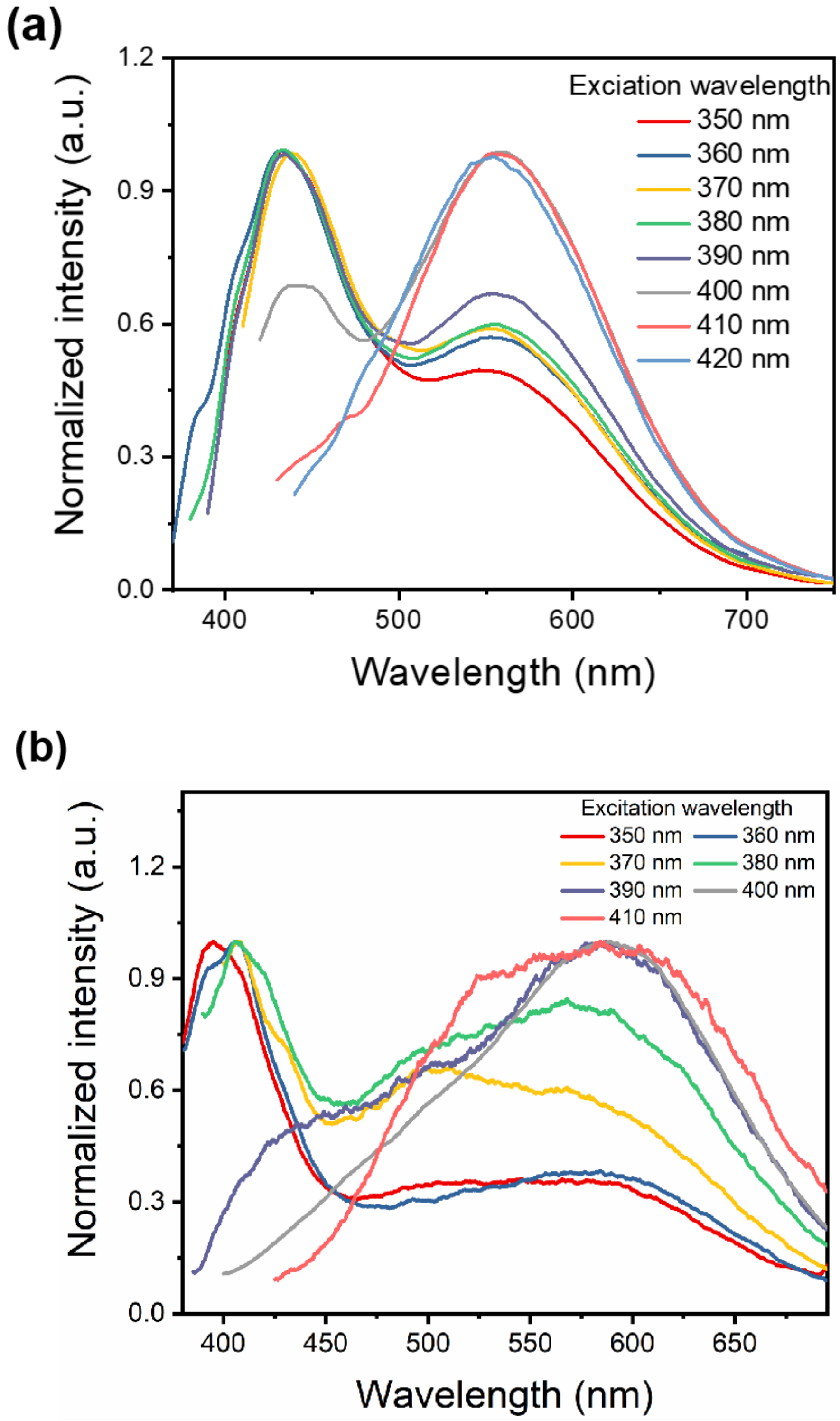

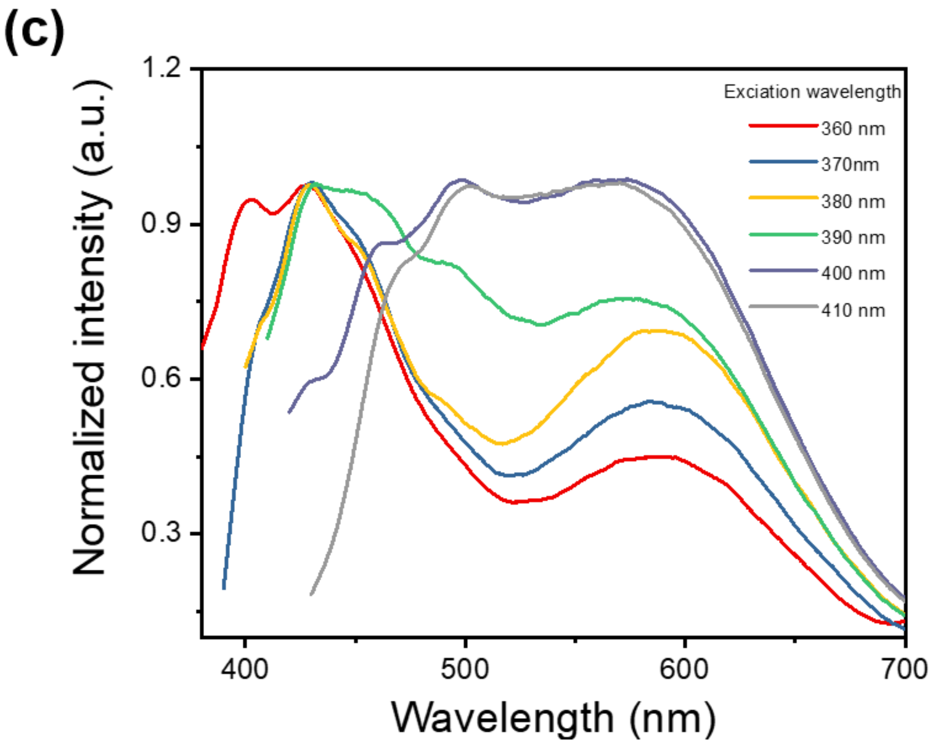

Figure S31. Emission spectra of (a) **3a**, (b) **5a** and (c) **5b** in THF solution using various excitation wavelengths, [**3a**] = [**5a**] = [**5b**] = 1 × 10^{-3} M, 25 ^{o}C.

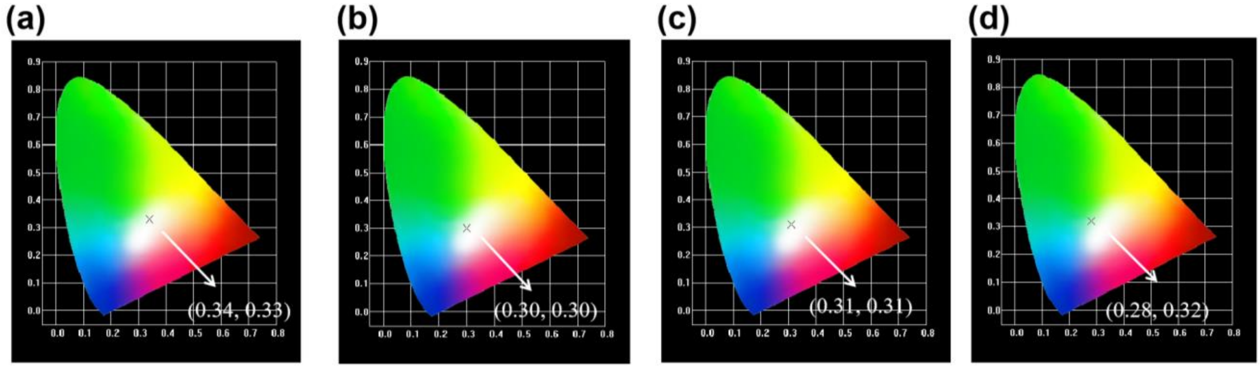

Figure S32. Commission Internationale de l'Éclairage (CIE) 1931 coordinates of PL emission: (a) **3a**; (b) **3b**; (c) **5a**; (d) **5b** in the 1 × 10^{-3} M THF solution under 375–400 nm excitation wavelengths at room temperature in the Ar atmosphere.

Electrochemical Characterization

Cyclic voltammograms were measured in dry THF solution (1×10^{-4} M **3a** and 0.2 M Bu_4NPF_6 as a supporting electrolyte) at an argon atmosphere. Ag/Ag^+ was used as a reference electrode, and a Pt wire and glassy carbon electrode served as the counter and working electrode, respectively. The data were gained versus ferrocene/ferrocenium (Fc/Fc^+) couple measured under the same conditions. As shown in Figure S34, the CV of **3a** displayed an irreversible curve, suggesting the complex is unstable on the CV timescale.

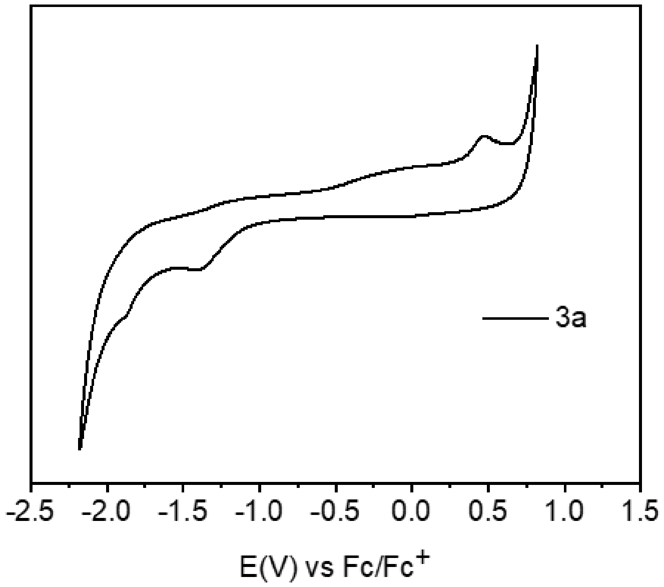

Figure S33. Cyclic voltammetry of complex **3a**.

NMR spectra characterization

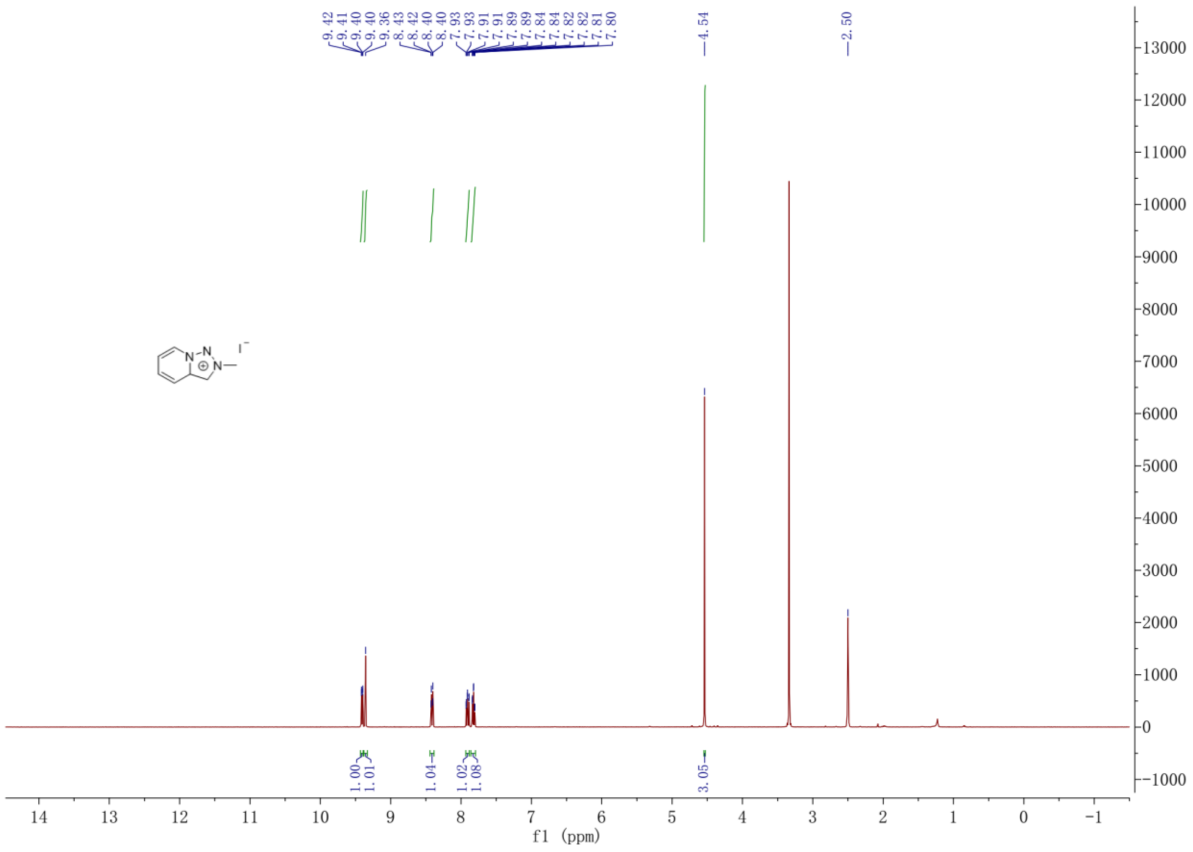

Figure S34. ^{1}H NMR spectrum of **1**.

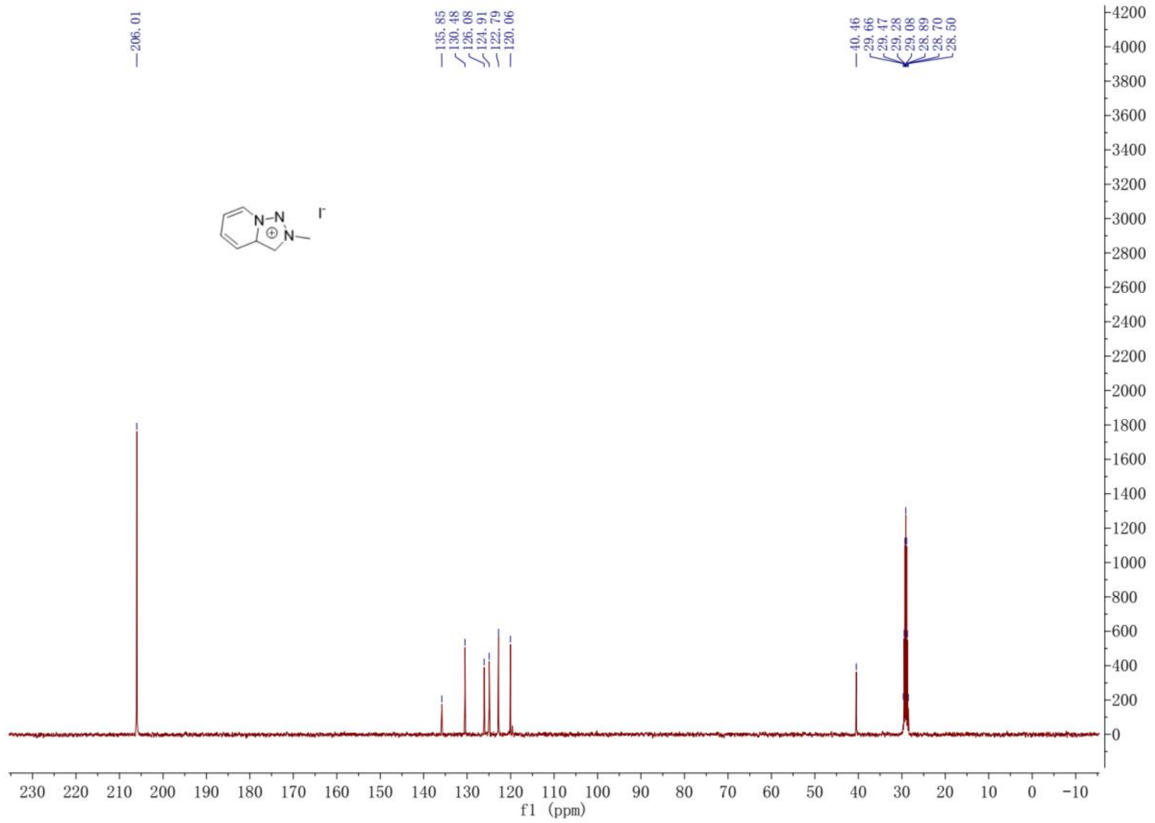

Figure S35. ^{13}C NMR spectrum of **1**.

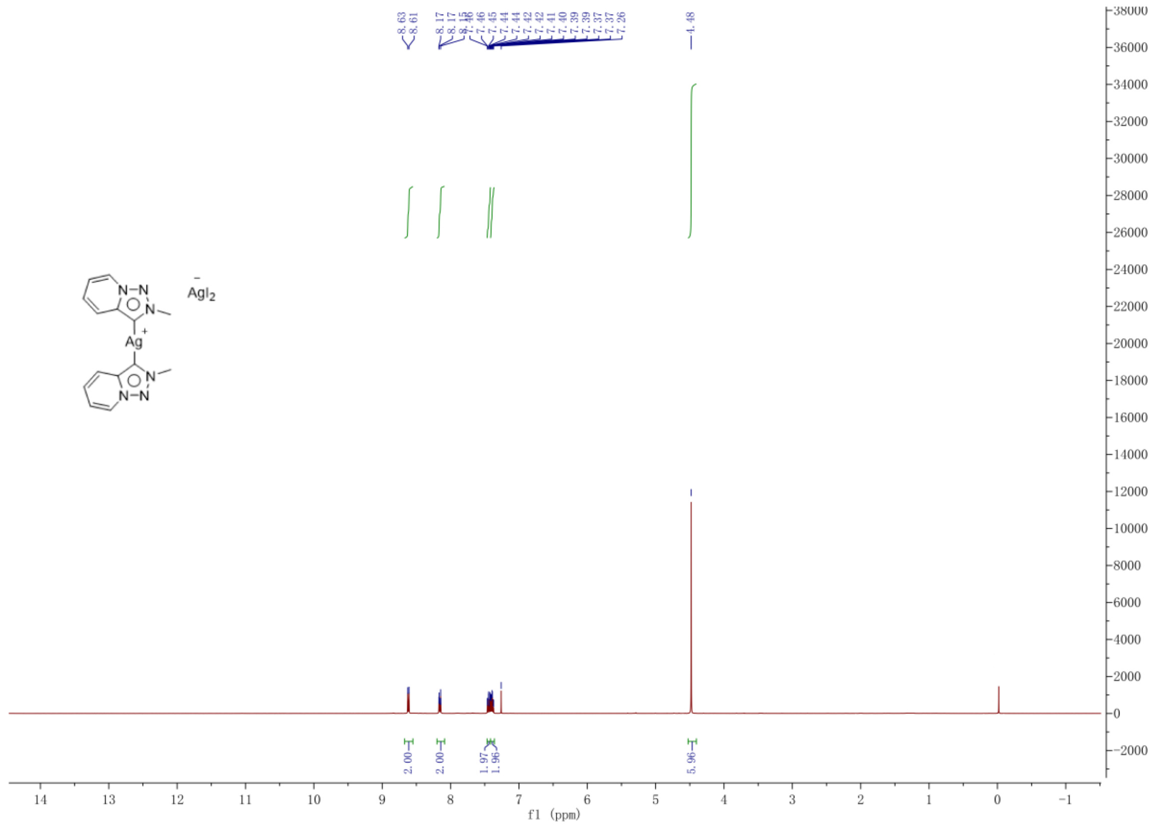

Figure S36. ^{1}H NMR spectrum of **6**.

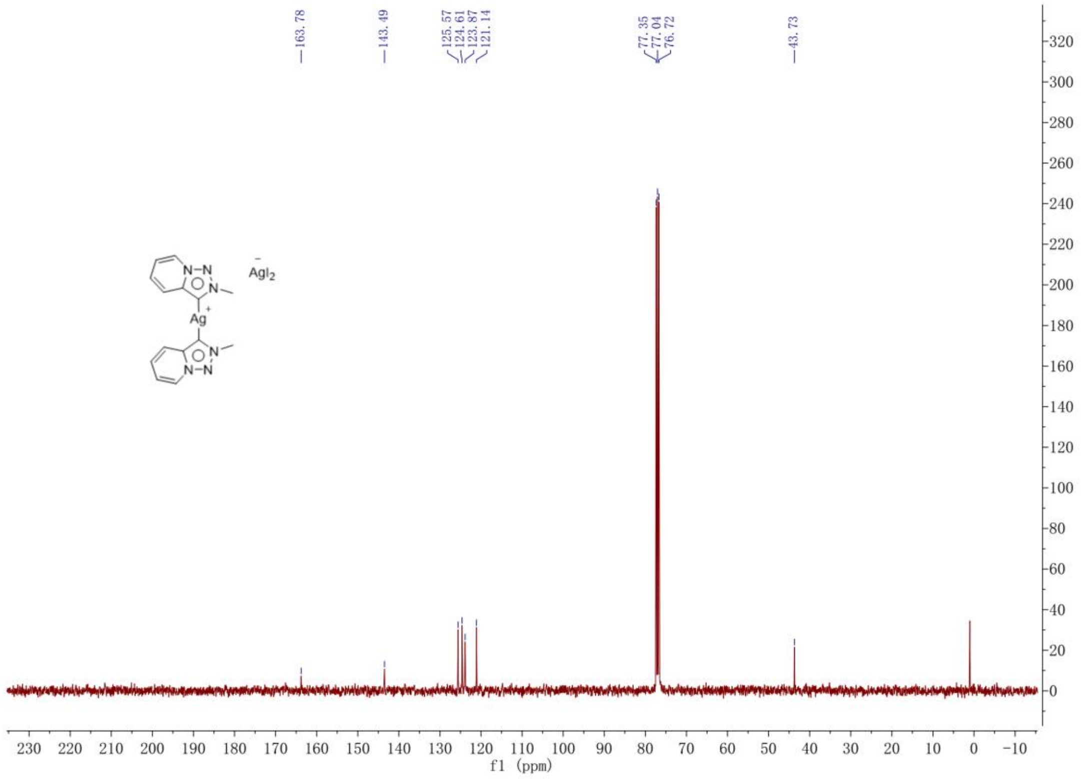

Figure S37. ^{13}C NMR spectrum of **6**.

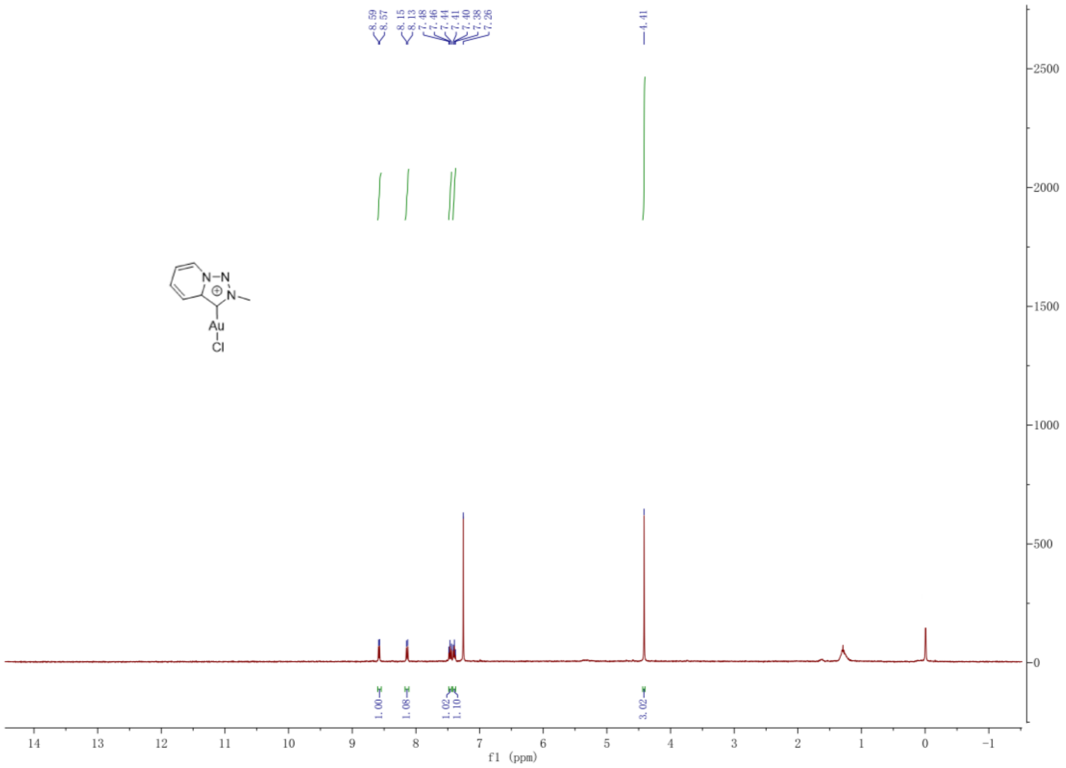

Figure S38. ^{1}H NMR spectrum of **2**.

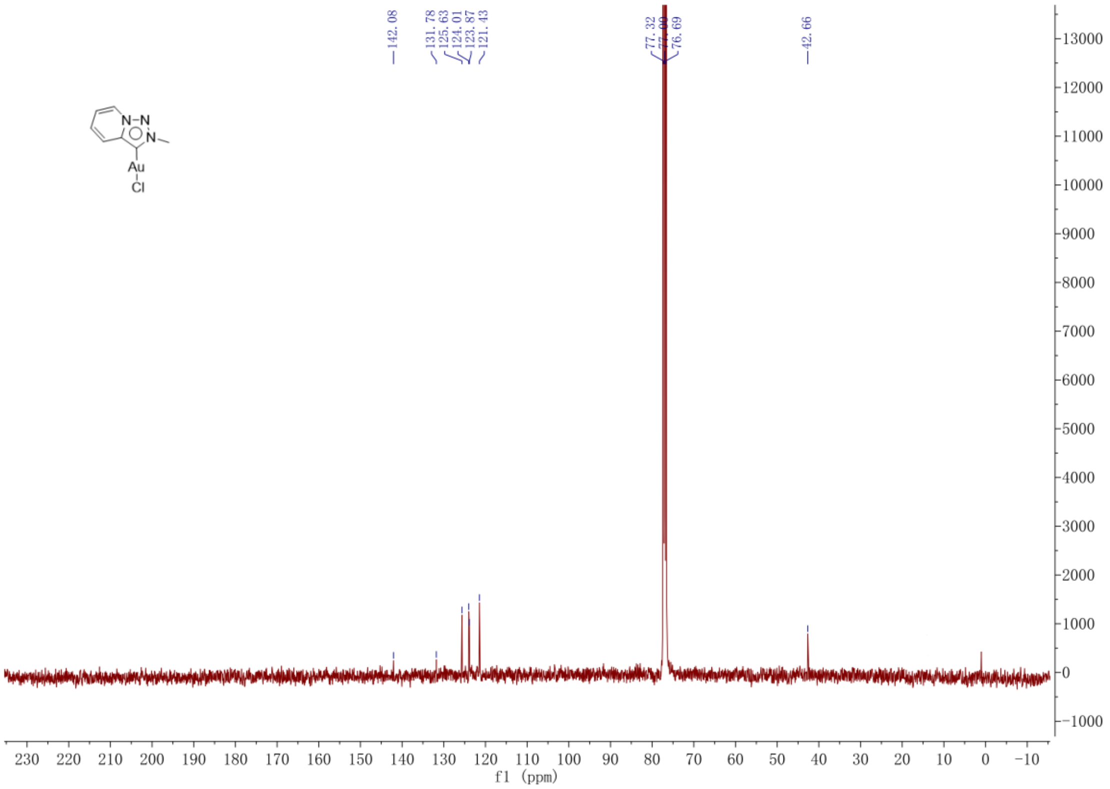

Figure S39. ^{13}C NMR spectrum of **2**.

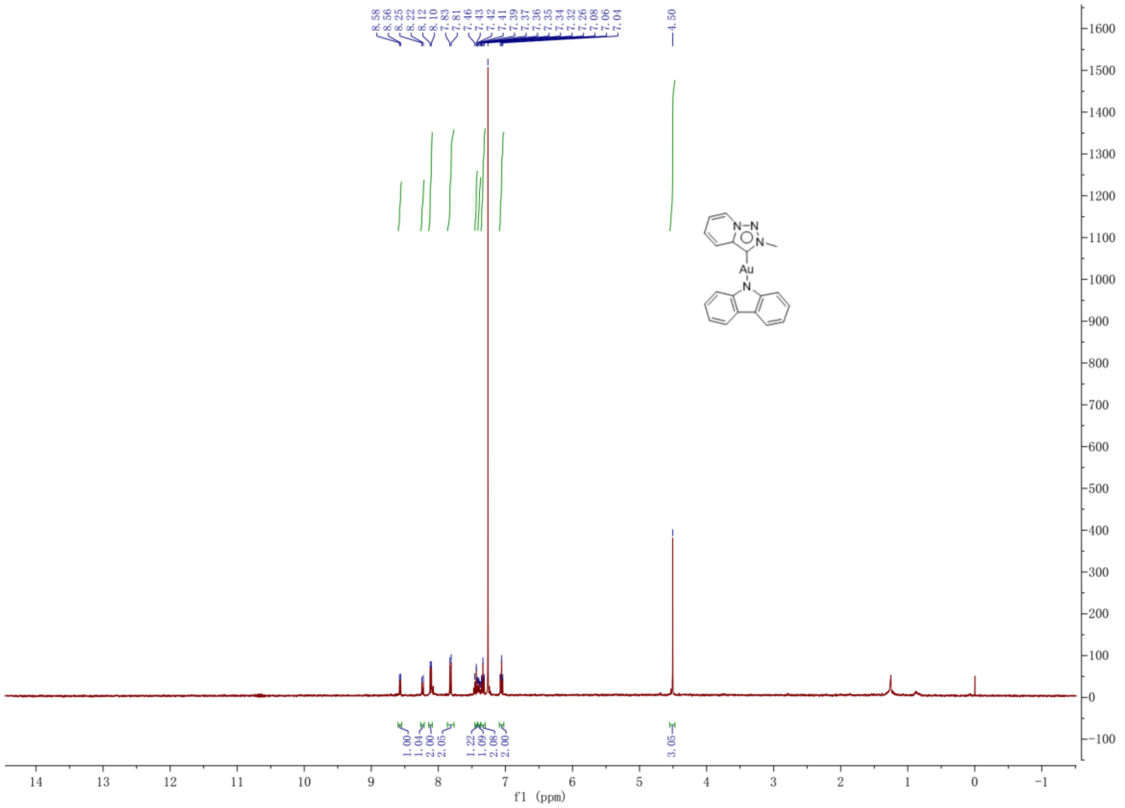

Figure S40. ^{1}H NMR spectrum of **3a**.

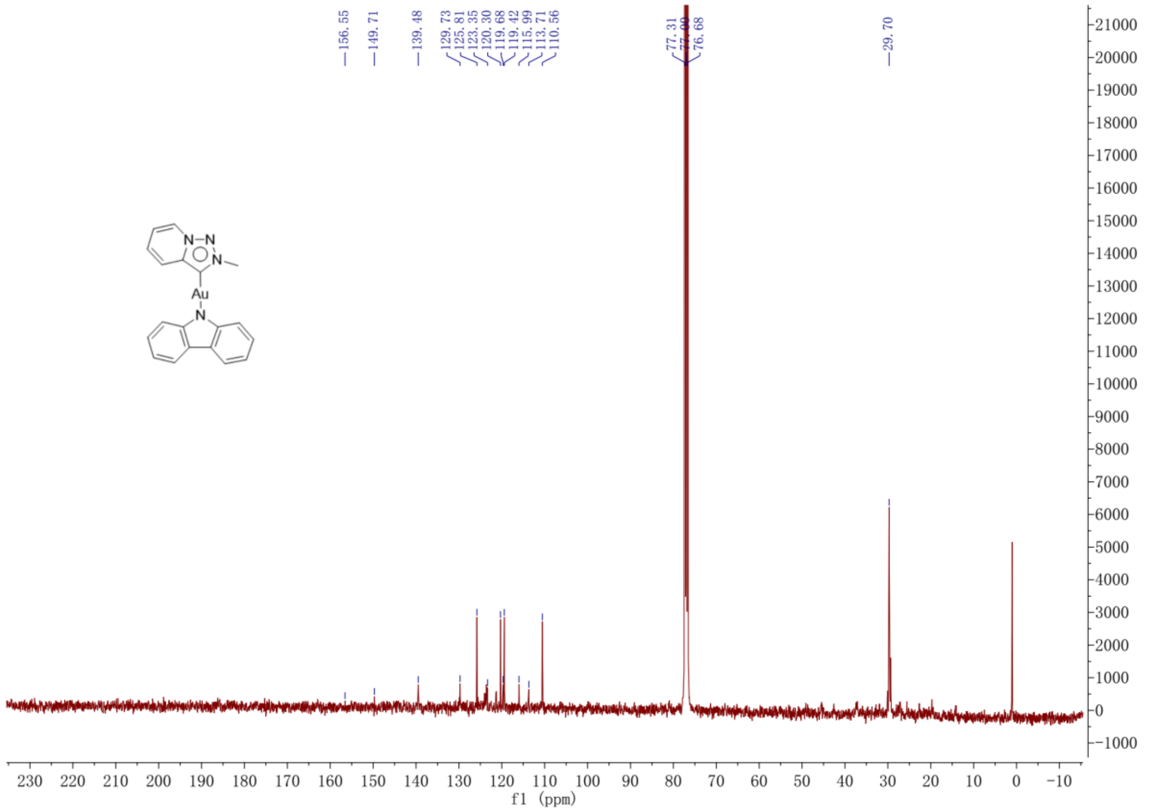

Figure S41. ^{13}C NMR spectrum of **3a**.

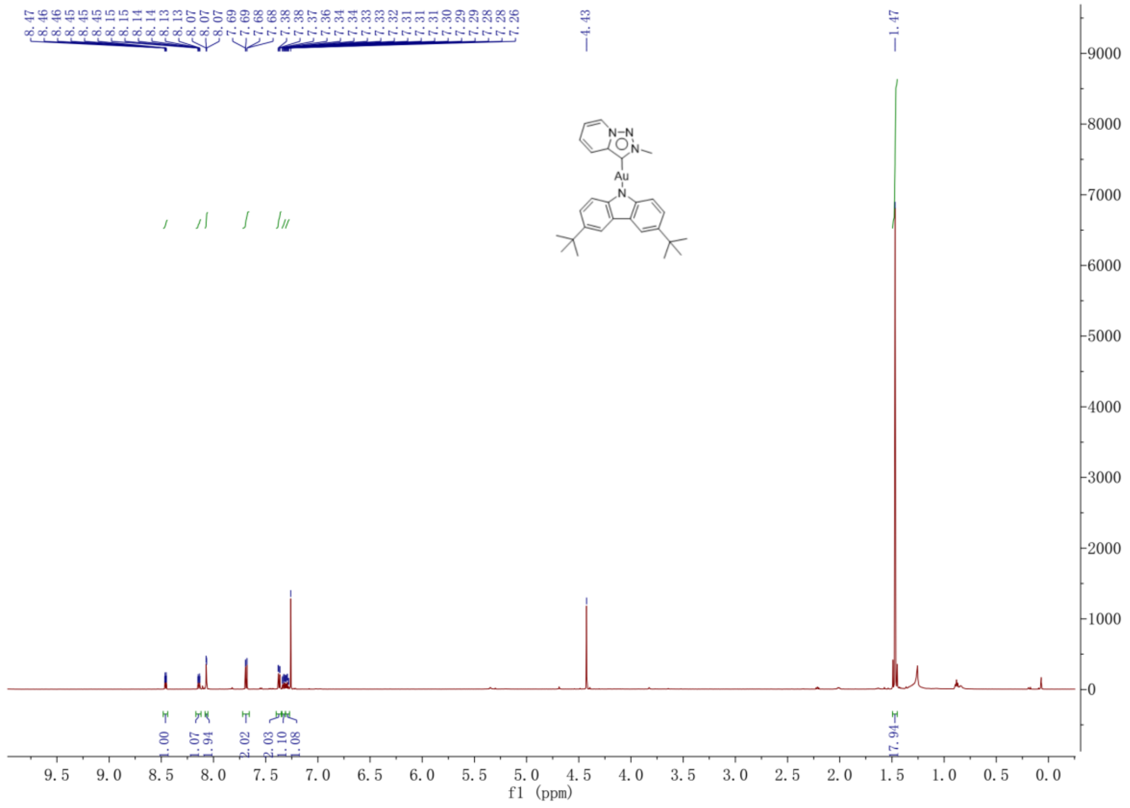

Figure S42. ^{1}H NMR spectrum of **3b**.

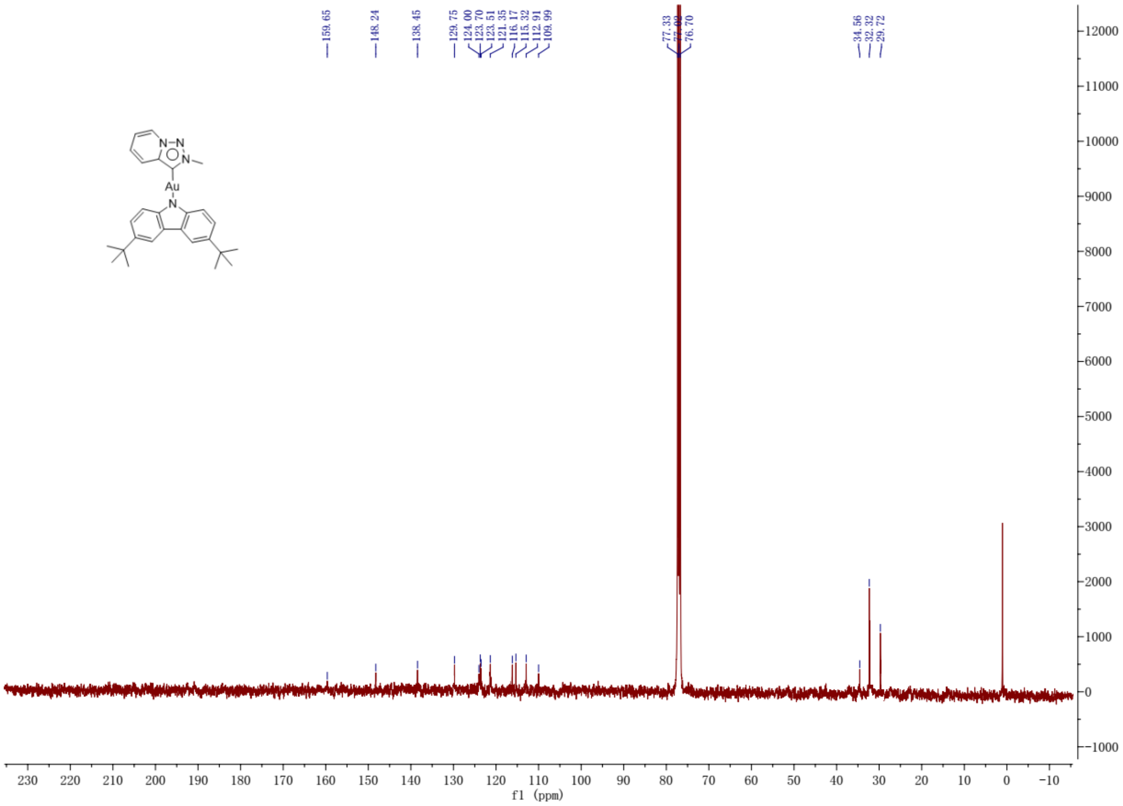

Figure S43. ^{13}C NMR spectrum of **3b**.

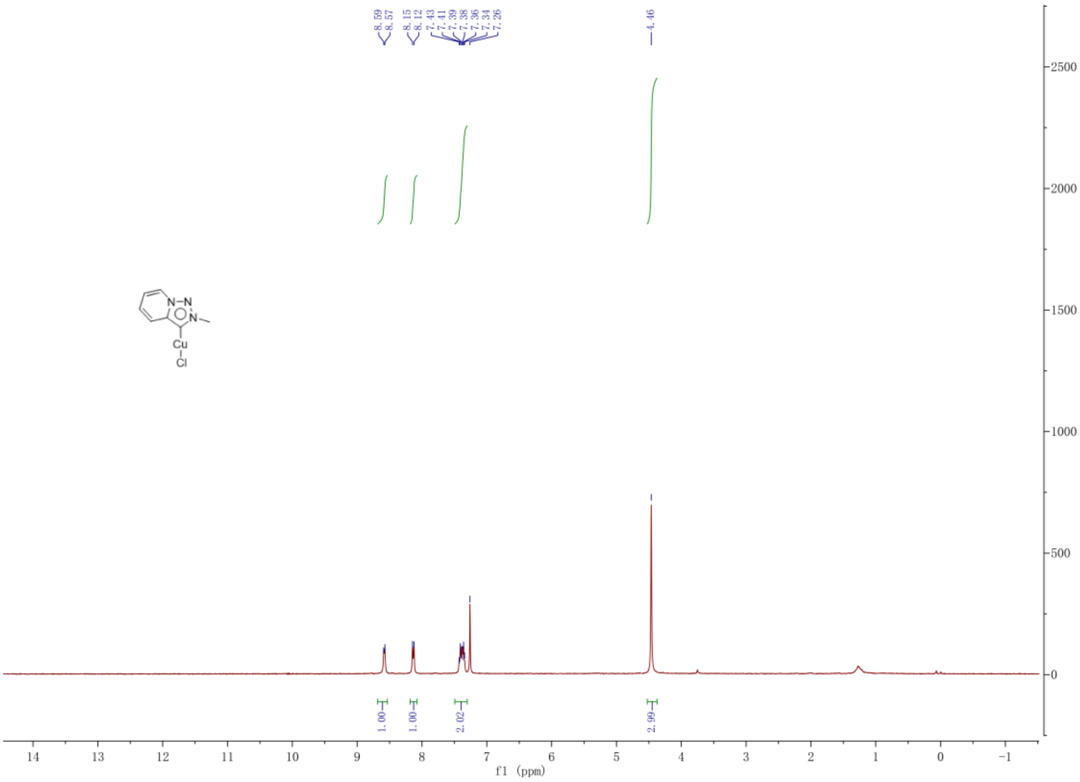

Figure S44. ^{1}H NMR spectrum of **4**.

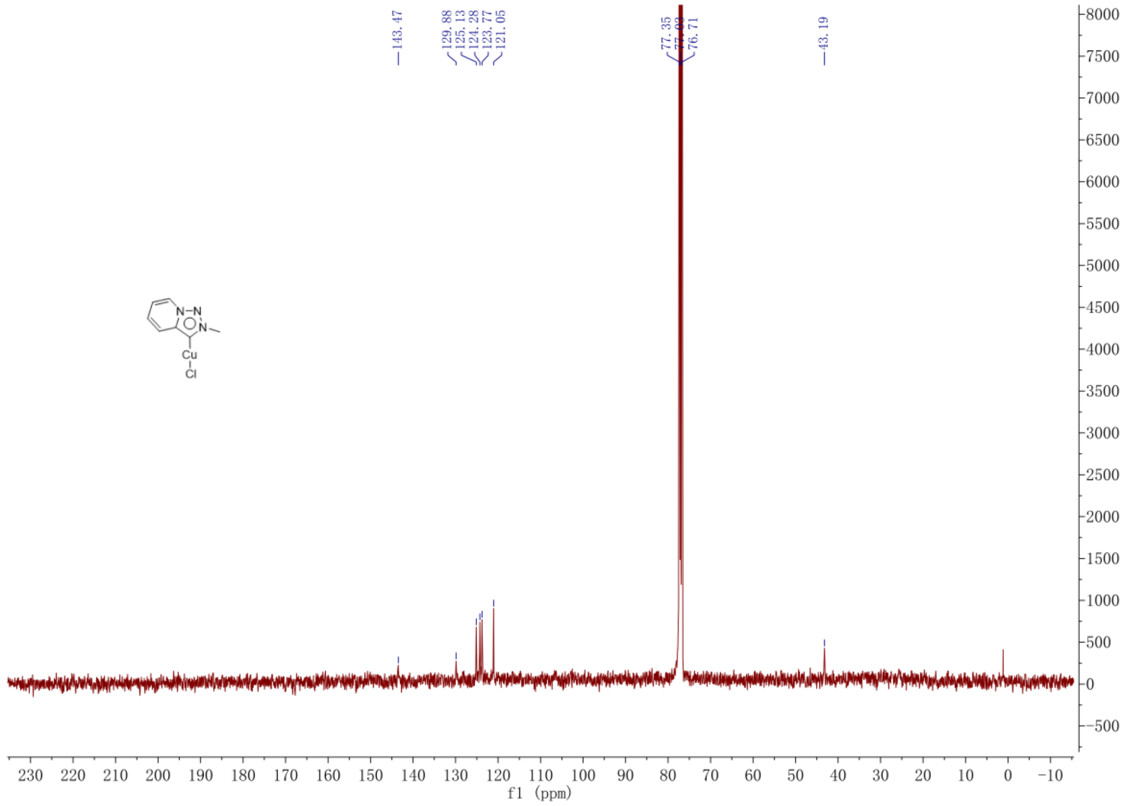

Figure S45. ^{13}C NMR spectrum of **4**.

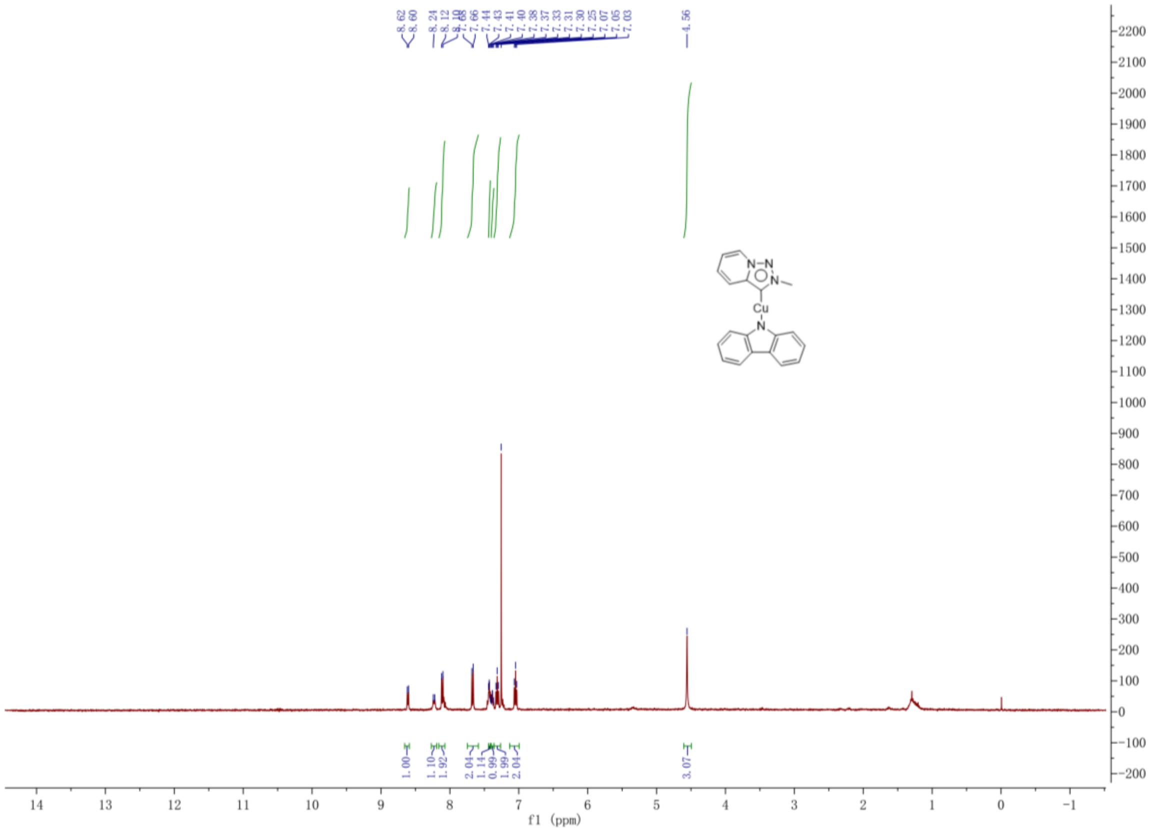

Figure S46. ^{1}H NMR spectrum of **5a**.

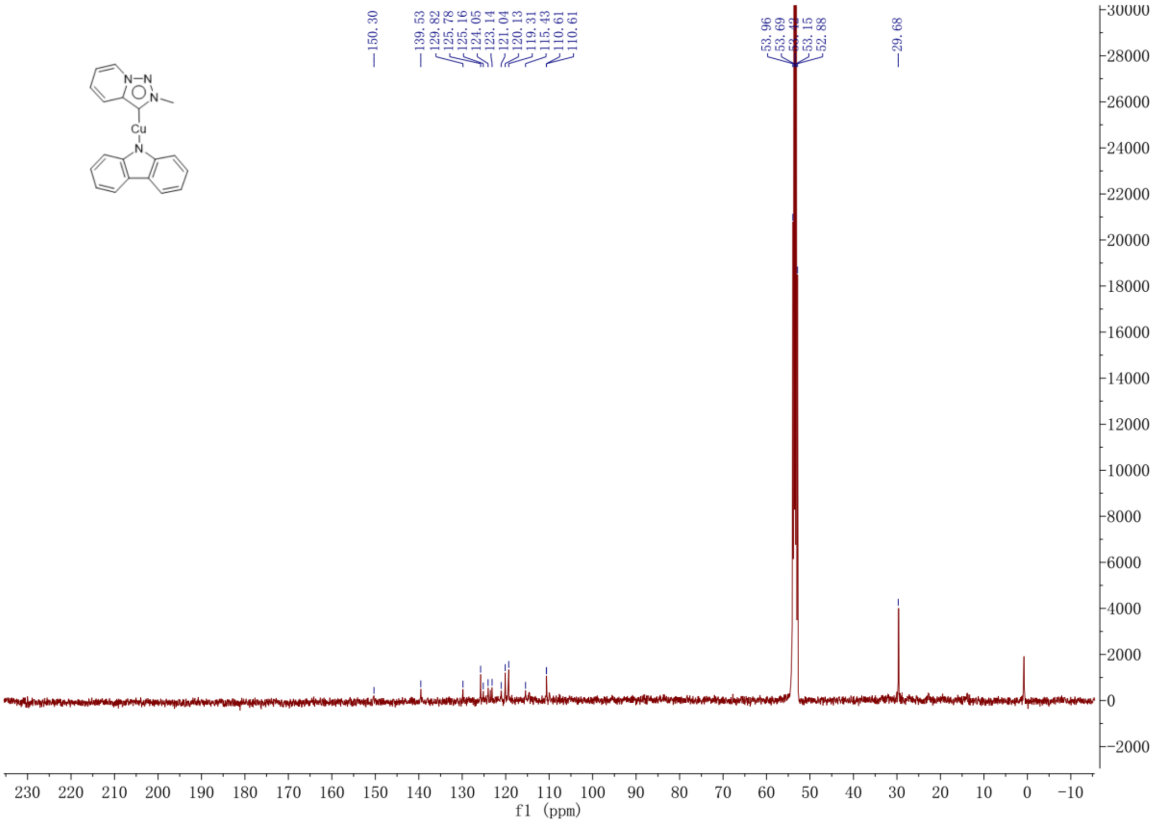

Figure S47. ^{13}C NMR spectrum of **5a**.

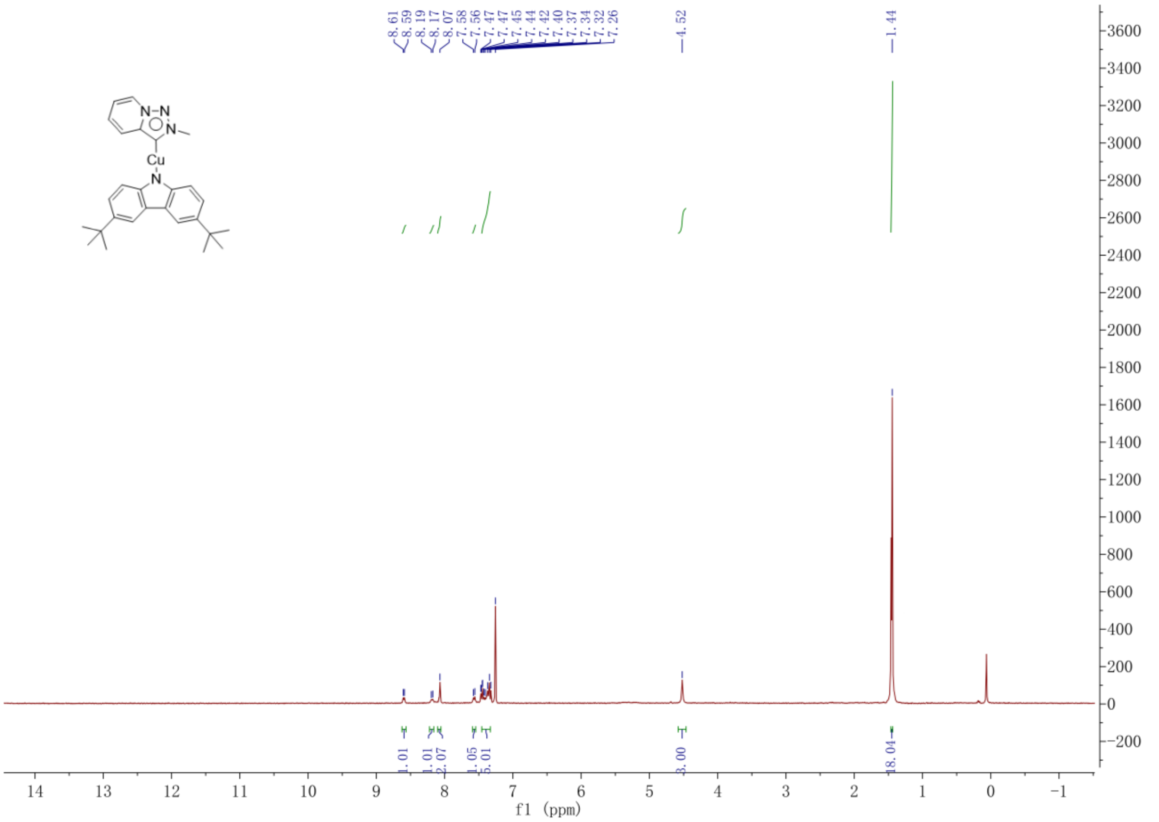

Figure S48. ^{1}H NMR spectrum of **5b**.

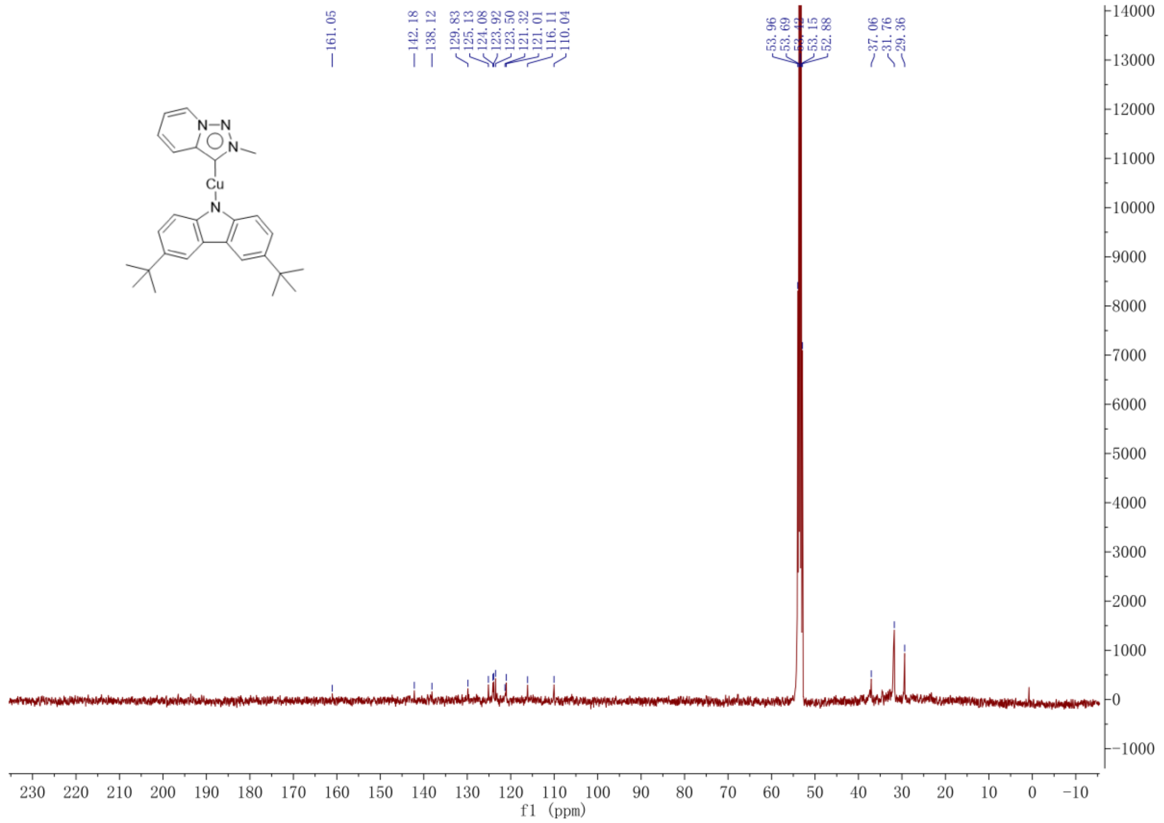

Figure S49. ^{13}C NMR spectrum of **5b**.

References

S1. Dolomanov, O. V.; Bourhis, L. J.; Gildea, R. J.; Howard, J. A. K.; Puschmann, H. OLEX2: a complete structure solution, refinement and analysis program. *J. Appl. Cryst.* **2009**, *42,* 339-341.

S2. Sheldrick, G. M. SHELXT - Integrated space - group and crystal - structure determination. *Acta Cryst*. A **2015**, *71,* 3-8.

S3. Sheldrick,G. M. Crystal structure refinement with SHELXL. *Acta Cryst*. C **2015**, *71,* 3-8.

S4. Hirayama, T.; Ueda, S.; Okada, T.; Tsurue, N.; Okuda, K.; Nagasawa, H. Facile One-Pot Synthesis of [1,2,3]Triazolo[1,5-*a*]Pyridines from 2-Acylpyridines by Copper(II)-Catalyzed Oxidative N–N Bond Formation. *Chem. Eur. J.* **2014**, *20,* 4156-4162.

S5. Korshin, E. E.; Leitus, G.; Shimon, L. J. W.; Konstantinovski, L.; Milstein, D. Silanol-Based Pincer Pt(II) Complexes: Synthesis, Structure, and Unusual Reactivity. *Inorg. Chem.* **2008**, *47,* 7177–7189.

S6. Gaussian 09, Revision D.01, Frisch, M. J.; Trucks, G. W.; Schlegel, H. B.; Scuseria, G. E.; Robb, M. A.; Cheeseman, J. R.; Scalmani, G.; Barone, V.; Mennucci, B.; Petersson, G. A.; Nakatsuji, H.; Caricato, M.; Li, X.; Hratchian, H. P.; Izmaylov, A. F.; Bloino, J.; Zheng, G.; Sonnenberg, J. L.; Hada, M.; Ehara, M.; Toyota, K.; Fukuda, R.; Hasegawa, J.; Ishida, M.; Nakajima, T.; Honda, Y.; Kitao, O.; Nakai, H.; Vreven, T.; Montgomery, J. A.; Jr.; Peralta, J. E.; Ogliaro, F.; Bearpark, M.; Heyd, J. J.; Brothers, E.; Kudin, K. N.; Staroverov, V. N.; Keith, T.; Kobayashi, R.; Normand, J.; Raghavachari, K.; Rendell, A.; Burant, J. C.; Iyengar, S. S.; Tomasi, J.; Cossi, M.; Rega, N.; Millam, J. M.; Klene, M.; Knox, J. E.; Cross, J. B.; Bakken, V.; Adamo, C.; Jaramillo, J.; Gomperts, R.; Stratmann, R. E.; Yazyev, O.; Austin, A. J.; Cammi, R.; Pomelli, C.; Ochterski, J. W.; Martin, R. L.; Morokuma, K.; Zakrzewski, V. G.; Voth, G. A.; Salvador, P.; Dannenberg, J. J.; Dapprich, S.; Daniels, A. D.; Farkas, O.; Foresman, J. B.; Ortiz, J. V.; Cioslowski, J.; Fox, D. J. Gaussian, Inc., Wallingford CT, **2013**.
